# Supplementary material for: A soccer-based intervention improves incarcerated individuals’ behaviour and public acceptance through group bonding
Source: Nat Hum Behav. 2024 Oct 14;8(12):2304–13. doi: 10.1038/s41562-024-02006-3 (PMC11659165; doi:10.1038/s41562-024-02006-3)
Supplement: Supplementary file 1 — Samples, materials, procedures and analyses of studies 1 (A) to 9 (I), including Supplementary Tables A1–21, B1–6, C1–8, D1, E1 and 2, F1 and 2, G1 and 2, H1 and 2 and I1 and 2 and Fig. C1. [file 41562_2024_2006_MOESM1_ESM.pdf]

nature human behaviour

Supplementary information

https://doi.org/10.1038/s41562-024-02006-3

A soccer-based intervention improves incarcerated individuals' behaviour and public acceptance through group bonding

In the format provided by the authors and unedited

Contents

Supplementary Information A (Study 1): The impact of Twinning Project on prison behaviour 3

Information about the Twinning Project 3

Sample 3

Materials 4

Analyses 5

Supplementary Information B (Study 2): The impact of identity fusion to positive and negative social groups on future optimism and prison behaviour among Twinning Project participants 52

Sample 52

Materials 52

Analyses 53

Supplementary Information C (Study 3): Associations between fusion to positive/negative groups and criminal behaviour among formerly incarcerated people 59

Sample 59

Materials 59

Analysis 61

Supplementary Information D (Study 4): Willingness to hire formerly incarcerated people based on prison education & shared interests among the receiving community 76

Sample 76

Materials 76

Procedure 78

Analyses 78

Supplementary Information E (Study 5): Fusion and willingness to hire/perceived chances to desist among UK soccer fans 82

Sample 82

Materials 82

Analyses 82

Supplementary Information F (Study 6): Fusion and willingness to hire/perceived future chances among US American Football fans 86

Sample 86

Materials 86

Analyses 86

Supplementary Information G (Study 7): Fusion and willingness to hire/ perceived future chances among US nationals 90

Sample 90

Materials 90
Analyses 90
Supplementary Information H (Study 8): Fusion and willingness to hire among UK nationals 94
Sample 94
Materials 94
Analyses 94
Supplementary Information I (Study 9): Fusion and willingness to hire among US nationals 97
Sample 97
Materials 97
Analyses 97
Supplementary Information Reference List 100

Supplementary Information A (Study 1): The impact of Twinning Project on prison behaviour

Information about the Twinning Project

(based on the Template for Intervention Description and Replication, TiDierR (Hoffman et al., 2014), https://www.equator-network.org/reporting-guidelines/tidier/)

1. The Twinning Project (TP) is a sport-based intervention that pairs prisons with local professional sports teams (association football, rugby) to provide entry-level accredited coaching or refereeing courses to prisoners. The intervention is provided in adult-only male and female prisons in England and Wales, including all security categories. The goal of TP is to increase participants' self-esteem and employability and thereby reduce reoffending rates after release, as well as to improve behaviour and wellbeing while incarcerated.

2. Programmes are delivered face-to-face by teams consisting of at least one prison officer (typically a Physical Education Instructor) and one member of staff of the sports club (a coach) and typically takes between 1 - 12 weeks (minimum 5 sessions) to complete. Individual sessions are delivered face-to-face to groups of about 10-18 prisoners, using the facilities available in the respective prisons, which may or may not include outdoor facilities. The most common version of the programme provides an entry level football coaching qualification, which consists of both theoretical and practical sessions. Materials reflect the official FA 'Introduction to coaching football' course, but coaches have the ability to adjust materials according to cohort needs (e.g., shorten text on slides, bring additional material for illustration purposes).

3. Cohorts are self-selecting: participants apply to take part in the Twinning Project, which is advertised and tends to be well known in the prison gym and in other areas of the prison. Applications are assessed by prison staff, then the club delivering the programme. There is usually a waiting list to take part in a programme. Participants should have little time left to serve and most clubs do not recruit participants with sexual offences.

Sample

Data was collected from the population of HMPPS (His Majesty's Prison and Probation Service) UK adult prisons (men and women), including all prisoners enrolled in the Twinning Project (treatment group) between September 2021 - March 2023 and a matched control group. In this study we are working exclusively with the male population, due to the unique needs of the women's population and highly unbalanced sample sizes (women make up around 5% of the prison population (House of Commons Justice Committee, 2022)).

Initially, 1411 individuals were identified as participants enrolled in the Twinning Project (TP) programme within the predetermined research period (September 2021 – March 2023), based on participant lists provided by HMPPS, as well as responses to a longitudinal survey conducted among a subsample of TP cohorts. Prison behaviour data was requested for n = 1348 cases, (excluding 51 cases lacking personally identifiable information, and 12 cases who did not complete the programme within the research period). HMPPS was able to identify and provide the data of n = 996 individuals. Among this sample, 67 cases had a recorded age of younger than 18 at the time of starting the programme or lacked reliable indicators that they were part of adult-cohorts and were removed from further analyses. Of the remaining n = 927 cases, 93 were female (10%), and preliminary analyses showed substantial differences between genders in terms of baseline levels of prison behaviour and experiences in the course. Considering the unique circumstances and needs of female prisoners, we decided to analyse their data separately and focus the subsequent analyses on the data of n = 834 male participants.

Full details about outcome measures is provided in 'Materials'. However, it should be noted that analyses taking into account prison behaviours captured via positive and negative case notes, a quantitative variable reflecting

the number of qualitative notes made by prison officers, exclude a subsample of $n = 158$ individuals housed in privately run prisons due to fact that these institutions prioritise a different system to record case notes (to which we had no access). The available data would underestimate such outcome measures compared to public prison records. Indeed, independent sample t-tests showed significantly lower baseline averages of positive (t(428.74) = -7.50, $p < .001$) and negative case notes (t(336.58) = - 2.73, $p = .007$) from private prison cases compared to public prison cases, whereas there were no significant differences for the baseline average of proven adjudications (t(832) = 0.42, $p = .673$), an outcome that is captured using the same system between private and public prisons.

Materials

Prison behavioural data is routinely collected by HMPPS for each prisoner. We obtained access to six behaviours: number of positive and negative case notes written by prison officers, visitation attendance, job attendance, number of self-harm incidents, and number of adjudications (i.e., disciplinary actions in response to crimes committed/rules broken in prison that require an external judge to rule on). Of these, adjudications is the most robust and objective measure (McDougall et al., 2017). For the case notes we calculated a balance score by subtracting the number of negative case notes from the positive case notes, so that positive numbers indicate an overall positive case note balance. For the activity attendance variable we intended to calculate an attendance rate based on the number of registered activity attendances out of all registered activities (including recorded absences). However, over 50% of cases contained missing data on either attendances or absences within pre-and post-treatment periods. The prevalence of missing data correlated significantly with individual institutions, as well as prison security levels and the observation period start date, which could reflect differences in recording practices between prisons and over time, and this data was therefore not included in the final analyses.

Additionally, HMPPS provides data on demographic characteristics (age, gender, ethnicity) and criminal justice records, including prisoners' Incentives and Earned Privileges (IEP) level (an incentive scheme reflecting prison behaviour, categorised as 1 = basic, 2 = standard, 3 = enhanced (Ministry of Justice, 2022)), prison, index offence (i.e., original offence for sentence, coded according to ORGS 4.0, see Howard, 2015), time left to serve, total number of convictions, and time since first offence. The latter two variables were used to calculate the Copas rate, an index designed to capture the length and intensity of a criminal career (Copas & Marshall, 1998), using the following formula: the natural log of (the number of court appearances or cautions + 1) / (the length of criminal career in years, +10).

$$copas = \ln\left(\frac{\text{the number of court appearances or cautions } + 1}{\text{the length of criminal career in years } + 10}\right)$$

Control group

HMPPS initially provided a group of $n = 5419$ control cases which were selected to reflect the treatment group based on average age, as well as gender, ethnic background and prison type composition. Following the abovementioned exclusion of female prisoners and prisoners from privately run institutions, $n = 4334$ cases were theoretically available for comparison.

We further restricted the observation periods according to the research period, i.e., pre-treatment period ends before 01.09.2021 and post-treatment period starts no later than 01.03.2023, which was important given the unique time-frame of the data collection during the later stages of the COVID-19 pandemic (Hewson et al., 2020; Suhomlinova et al., 2022). A sample of n = 1874 control cases contained data for this period and were used for the main analyses.

The matching parameters included baseline level prison behaviours captured in a two-month period before the treatment start date. Thus, we had to determine an equivalent time-frame for control cases in order to process the baseline data. We used the average time between treatment start and release for treatment cases (20 months) to calculate the equivalent pre/post-treatment periods for control cases. Thereby, we eliminated the variation in the time left to serve of the control group, a potential problem if people are often motivated to behave 'well' toward the end of their sentence. We examine alternative approaches to process the data and estimate the propensity score models as part of the sensitivity analyses found in *Sensitivity Analyses* section starting on p. 20, namely: using the median time to release as the control group pre/post cut point; and allowing the cut point to vary randomly.

Analyses

Deviation from pre-registration

The pre-registration did not specify an approach to process case-control data because it was originally anticipated that the data provider would supply a 1:1 matched control sample. Due to unforeseen circumstances this was not possible. The data provider agreed to supply a larger control population, loosely pre-selected to meet the demographic composition of the treatment population (i.e., similar % male/female cases, similar mean age, similar % ethnic composition), for which we chose the analysis approach as described below. Consequently, the pre-registered analysis of simple independent sample t-tests was abandoned and the treatment effect was determined in line with the TWANG approach (Ridgeway et al., 2021) outlined below.

The pre-registered analysis of '*activity/ work attendance'*- rates and '*visit attendance*'-rates was not conducted as it was deemed not feasible. The supplied data did not allow to distinguish between types of events attended, and there were concerns over data quality (due to non-random missingness, as outlined above).

Differences between groups

Considering that the Twinning Project tends to recruit at a specific population of prisoners (younger, well-behaved), we decided to use the Toolkit for Weighting and Analysis of Nonequivalent Groups (TWANG) (Ridgeway et al., 2021) to balance treatment and comparison group responses by applying weights based on a propensity score (King & Nielsen, 2019). With the TWANG package in R, the propensity score and weights are generated using gradient boosted regression (GBM) models to either estimate the average treatment effect of the treated (ATT) or to estimate the population average treatment effect (ATE). As per Ridgeway et al. (2021:2) "ATE estimates the change in the outcome if the treatment were applied to the entire population versus if the control were applied to the entire population. ATT estimates the analogous effect, averaging only over the treated population effect." Given that TP, as currently designed, draws on a subset of the prison population, selected partially based on their suitability for the programme, an ATT analysis was deemed more appropriate than an ATE analysis.

The propensity score model was fit to predict membership in the treatment group (Twinning Project) based on demographic factors (age, ethnicity), institutional factors (prison security level) as well as indicators of prison behaviour (pre-treatment IEP-level, adjudications, case note balance, self-harm incidents) and criminal history (index offence, COPAS score). This approach thus at least somewhat eliminates issues of officer judgements in the selection process. We used the default settings of the *ps* function of the TWANG package unless specified otherwise (a sample of our code is provided in the supplementary data folder).

All models were run twice, following an intent-to-treat (ITT) and a protocol-adherence (PA) approach. Following Early et al.'s (2013) operationalisation, we define ITT as any cases admitted to the programme with the intention to receive treatment regardless of completion status. Consequently, PA was defined as any cases admitted to the programme who completed the treatment.

To evaluate the performance of the models, we compare standardised mean differences (SMD) and distribution differences (Kolmogorov-Smirnov statistic, KS) of all covariates between groups, with and without weights applied. We consider the following thresholds for imbalance; SMD > 0.20, KS > 0.10, (as per McCaffrey et al., 2013). The weights were estimated according to a stopping rule to determine the optimal iteration that minimizes imbalances between treatment groups. Stopping rules were either based on summary statistics of absolute standardised bias, or on the distribution of covariates between treatment groups. Here we use a stopping rule based on mean standardised bias (SMD), which has been shown to be particularly suitable to assess balance in binary treatment settings (see McCaffrey et al., 2013).

Summary statistics for group balance (Table A1) showed standardised mean differences above the threshold of SMD > 0.20, and average KS statistic close to the threshold of KS > 0.10 for the unweighted estimates average. The weighted estimates, based on the *es.mean* stopping method, showed improved balance, as indicated by average SMD and KS levels well below the thresholds. Furthermore, the summary table shows that the weighted estimates effectively utilize only 223 out of 1874 (12%) of the control cases, indicating that most control cases were unlike the treatment group which was unsurprising given the recruitment specifications of the programme.

Table A1

Summary of Balance Indicators

	Treatment n	Control n	Control Effective sample size	Max. SMD	Mean SMD	Max. KS	Mean KS	Iterations
ITT								
Unw.	676	1874	1874	1.20	0.26	0.56	0.09	
Weigh.	676	1874	223.65	0.22	0.06	0.09	0.02	2673
PA								
Unw.	621	1874	1874	1.17	0.26	0.56	0.09	
Weigh.	621	1874	223.77	0.23	0.06	0.09	0.02	2168

Note. Results of group balance testing based on mean standardised bias stopping rule for the intent to treat approach and protocol adherence approach.

ITT = Intent to Treat approach, PA = Protocol Adherence approach, Unw = un-weighted, Weigh. = weighted, SMD = standardised mean difference, KS = Kolmogorov Smirnov statistic.

The individual balance indicators in the unweighted comparison model showed significant differences between the groups for all covariates regarding SMD statistics (see Table A2). The treatment group was on average significantly younger, and more ethnically diverse. The treatment group also represented significantly more category D and fewer Category B prisoners, as well as significantly more prisoners on the highest (most desirable) IEP-level. This indicates that more prisoners were housed in lower risk prisons (category A being the most secure). They also had more favourable baseline case notes balances and fewer baseline self-harm incidents. There were also a number of significant difference regarding index offences, most notably, there were fewer sexual offences and more drug offences among the treatment group.

Examining the balance statistics of the parameters independently (Table A2) showed that SMD-levels were statistically significant for the shares of missing values (NAs) of IEP-level, Copas-score and case notes balance, and only for IEP-level was the standardised difference above the threshold indicating problematic balance (>0.20). Similarly, the weighted estimates yielded good distribution balance, with all covariates showing non-significant distributions differences. Given the low number of confirmed dropouts (n = 65), the balance indicators following the PA approach were highly similar (see Table A3).

Table A2

Balance Indicators After Fitting PSM Based on Intent To Treat (ITT) Approach

	Unweighted									Weighted								
	Treatment		Control							Treatment		Control						
Variable	M	SD	M	SD	SMD	t-test/ χ2	SMD *p*	KS	KS *p*	M	SD	M	SD	SMD	t-test/ χ2	SMD *p*	KS	KS *p*
Age	31.17	7.75	34.49	10.53	-0.43	-8.64	< .001	0.16	< .001	31.17	7.75	30.95	7.49	0.03	0.45	.655	0.04	.911
Ethnicity - Asian	0.07	0.26	0.08	0.28	-0.03	15.68	< .001	0.01	< .001	0.07	0.26	0.08	0.27	-0.01	1.46	.206	0.00	.206
Ethnicity – Black	0.22	0.41	0.11	0.32	0.25			0.10		0.22	0.41	0.15	0.36	0.15			0.06	
Ethnicity – Mixed	0.09	0.29	0.05	0.22	0.14			0.04		0.09	0.29	0.11	0.31	-0.05			0.01	
Ethnicity – White	0.59	0.49	0.73	0.44	-0.29			0.14		0.59	0.49	0.64	0.48	-0.10			0.05	
Ethnicity – Other	0.01	0.09	0.01	0.12	-0.06			0.01		0.01	0.09	0.01	0.12	-0.05			0.00	
Ethnicity - NA	0.02	0.13	0.00	0.06	0.10			0.01		0.02	0.13	0.00	0.06	0.10			0.01	
Prison – Yoi	0.06	0.23	0.13	0.34	-0.32	29.72	< .001	0.07	< .001	0.06	0.23	0.06	0.23	0.00	1.03	.386	0.00	.386
Prison – D-Cat	0.24	0.42	0.10	0.29	0.33			0.14		0.24	0.42	0.24	0.43	0.00			0.00	
Prison – C-Cat	0.42	0.49	0.54	0.50	-0.24			0.12		0.42	0.49	0.49	0.50	-0.13			0.06	
Prison – B-Cat	0.27	0.44	0.22	0.42	0.10			0.04		0.27	0.44	0.21	0.41	0.13			0.06	
Prison – A-Cat	0.02	0.13	0.01	0.10	0.06			0.01		0.02	0.13	0.01	0.12	0.03			0.00	
Copas	-0.98	0.78	-0.62	0.80	-0.47	-10.10	< .001	0.19	< .001	-0.98	0.78	-0.88	0.69	-0.13	-1.91	.056	0.09	.168
Copas - NA	0.05	0.22	0.07	0.26	-0.09	-1.95	.052	0.02	0.987	0.05	0.22	0.03	0.16	0.11	2.14	.032	0.03	1.000
IEP – Basic	0.01	0.09	0.04	0.20	-0.34	211.04	< .001	0.03	< .001	0.01	0.09	0.01	0.11	-0.04	2.65	.083	0.00	.083
IEP – Standard	0.21	0.41	0.71	0.46	-1.20			0.49		0.21	0.41	0.25	0.43	-0.09			0.04	
IEP – Enhanced	0.71	0.46	0.19	0.39	1.14			0.52		0.71	0.46	0.72	0.45	-0.03			0.01	
IEP – NA	0.07	0.25	0.06	0.24	0.02			0.00		0.07	0.25	0.01	0.12	0.22			0.06	
Adjudications (pre)	0.12	0.45	0.20	0.79	-0.17	-3.08	.002	0.03	0.911	0.12	0.45	0.15	0.58	-0.06	-0.96	.337	0.01	1.000

Table A2

Balance Indicators After Fitting PSM Based on Intent To Treat (ITT) Approach

	Unweighted									Weighted								
	Treatment		Control							Treatment		Control						
Variable	M	SD	M	SD	SMD	t-test/ $\chi2$	SMD *p*	KS	KS *p*	M	SD	M	SD	SMD	t-test/ $\chi2$	SMD *p*	KS	KS *p*
Casenote balance (pre)	0.66	2.27	-0.24	2.83	0.40	6.82	< .001	0.17	< .001	0.66	2.27	0.70	2.15	-0.02	-0.29	.771	0.05	.809
Casenote balance (pre) - NA	0.00		0.56	0.50		-48.91	< .001	0.56	< .001	0.00		0.00	0.02		-12.03	< .001	0.00	1.000
Self-harm (pre)	0.01	0.21	0.07	0.65	-0.27	-3.26	.001	0.02	0.935	0.01	0.21	0.02	0.28	-0.02	-0.54	.588	0.00	1.000
IO: Criminal damage and arson	0.00	0.07	0.02	0.13	-0.18	16.43	< .001	0.01	< .001	0.00	0.07	0.00	0.06	0.01	1.51	.128	0.00	.128
IO: Drug offences	0.32	0.47	0.20	0.40	0.27			0.13		0.32	0.47	0.33	0.47	-0.01			0.00	
IO: Fraud offences	0.01	0.11	0.01	0.09	0.03			0.00		0.01	0.11	0.01	0.11	-0.01			0.00	
IO: Miscellaneous crimes against society	0.04	0.18	0.05	0.21	-0.06			0.01		0.04	0.18	0.03	0.17	0.02			0.00	
IO: Possession of weapons	0.05	0.21	0.05	0.22	-0.03			0.01		0.05	0.21	0.04	0.20	0.02			0.00	
IO: Public order offences	0.02	0.14	0.07	0.26	-0.39			0.05		0.02	0.14	0.02	0.13	0.02			0.00	
IO: Robbery	0.12	0.32	0.08	0.27	0.13			0.04		0.12	0.32	0.12	0.33	-0.02			0.01	
IO: Sexual offences	0.01	0.10	0.06	0.24	-0.51			0.05		0.01	0.10	0.01	0.10	0.01			0.00	
IO: Summary motoring	0.01	0.08	0.01	0.11	-0.08			0.01		0.01	0.08	0.00	0.02	0.07			0.01	
IO: Summary non-motoring	0.01	0.09	0.03	0.16	-0.24			0.02		0.01	0.09	0.00	0.06	0.04			0.00	
IO: Theft offences	0.07	0.26	0.17	0.38	-0.39			0.10		0.07	0.26	0.09	0.28	-0.06			0.01	
IO: Unknown	0.00		0.00	0.05				0.00		0.00		0.00	0.03				0.00	
IO: Violence against the person	0.31	0.46	0.25	0.43	0.14			0.06		0.31	0.46	0.34	0.47	-0.06			0.03	
IO: <NA>	0.03	0.17	0.00		0.17			0.03		0.03	0.17	0.00		0.17			0.03	

Note. p tests are two-tailed

Table A3

Balance Indicators After Fitting PSM Based on PA

	Unweighted									Weighted								
	Treatment		Control							Treatment		Control						
Variable	M	SD	M	SD	SMD	t-test/ χ2	SMD *p*	KS	KS *p*	M	SD	M	SD	SMD	t-test/ χ2	SMD *p*	KS	KS *p*
Age	31.08	7.84	34.49	10.53	-0.44	-8.58	< .001	0.17	< .001	31.08	7.84	31.04	7.74	0.01	0.07	.940	0.03	.996
Ethnicity - Asian	0.08	0.27	0.08	0.28	-0.02	16.49	< .001	0.00	< .001	0.08	0.27	0.08	0.28	-0.01	1.60	.163	0.00	.163
Ethnicity – Black	0.22	0.42	0.11	0.32	0.27			0.11		0.22	0.42	0.16	0.36	0.16			0.07	
Ethnicity – Mixed	0.10	0.30	0.05	0.22	0.15			0.04		0.10	0.30	0.12	0.32	-0.07			0.02	
Ethnicity – White	0.58	0.49	0.73	0.44	-0.32			0.16		0.58	0.49	0.63	0.48	-0.10			0.05	
Ethnicity – Other	0.01	0.10	0.01	0.12	-0.05			0.00		0.01	0.10	0.01	0.12	-0.04			0.00	
Ethnicity - NA	0.01	0.12	0.00	0.06	0.09			0.01		0.01	0.12	0.00	0.05	0.10			0.01	
Prison – Yoi	0.06	0.24	0.13	0.34	-0.29	20.99	< .001	0.07	< .001	0.06	0.24	0.06	0.24	-0.01	0.72	.552	0.00	.552
Prison – D-Cat	0.20	0.40	0.10	0.29	0.27			0.11		0.20	0.40	0.20	0.40	0.00			0.00	
Prison – C-Cat	0.44	0.50	0.54	0.50	-0.21			0.10		0.44	0.50	0.49	0.50	-0.11			0.06	
Prison – B-Cat	0.28	0.45	0.22	0.42	0.12			0.05		0.28	0.45	0.22	0.41	0.13			0.06	
Prison – A-Cat	0.02	0.14	0.01	0.10	0.07			0.01		0.02	0.14	0.02	0.14	0.00			0.00	
Copas	-0.99	0.78	-0.62	0.80	-0.47	-9.87	< .001	0.19	< .001	-0.99	0.78	-0.89	0.70	-0.13	-1.83	.068	0.09	.202
Copas - NA	0.05	0.22	0.07	0.26	-0.09	-1.92	.055	0.02	.989	0.05	0.22	0.02	0.15	0.13	2.77	.006	0.03	.999
IEP – Basic	0.01	0.08	0.04	0.20	-0.43	197.09	< .001	0.03	< .001	0.01	0.08	0.01	0.10	-0.04	3.92	.023	0.00	.023
IEP – Standard	0.22	0.42	0.71	0.46	-1.17			0.49		0.22	0.42	0.26	0.44	-0.10			0.04	
IEP – Enhanced	0.70	0.46	0.19	0.39	1.12			0.51		0.70	0.46	0.72	0.45	-0.04			0.02	
IEP – NA	0.07	0.26	0.06	0.24	0.03			0.01		0.07	0.26	0.01	0.11	0.23			0.06	
Adjudications (pre)	0.13	0.45	0.20	0.79	-0.17	-2.91	.004	0.03	.897	0.13	0.45	0.15	0.59	-0.05	-0.76	.445	0.01	1.000

Table A3

Balance Indicators After Fitting PSM Based on PA

	Unweighted									Weighted								
	Treatment		Control							Treatment		Control						
Variable	M	SD	M	SD	SMD	t-test/ χ2	SMD *p*	KS	KS *p*	M	SD	M	SD	SMD	t-test/ χ2	SMD *p*	KS	KS *p*
Casenote balance (pre)	0.68	2.31	-0.24	2.83	0.40	6.80	< .001	0.19	< .001	0.68	2.31	0.69	2.25	0.00	-0.08	.936	0.05	.792
Casenote balance (pre) - NA	0.00		0.56	0.50		-48.91	< .001	0.56	< .001	0.00		0.00	0.06		-12.61	< .001	0.00	1.000
Self-harm (pre)	0.01	0.22	0.07	0.65	-0.25	-3.13	.002	0.02	.960	0.01	0.22	0.02	0.32	-0.04	-0.74	.459	0.00	1.000
IO: Criminal damage and arson	0.00	0.07	0.02	0.13	-0.17	15.10	< .001	0.01	< .001	0.00	0.07	0.00	0.06	0.01	1.72	.069	0.00	.069
IO: Drug offences	0.32	0.47	0.20	0.40	0.27			0.12		0.32	0.47	0.33	0.47	-0.01			0.00	
IO: Fraud offences	0.01	0.11	0.01	0.09	0.03			0.00		0.01	0.11	0.01	0.09	0.04			0.00	
IO: Miscellaneous crimes against society	0.03	0.18	0.05	0.21	-0.09			0.01		0.03	0.18	0.03	0.18	0.00			0.00	
IO: Possession of weapons	0.05	0.22	0.05	0.22	-0.02			0.00		0.05	0.22	0.04	0.20	0.03			0.01	
IO: Public order offences	0.02	0.14	0.07	0.26	-0.36			0.05		0.02	0.14	0.02	0.13	0.02			0.00	
IO: Robbery	0.11	0.32	0.08	0.27	0.11			0.03		0.11	0.32	0.12	0.32	-0.01			0.00	
IO: Sexual offences	0.01	0.11	0.06	0.24	-0.48			0.05		0.01	0.11	0.01	0.10	0.02			0.00	
IO: Summary motoring	0.01	0.08	0.01	0.11	-0.07			0.01		0.01	0.08	0.00	0.03	0.07			0.01	
IO: Summary non-motoring	0.01	0.09	0.03	0.16	-0.22			0.02		0.01	0.09	0.00	0.06	0.05			0.00	
IO: Theft offences	0.08	0.26	0.17	0.38	-0.37			0.10		0.08	0.26	0.09	0.28	-0.05			0.01	
IO: Unknown	0.00		0.00	0.05				0.00		0.00		0.00	0.03				0.00	
IO: Violence against the person	0.31	0.46	0.25	0.43	0.14			0.07		0.31	0.46	0.35	0.48	-0.07			0.03	
IO: <NA>	0.03	0.17	0.00		0.18			0.03		0.03	0.17	0.00		0.18			0.03	

Note. p tests are two-tailed

Treatment effects

We extract and apply weights to fit models predicting post-treatment prison behaviour based on treatment group membership (model 1) and additionally based on matching criteria to estimate doubly robust estimates (model 2) following both ITT and PA approaches.

Results show a significant treatment effect on adjudications (Table A4, Model 1). In the doubly robust model, we also find the significant treatment effect, and also significant effects of other prison behaviour and criminal history indicators. Individuals with more baseline adjudication and self-harm incidents received more adjudications after the programme. Individuals with higher Copas rate, and certain index offences, particularly violent or drug related offences, received more adjudications. Effect patterns were highly similar for the models following the PA approach.

We did not find significant treatment effects on post treatment case notes balance (Table A5), which was instead linked to pre-treatment case notes balance, and being classed in the most favourable IEP category (enhanced). We also did not find treatment effects on self-harm incidents (Table A6), which was significantly correlated with white ethnic ethnicity in the ITT model, or white ethnicity and pre-treatment self-harm incidents in the PA model.

Table A4

General Linear Model Predicting Adjudications Based on Treatment (Model 1) and Based on Treatment and Matching Parameters (Model 2) Following the Intent to Treat (ITT) and Protocol Adherence (PA) Approach

		ITT					PA				
Model	Effect	B	SE	t	*p*	95_CI	B	SE	t	*p*	95_CI
Model 1	(Intercept)	0.31	0.07	4.19	< .001	0.166 - 0.458	0.32	0.08	4.14	< .001	0.169 - 0.474
	TP Treatment	-0.16	0.08	-2.06	.039	-0.311 - -0.008	-0.18	0.08	-2.20	.028	-0.334 - -0.02
Model 2	(Intercept)	0.52	0.21	2.50	.012	0.111 - 0.92	0.48	0.23	2.12	.034	0.037 - 0.931
	TP Treatment	-0.15	0.08	-2.05	.040	-0.303 - -0.007	-0.18	0.08	-2.24	.025	-0.33 - -0.022
	Age	-0.01	0.01	-1.32	.188	-0.024 - 0.005	-0.01	0.01	-1.37	.170	-0.026 - 0.004
	Ethnicity – Black	0.21	0.09	2.40	.017	0.038 - 0.382	0.24	0.09	2.75	.006	0.069 - 0.415
	Ethnicity – Mixed	0.00	0.06	-0.06	.953	-0.127 - 0.12	0.01	0.06	0.22	.826	-0.109 - 0.136
	Ethnicity – White	0.11	0.07	1.48	.140	-0.036 - 0.257	0.12	0.08	1.64	.101	-0.024 - 0.271
	Ethnicity – Other	-0.08	0.09	-0.89	.374	-0.246 - 0.092	-0.07	0.08	-0.82	.414	-0.234 - 0.096
	Prison – D-Cat	-0.09	0.11	-0.78	.436	-0.3 - 0.129	-0.10	0.12	-0.85	.396	-0.325 - 0.129
	Prison – C-Cat	0.03	0.15	0.20	.844	-0.271 - 0.331	0.01	0.16	0.07	.947	-0.309 - 0.331
	Prison – B-Cat	-0.04	0.12	-0.32	.756	-0.273 - 0.197	-0.05	0.12	-0.40	.690	-0.293 - 0.194
	Prison – A-Cat	0.19	0.36	0.53	.594	-0.518 - 0.905	0.13	0.37	0.35	.728	-0.591 - 0.846
	Copas	0.07	0.03	2.17	.030	0.006 - 0.125	0.06	0.03	1.87	.061	-0.003 - 0.124
	IEP – Standard	-0.13	0.14	-0.91	.363	-0.411 - 0.15	-0.06	0.17	-0.34	.733	-0.382 - 0.269
	IEP – Enhanced	-0.20	0.15	-1.35	.178	-0.491 - 0.091	-0.12	0.17	-0.74	.460	-0.446 - 0.202

Table A4

General Linear Model Predicting Adjudications Based on Treatment (Model 1) and Based on Treatment and Matching Parameters (Model 2) Following the Intent to Treat (ITT) and Protocol Adherence (PA) Approach

		ITT					PA				
Model	Effect	B	SE	t	*p*	95_CI	B	SE	t	*p*	95_CI
	Adjudications (pre)	0.21	0.08	2.76	.006	0.061 - 0.36	0.19	0.06	2.98	.003	0.064 - 0.312
	Casenote balance (pre)	-0.01	0.01	-0.39	.696	-0.035 - 0.023	-0.01	0.02	-0.44	.662	-0.037 - 0.023
	Self-harm (pre)	0.29	0.13	2.13	.033	0.023 - 0.548	0.25	0.13	1.99	.047	0.003 - 0.502
	IO: Drug offences	0.19	0.06	3.38	.001	0.079 - 0.299	0.18	0.06	3.05	.002	0.064 - 0.294
	IO: Fraud offences	0.34	0.13	2.65	.008	0.088 - 0.594	0.37	0.15	2.52	.012	0.081 - 0.653
	IO: Miscellaneous crimes against society	0.16	0.09	1.86	.062	-0.008 - 0.327	0.16	0.10	1.63	.104	-0.033 - 0.353
	IO: Possession of weapons	0.15	0.10	1.49	.136	-0.046 - 0.339	0.15	0.10	1.46	.144	-0.051 - 0.348
	IO: Public order offences	0.27	0.14	1.97	.049	0.001 - 0.533	0.26	0.14	1.86	.064	-0.015 - 0.544
	IO: Robbery	0.20	0.09	2.29	.022	0.028 - 0.367	0.17	0.09	1.99	.046	0.003 - 0.342
	IO: Sexual offences	0.40	0.35	1.15	.251	-0.285 - 1.089	0.40	0.36	1.11	.267	-0.309 - 1.113
	IO: Summary motoring	0.79	0.37	2.13	.033	0.064 - 1.521	0.78	0.36	2.17	.030	0.075 - 1.483
	IO: Summary non-motoring	0.07	0.09	0.75	.456	-0.11 - 0.245	0.06	0.09	0.67	.506	-0.119 - 0.242
	IO: Theft offences	0.07	0.07	0.92	.360	-0.075 - 0.205	0.06	0.07	0.85	.397	-0.083 - 0.208
	IO: Unknown	0.01	0.10	0.06	.953	-0.187 - 0.198	-0.01	0.10	-0.14	.890	-0.22 - 0.191
	IO: Violence against the person	0.30	0.08	3.56	< .001	0.133 - 0.460	0.28	0.09	3.15	.002	0.107 - 0.46

Note. P tests are two-tailed. ITT Model 1, $F(1, 2548) = 4.26$, $p = .039$; ITT Model 2, $F(28, 1341) = 3.64$, $p < .001$; PA Model 1 $F(1, 2493) = 4.86$, $p = .028$; ITT Model 2, $F(28, 1291) = 3.46$, $p < .001$

Table A5

General Linear Model Predicting Case Note Balance Based on Treatment (Model 1) and Based on Treatment and Matching Parameters (Model 2) Following the Intent to Treat (ITT) and Protocol Adherence (PA) Approach

		ITT					PA				
Model	Effect	B	SE	t	*p*	95_CI	B	SE	t	*p*	95_CI
Model 1	(Intercept)	0.32	0.14	2.25	.024	0.041 - 0.59	0.28	0.14	1.97	.049	0.002 - 0.563
	TP Treatment	0.17	0.17	0.99	.323	-0.167 - 0.506	0.27	0.18	1.51	.132	-0.08 - 0.61
Model 2	(Intercept)	-2.67	1.08	-2.47	.014	-4.789 - -0.551	-1.69	0.97	-1.75	.080	-3.592 - 0.202
	TP Treatment	0.22	0.17	1.26	.207	-0.121 - 0.556	0.29	0.19	1.55	.121	-0.076 - 0.653
	Age	0.02	0.01	1.53	.125	-0.005 - 0.042	0.02	0.01	1.60	.110	-0.005 - 0.044
	Ethnicity – Black	0.19	0.30	0.62	.534	-0.399 - 0.769	0.04	0.30	0.13	.899	-0.56 - 0.637
	Ethnicity – Mixed	0.28	0.30	0.93	.352	-0.312 - 0.876	0.27	0.31	0.88	.377	-0.332 - 0.875
	Ethnicity – White	0.27	0.28	0.96	.336	-0.279 - 0.817	0.19	0.28	0.67	.501	-0.367 - 0.75
	Ethnicity – Other	0.51	0.60	0.84	.401	-0.677 - 1.689	0.37	0.62	0.60	.547	-0.84 - 1.583
	Prison – D-Cat	-0.12	0.49	-0.25	.800	-1.09 - 0.84	-0.07	0.51	-0.14	.886	-1.072 - 0.926
	Prison – C-Cat	-0.29	0.50	-0.58	.560	-1.282 - 0.694	-0.26	0.52	-0.50	.620	-1.275 - 0.761
	Prison – B-Cat	0.02	0.51	0.03	.976	-0.991 - 1.022	0.02	0.53	0.04	.966	-1.02 - 1.066
	Prison – A-Cat	1.62	1.09	1.49	.136	-0.512 - 3.748	1.25	1.20	1.04	.296	-1.098 - 3.601
	Copas	-0.20	0.12	-1.60	.111	-0.437 - 0.045	-0.19	0.13	-1.45	.148	-0.446 - 0.068
	IEP – Standard	1.50	0.89	1.69	.091	-0.241 - 3.251	0.49	0.73	0.66	.508	-0.953 - 1.924

Table A5

General Linear Model Predicting Case Note Balance Based on Treatment (Model 1) and Based on Treatment and Matching Parameters (Model 2) Following the Intent to Treat (ITT) and Protocol Adherence (PA) Approach

		ITT					PA				
Model	Effect	B	SE	t	*p*	95_CI	B	SE	t	*p*	95_CI
	IEP – Enhanced	1.94	0.89	2.17	.030	0.19 - 3.697	0.91	0.74	1.24	.216	-0.533 - 2.352
	Adjudications (pre)	-0.17	0.17	-0.98	.329	-0.502 - 0.168	-0.20	0.17	-1.20	.231	-0.539 - 0.13
	Casenote balance (pre)	0.27	0.06	4.70	< .001	0.156 - 0.379	0.27	0.06	4.62	< .001	0.156 - 0.387
	Self-harm (pre)	-0.13	0.11	-1.13	.257	-0.344 - 0.092	-0.15	0.11	-1.32	.188	-0.362 - 0.071
	IO: Drug offences	0.22	0.29	0.76	.449	-0.349 - 0.787	0.29	0.29	0.98	.329	-0.29 - 0.863
	IO: Fraud offences	1.01	0.62	1.64	.100	-0.195 - 2.218	1.03	0.72	1.44	.149	-0.372 - 2.44
	IO: Miscellaneous crimes against society	-0.05	0.43	-0.12	.904	-0.899 - 0.795	-0.02	0.46	-0.03	.974	-0.921 - 0.89
	IO: Possession of weapons	-0.04	0.43	-0.08	.933	-0.88 - 0.808	-0.10	0.44	-0.23	.816	-0.97 - 0.764
	IO: Public order offences	-0.20	0.39	-0.53	.596	-0.962 - 0.553	-0.18	0.39	-0.47	.638	-0.948 - 0.581
	IO: Robbery	0.05	0.37	0.13	.895	-0.674 - 0.772	0.14	0.39	0.36	.717	-0.622 - 0.903
	IO: Sexual offences	0.37	0.64	0.58	.565	-0.882 - 1.614	0.36	0.65	0.56	.579	-0.911 - 1.63
	IO: Summary motoring	1.07	0.77	1.40	.163	-0.434 - 2.574	1.03	0.73	1.41	.158	-0.401 - 2.471
	IO: Summary non-motoring	0.08	0.44	0.19	.852	-0.777 - 0.941	0.10	0.46	0.22	.826	-0.801 - 1.003
	IO: Theft offences	0.04	0.40	0.10	.918	-0.738 - 0.819	0.12	0.41	0.29	.773	-0.691 - 0.929
	IO: Unknown	0.13	0.34	0.37	.709	-0.546 - 0.803	0.20	0.35	0.56	.577	-0.492 - 0.883
	IO: Violence against the person	0.08	0.30	0.28	.777	-0.496 - 0.664	0.07	0.30	0.25	.804	-0.519 - 0.669

Note. ITT Model 1, $F(1, 1497) = 0.98$, $p = .323$; ITT Model 2, $F(28, 1341) = 4.38$, $p < .001$; PA Model 1, PA Model 1, $F(1, 1442) = 2.27$, $p = .132$; ITT Model 2, $F(28, 1291) = 4.11$, $p < .001$.

Table A6

General Linear Model Predicting Self-Harm Incidents Based on Treatment (Model 1) and Based on Treatment and Matching Parameters (Model 2) Following the Intent to Treat (ITT) and Protocol Adherence (PA) Approach

		ITT					PA				
Model	Effect	B	SE	t	*p*	95_CI	B	SE	t	*p*	95_CI
Model 1	(Intercept)	0.04	0.01	3.43	.001	0.018 - 0.067	0.04	0.01	3.37	.001	0.018 - 0.069
	TP Treatment	-0.02	0.02	-1.37	.170	-0.053 - 0.009	-0.02	0.02	-1.34	.179	-0.056 - 0.01
Model 2	(Intercept)	0.03	0.09	0.33	.738	-0.139 - 0.196	0.02	0.09	0.16	.869	-0.167 - 0.197
	TP Treatment	-0.01	0.02	-0.56	.576	-0.044 - 0.025	-0.01	0.02	-0.58	.563	-0.048 - 0.026
	Age	0.00	0.00	-1.64	.101	-0.005 – 0.000	0.00	0.00	-1.70	.090	-0.005 – 0.000
	Ethnicity – Black	0.01	0.01	1.32	.187	-0.007 - 0.036	0.02	0.01	1.44	.150	-0.006 - 0.037
	Ethnicity – Mixed	0.01	0.01	0.88	.381	-0.012 - 0.033	0.01	0.01	0.88	.378	-0.013 - 0.035
	Ethnicity – White	0.05	0.02	2.81	.005	0.015 - 0.086	0.05	0.02	2.89	.004	0.017 - 0.088
	Ethnicity – Other	0.01	0.03	0.42	.672	-0.044 - 0.068	0.01	0.03	0.46	.644	-0.041 - 0.067
	Prison – D-Cat	0.01	0.03	0.36	.718	-0.044 - 0.064	0.01	0.03	0.32	.747	-0.054 - 0.075
	Prison – C-Cat	0.03	0.03	0.90	.366	-0.036 - 0.098	0.03	0.04	0.70	.485	-0.049 - 0.102
	Prison – B-Cat	0.02	0.03	0.75	.456	-0.04 - 0.089	0.02	0.04	0.66	.512	-0.049 - 0.098
	Prison – A-Cat	0.01	0.04	0.19	.849	-0.062 - 0.076	0.01	0.04	0.30	.767	-0.067 - 0.090
	Copas	0.02	0.02	1.20	.230	-0.013 - 0.055	0.02	0.02	1.21	.225	-0.014 - 0.058
	IEP – Standard	0.01	0.07	0.15	.879	-0.13 - 0.151	0.03	0.08	0.33	.743	-0.127 - 0.178

Table A6

General Linear Model Predicting Self-Harm Incidents Based on Treatment (Model 1) and Based on Treatment and Matching Parameters (Model 2) Following the Intent to Treat (ITT) and Protocol Adherence (PA) Approach

		ITT					PA				
Model	Effect	B	SE	t	*p*	95_CI	B	SE	t	*p*	95_CI
	IEP – Enhanced	-0.03	0.07	-0.39	.695	-0.154 - 0.103	-0.01	0.07	-0.14	.892	-0.149 - 0.13
	Adjudications (pre)	0.07	0.06	1.14	.256	-0.052 - 0.197	0.06	0.07	0.91	.364	-0.071 - 0.192
	Casenote balance (pre)	0.00	0.01	0.73	.465	-0.007 - 0.016	0.00	0.01	0.67	.503	-0.008 - 0.017
	Self-harm (pre)	0.17	0.09	1.86	.063	-0.009 - 0.353	0.16	0.08	2.02	.043	0.005 - 0.312
	IO: Drug offences	0.03	0.02	1.11	.265	-0.021 - 0.075	0.03	0.02	1.55	.121	-0.009 - 0.075
	IO: Fraud offences	0.04	0.03	1.45	.147	-0.015 - 0.103	0.04	0.03	1.62	.105	-0.009 - 0.099
	IO: Miscellaneous crimes against society	0.18	0.15	1.21	.228	-0.111 - 0.465	0.20	0.16	1.25	.213	-0.115 - 0.518
	IO: Possession of weapons	0.01	0.02	0.24	.811	-0.041 - 0.052	0.01	0.02	0.62	.535	-0.027 - 0.053
	IO: Public order offences	0.00	0.03	0.06	.952	-0.05 - 0.053	0.01	0.02	0.33	.744	-0.039 - 0.054
	IO: Robbery	0.05	0.04	1.18	.238	-0.034 - 0.137	0.06	0.05	1.39	.166	-0.026 - 0.153
	IO: Sexual offences	0.03	0.03	0.93	.351	-0.035 - 0.1	0.05	0.03	1.40	.163	-0.018 - 0.109
	IO: Summary motoring	0.03	0.03	0.94	.349	-0.031 - 0.086	0.03	0.03	1.05	.295	-0.025 - 0.081
	IO: Summary non-motoring	0.01	0.03	0.44	.658	-0.04 - 0.064	0.02	0.02	0.79	.428	-0.028 - 0.066
	IO: Theft offences	-0.01	0.03	-0.23	.819	-0.06 - 0.048	0.00	0.02	0.01	.991	-0.048 - 0.049
	IO: Unknown	0.00	0.04	0.03	.977	-0.068 - 0.07	0.00	0.04	0.01	.994	-0.069 - 0.069
	IO: Violence against the person	0.05	0.03	1.78	.076	-0.005 - 0.096	0.05	0.02	2.19	.029	0.005 - 0.097

Note. P tests are two-tailed. ITT Model 1, $F(1, 2548) = 1.89$, $p = .170$; ITT Model 2, $F(28, 1341) = 1.11$, $p = .313$; PA Model 1, $F(1, 2493) = 1.80$, $p = .180$; ITT Model 2, $F(28, 1291) = 1.17$, $p = .248$

Sensitivity analyses

To test the robustness of our finding that participation in Twinning Project reduces the number of adjudications, we conducted sensitivity analyses targeting various aspects of the design of the main reported model. Specifically, we altered the following model parameters and analyses techniques and report here how they impact case/control group balance and treatment effects.

1) **Covariate coding**: We tested alternative coding schemes for continuous and categorical variables, i.e., coding IEP-levels and prison security categories as continuous (rather than ordinal variables), coding age as ordinal (rather than continuous), coding ethnicity as a binary indicator of ethnic minority status (rather than a multicategorical nominal variable).

2) **Model type used to estimate treatment effects**: Estimates can be sensitive to model structure, especially when treatment and control cases are unlike (as demonstrated in this case by the unweighted balance comparison in Tables A1 & A2). We test the outcomes using a linear model function instead of the generalised linear model function.

3) **Method to calculate the propensity score**: The quality of balance and treatment effects can be sensitive to the method used to estimate propensity scores. We tested logistic regression models to calculate propensity scores and extract weights for treatment models.

4) **Method to calculate control group "observation/"treatment" period**: We tested alternative approaches to assign the observation period , i.e., "treatment" start time for the control cases, using a) the median (instead of the mean) treatment-to-release time of the experimental group, and b) assigning a random date within the research period to each control case , repeated over 1.000 iterations using a bootstrapping procedure.

Overall, the main finding of a treatment effect on adjudications was not sensitive to the vast majority of tests (Table A7), indicating that our main model is robust. Balance and treatment effects from the alternative models adjusting covariate coding, treatment or propensity score models were highly similar to the main model. Adjusting the method to define and construct the observation period of the control group posed a challenge, due to the fact that cases in the control sample had significantly more time left to serve.

The eligible control population (i.e., male & in public prisons; n = 4334) had on average 32 months left to serve, compared to the 19 months of the treatment population. The approach for the main model was to fix the control group observation period to 19 months from release, which reduced the available control sample to n = 1874, effectively utilising n = 223 cases (12%). Drawing on a control group constructed based on the median 'time until release' further reduced the available sample to n = 1241, but using 'time until release' as a matching parameter lead to substantial imbalance on multiple variables. Constructing the control sample based on randomised observation periods allowed us to draw on all n = 4334 cases, and a larger effective sample size (n = 356), but the relative effective sample size was identical to the main analysis (12%) and it was not possible to reduce the standardised mean difference of 'time until release' below the desired SMD <.20 and KS < .10. We provide full model results of the sensitivity analyses in the separate Supplementary Information J.

Table A7

Overview of Sensitivity Analyses

Model	Control Observation period	PS method	Treatment effect model	Covariate coding	Control sample		Balance after weighting		Treatment effects (ITT)		Treatment effect (PA)	
					N	Effective N	Mean SMD	Mean KS	Simple	Doubly robust	Simple	Doubly robust
Main	Mean	GBM	GLM	STD	1874	223.65	0.06	0.02	-.16 (.08) *p* = .09	-.15 (.08) *p* = .040	-.18 (.08) *p* = .028	-.18 (.18) *p* = .034
Alt 1	Mean	GBM	GLM	ALT	1874	239.15	0.06	0.02	-.15 (.07) *p* = .028	-.13 (.07) *p* = .048	-.15 (.06) *p* = .013	-.13 (.06) *p* = .014
Alt 2	Mean	GBM	LM	STD	-	-	-	-	-.16 (.03) *p* < .001	-.15 (.04) p<.001	-.18 (.03) p<.001	-.18 (.04) p<.001
Alt 3	Mean	Logit	GLM	STD	764	191.73	0.04	0.02	-.17 (.08) *p* = .029	-.16 (.07) *p* = .018	-.18 (.07) *p* = .018	-.18 (.07) *p* = .010
Alt 4.1	Median	GBM	GLM	STD	1241	151.16	0.21[a]	0.09	-	-	-	-
Alt 4.2	Median	GBM	GLM	STD	1241	175.25	0.08	0.02	-.23 (.08) *p* = .006	-.21 (.09) *p* = .014	-.26 (.09) *p* = .005	-.25 (.09) *p* = .009
Alt 5	Random	GBM	GLM	STD	4334	356.98	0.08[b]	0.03	-.09 (.04) *p* = .027 [.85]	-.08 (.04) *p* = .163 [.33]	-.10 (.04) *p* = .017 [.92]	-.09 (.04) *p* = .097 [.48]

Note. Overview of alternative modelling approaches in comparison to the main model (main) presented in the manuscript. For each alternative (alt.) approach, we specify: 1) the observation period for the control group (based on mean, median or random sampling), 2) the propensity score (PS) method, either gradient boosted regression (GBM) or logistic regression (Logit), 3) the treatment effect model, either generalised linear model (GLM) or linear model (LM), and 4) the covariate coding following either the standard coding (STD) or an alternative coding (ALT) as outlined above.

Statistics for control sample size and balance after weighting are based on the ITT approach. Balance indicators are presented for PSM following ITT approach.

Treatment effects are unstandardised estimates with standard errors in parentheses.

Model Alt 1 full results can be found in Tables A8 - A11.

Model Alt 2 full results can be found in Tables A12.

Model Alt 3 full results can be found in Tables A13 – A14.

Model Alt 4.1 included ‘time until release’ as matching parameter for the PSM. [a] Individual covariates statistically significant ($p < .05$) **and** beyond the cutoff points for respective metrics (SMD > .20 or KS >.10): ethnicity, prison type, IEP-level, Copas, adjudications, self-harm, index offence.

Model Alt 4.2 included ‘time until release’ as covariate in treatment effect model.

Full results for Alt 4.1 and Alt 4.2 can be found in Tables A15 – A18.

Model Alt 5 Estimates and *p* values are averages of 1.000 Bootstrapp iterations. In addition to average unstandardised estimates, standard errors and *p* values we provide the percentage of iterations with $p < .05$ in square brackets [ ] on a scale from 0 to 1.

[b] Individual covariates statistically significant ($p < .05$) **and** beyond the cutoff points for respective metrics (SMD > .20 or KS >.10): time until release. Full results for Model Alt 5 can be found in Tables A19 – A22.

Covariate coding

Using alternative covariate coding schemes yielded highly similar results to the main balance analysis, both with regard to the summary statistics and individual covariates. Thus, the weights extracted via PSM improved balance compared unweighted estimates, both for the ITT and PA approach (Table A8). Besides differences in missing values for pre-treatment IEP-levels, no covariates showed standardised mean difference or KS differences above the respective critical thresholds of SMD > .20 and KS > .10 (see tables A9 & A10). Likewise, the general linear models showed the same significant treatment effect patterns, which held after controlling for matching parameters for both analyses following the ITT and PA approach (see Table A11).

Table A8

Summary of Balance Indicators Using Alternative Covariate Coding Schemes

	Treatment N	Control N	Control Effective N	Max. SMD	Mean SMD	Max. KS	Mean KS	Iterations
ITT								
Unw.	676	1874	1874	1.30	0.24	0.56	0.09	
Weigh.	676	1874	239.15	0.22	0.06	0.08	0.02	2079
PA								
Unw.	621	1874	1874	1.30	0.24	0.56	0.09	
Weigh.	621	1874	219.64	0.23	0.06	0.08	0.02	2010

Note. Results of group balance testing based on mean standardised bias stopping rule for the intent to treat approach and protocol adherence approach.

ITT = Intent to Treat approach, PA = Protocol Adherence approach, Unw = un-weighted, Weigh. = weighted, SMD = standardised mean difference, KS = Kolmogorov Smirnov statistic.

Table A9

Balance Indicators After Fitting PSM Based on Intent to Treat (ITT) Approach Using Alternative Covariate Coding Scheme

	Unweighted									Weighted								
	Treatment		Control							Treatment		Control						
Variable	M	SD	M	SD	SMD	t-test/ χ2	SMD *p*	KS	KS *p*	M	SD	M	SD	SMD	t-test/ χ2	SMD *p*	KS	KS *p*
Age group: 18-25	0.25	0.43	0.20	0.40	0.11	15.40	< .001	0.05	< .001	0.25	0.43	0.27	0.44	-0.05	0.42	.767	0.02	.767
Age group: 26-29	0.19	0.39	0.16	0.36	0.09			0.04		0.19	0.39	0.18	0.38	0.03			0.01	
Age group: 30-39	0.42	0.49	0.36	0.48	0.13			0.06		0.42	0.49	0.43	0.50	-0.01			0.01	
Age group: 40-49	0.11	0.31	0.19	0.39	-0.26			0.08		0.11	0.31	0.09	0.28	0.07			0.02	
Age group: 50 or older	0.03	0.16	0.09	0.29	-0.42			0.06		0.03	0.16	0.03	0.17	-0.02			0.00	
Ethnic minority: No	0.59	0.49	0.73	0.44	-0.29	27.26	< .001	0.14	< .001	0.59	0.49	0.65	0.48	-0.11	3.48	.039	0.05	.039
Ethnic minority: Yes	0.39	0.49	0.26	0.44	0.26			0.13		0.39	0.49	0.35	0.48	0.08			0.04	
Ethnic minority: NA	0.02	0.13	0.00	0.06	0.10			0.01		0.02	0.13	0.00	0.05	0.11			0.01	
Prison type	2.95	0.89	2.89	0.93	0.07	1.58	.113	0.07	.009	2.95	0.89	2.90	0.85	0.06	0.89	.373	0.06	.559
Copas	-0.98	0.78	-0.62	0.80	-0.47	-10.10	< .001	0.19	< .001	-0.98	0.78	-0.89	0.69	-0.12	-1.80	.072	0.08	.184
Copas - NA	0.05	0.22	0.07	0.26	-0.09	-1.95	.052	0.02	.987	0.05	0.22	0.02	0.15	0.13	2.97	.003	0.03	.998
IEP	2.75	0.46	2.16	0.47	1.30	27.79	< .001	0.56	< .001	2.75	0.46	2.72	0.48	0.07	1.08	.281	0.03	1.000
IEP – NA	0.07	0.25	0.06	0.24	0.02	0.49	.627	0.00	1.000	0.07	0.25	0.01	0.12	0.22	3.43	.001	0.06	.638
Adjudications (pre)	0.12	0.45	0.20	0.79	-0.17	-3.08	.002	0.03	.911	0.12	0.45	0.15	0.57	-0.06	-1.00	.315	0.01	1.000
Casenote balance (pre)	0.66	2.27	-0.24	2.83	0.40	6.82	< .001	0.17	< .001	0.66	2.27	0.69	2.24	-0.01	-0.23	.820	0.05	.806
Casenote balance (pre) - NA	0.00		0.56	0.50		-48.91	< .001	0.56	< .001	0.00		0.00	0.03		-12.80	< .001	0.00	1.000
Self-harm (pre)	0.01	0.21	0.07	0.65	-0.27	-3.26	.001	0.02	.935	0.01	0.21	0.02	0.31	-0.02	-0.52	.601	0.00	1.000
IO: Criminal damage and arson	0.00	0.07	0.02	0.13	-0.18	16.43	< .001	0.01	< .001	0.00	0.07	0.00	0.06	0.01	1.87	.044	0.00	.044

Table A9

Balance Indicators After Fitting PSM Based on Intent to Treat (ITT) Approach Using Alternative Covariate Coding Scheme

	Unweighted									Weighted								
	Treatment		Control							Treatment		Control						
Variable	M	SD	M	SD	SMD	t-test/ $\chi2$	SMD *p*	KS	KS *p*	M	SD	M	SD	SMD	t-test/ $\chi2$	SMD *p*	KS	KS *p*
IO: Drug offences	0.32	0.47	0.20	0.40	0.27			0.13		0.32	0.47	0.33	0.47	0.00			0.00	
IO: Fraud offences	0.01	0.11	0.01	0.09	0.03			0.00		0.01	0.11	0.01	0.10	0.02			0.00	
IO: Miscellaneous crimes against society	0.04	0.18	0.05	0.21	-0.06			0.01		0.04	0.18	0.03	0.17	0.03			0.00	
IO: Possession of weapons	0.05	0.21	0.05	0.22	-0.03			0.01		0.05	0.21	0.04	0.20	0.03			0.01	
IO: Public order offences	0.02	0.14	0.07	0.26	-0.39			0.05		0.02	0.14	0.01	0.12	0.03			0.00	
IO: Robbery	0.12	0.32	0.08	0.27	0.13			0.04		0.12	0.32	0.12	0.32	0.00			0.00	
IO: Sexual offences	0.01	0.10	0.06	0.24	-0.51			0.05		0.01	0.10	0.01	0.10	0.01			0.00	
IO: Summary motoring	0.01	0.08	0.01	0.11	-0.08			0.01		0.01	0.08	0.00	0.02	0.07			0.01	
IO: Summary non-motoring	0.01	0.09	0.03	0.16	-0.24			0.02		0.01	0.09	0.00	0.05	0.05			0.00	
IO: Theft offences	0.07	0.26	0.17	0.38	-0.39			0.10		0.07	0.26	0.08	0.28	-0.04			0.01	
IO: Unknown	0.00		0.00	0.05				0.00		0.00		0.00	0.03				0.00	
IO: Violence against the person	0.31	0.46	0.25	0.43	0.14			0.06		0.31	0.46	0.36	0.48	-0.10			0.05	
IO: <NA>	0.03	0.17	0.00		0.17			0.03		0.03	0.17	0.00		0.17			0.03	

Table A10

Balance Indicators After Fitting PSM Based on Protocol Adherence (PA) Approach Using Alternative Covariate Coding Scheme

	Unweighted									Weighted								
	Treatment		Control							Treatment		Control						
Variable	M	SD	M	SD	SMD	t-test/ $\chi 2$	SMD *p*	KS	KS *p*	M	SD	M	SD	SMD	t-test/ $\chi 2$	SMD *p*	KS	KS *p*
Age group: 18-25	0.26	0.44	0.20	0.40	0.12	14.42	< .001	0.05	< .001	0.26	0.44	0.28	0.45	-0.05	0.47	.732	0.02	.732
Age group: 26-29	0.19	0.40	0.16	0.36	0.10			0.04		0.19	0.40	0.17	0.38	0.05			0.02	
Age group: 30-39	0.42	0.49	0.36	0.48	0.11			0.06		0.42	0.49	0.43	0.50	-0.02			0.01	
Age group: 40-49	0.11	0.31	0.19	0.39	-0.27			0.08		0.11	0.31	0.09	0.28	0.07			0.02	
Age group: 50 or older	0.03	0.16	0.09	0.29	-0.38			0.06		0.03	0.16	0.03	0.17	-0.02			0.00	
Ethnic minority: No	0.58	0.49	0.73	0.44	-0.32	29.63	< .001	0.16	< .001	0.58	0.49	0.63	0.48	-0.10	2.90	.067	0.05	.067
Ethnic minority: Yes	0.41	0.49	0.26	0.44	0.30			0.14		0.41	0.49	0.37	0.48	0.08			0.04	
Ethnic minority: NA	0.01	0.12	0.00	0.06	0.09			0.01		0.01	0.12	0.00	0.05	0.10			0.01	
Prison type	2.99	0.90	2.89	0.93	0.11	2.43	.015	0.07	.025	2.99	0.90	2.93	0.88	0.07	0.84	.398	0.05	.863
Copas	-0.99	0.78	-0.62	0.80	-0.47	-9.87	< .001	0.19	< .001	-0.99	0.78	-0.89	0.70	-0.12	-1.63	.102	0.08	.231
Copas - NA	0.05	0.22	0.07	0.26	-0.09	-1.92	.055	0.02	.989	0.05	0.22	0.02	0.14	0.14	2.95	.003	0.03	.998
IEP	2.75	0.45	2.16	0.47	1.31	27.01	< .001	0.55	< .001	2.75	0.45	2.72	0.47	0.06	0.98	.325	0.03	1.000
IEP – NA	0.07	0.26	0.06	0.24	0.03	0.71	.477	0.01	1.000	0.07	0.26	0.01	0.11	0.23	3.96	< .001	0.06	.586
Adjudications (pre)	0.13	0.45	0.20	0.79	-0.17	-2.91	.004	0.03	.897	0.13	0.45	0.14	0.57	-0.04	-0.67	.500	0.01	1.000
Casenote balance (pre)	0.68	2.31	-0.24	2.83	0.40	6.80	< .001	0.19	< .001	0.68	2.31	0.71	2.34	-0.01	-0.22	.827	0.05	.850
Casenote balance (pre) - NA	0.00		0.56	0.50		-48.91	< .001	0.56	< .001	0.00		0.00	0.03		-12.06	< .001	0.00	1.000

Table A10

Balance Indicators After Fitting PSM Based on Protocol Adherence (PA) Approach Using Alternative Covariate Coding Scheme

	Unweighted									Weighted								
	Treatment		Control							Treatment		Control						
Variable	M	SD	M	SD	SMD	t-test/ χ2	SMD *p*	KS	KS *p*	M	SD	M	SD	SMD	t-test/ χ2	SMD *p*	KS	KS *p*
Self-harm (pre)	0.01	0.22	0.07	0.65	-0.25	-3.13	.002	0.02	.960	0.01	0.22	0.02	0.32	-0.03	-0.54	.587	0.00	1.000
IO: Criminal damage and arson	0.00	0.07	0.02	0.13	-0.17	15.10	< .001	0.01	< .001	0.00	0.07	0.00	0.06	0.02	1.95	.035	0.00	.035
IO: Drug offences	0.32	0.47	0.20	0.40	0.27			0.12		0.32	0.47	0.32	0.47	0.00			0.00	
IO: Fraud offences	0.01	0.11	0.01	0.09	0.03			0.00		0.01	0.11	0.01	0.09	0.03			0.00	
IO: Miscellaneous crimes against society	0.03	0.18	0.05	0.21	-0.09			0.01		0.03	0.18	0.03	0.17	0.00			0.00	
IO: Possession of weapons	0.05	0.22	0.05	0.22	-0.02			0.00		0.05	0.22	0.04	0.20	0.03			0.01	
IO: Public order offences	0.02	0.14	0.07	0.26	-0.36			0.05		0.02	0.14	0.02	0.13	0.03			0.00	
IO: Robbery	0.11	0.32	0.08	0.27	0.11			0.03		0.11	0.32	0.11	0.32	0.00			0.00	
IO: Sexual offences	0.01	0.11	0.06	0.24	-0.48			0.05		0.01	0.11	0.01	0.10	0.01			0.00	
IO: Summary motoring	0.01	0.08	0.01	0.11	-0.07			0.01		0.01	0.08	0.00	0.02	0.07			0.01	
IO: Summary non-motoring	0.01	0.09	0.03	0.16	-0.22			0.02		0.01	0.09	0.00	0.05	0.06			0.00	
IO: Theft offences	0.08	0.26	0.17	0.38	-0.37			0.10		0.08	0.26	0.09	0.28	-0.04			0.01	
IO: Unknown	0.00		0.00	0.05				0.00		0.00		0.00	0.03				0.00	
IO: Violence against the person	0.31	0.46	0.25	0.43	0.14			0.07		0.31	0.46	0.37	0.48	-0.11			0.05	
IO: <NA>	0.03	0.17	0.00		0.18			0.03		0.03	0.17	0.00		0.18			0.03	

Table A11

General Linear Model Predicting Adjudications Based on Treatment (Model 1) And Based on Treatment and Matching Parameters (Model 2) Following the Intent to Treat (ITT) and Protocol Adherence (PA) Approach

		ITT					PA				
Model	Effect	B	SE	t	*p*	95_CI	B	SE	t	*p*	95_CI
Model 1	(Intercept)	0.30	0.07	4.64	< .001	0.175 - 0.43	0.29	0.06	5.24	< .001	0.182 - 0.401
	TP treatment	-0.15	0.07	-2.20	.028	-0.284 - -0.016	-0.15	0.06	-2.48	.013	-0.263 - -0.031
Model 2	(Intercept)	0.47	0.13	3.62	< .001	0.216 - 0.727	0.49	0.13	3.65	< .001	0.226 - 0.752
	TP treatment	-0.13	0.07	-1.98	.048	-0.261 - -0.001	-0.13	0.06	-2.26	.024	-0.244 - -0.017
	Age group: 26-29	-0.14	0.11	-1.27	.203	-0.367 - 0.078	-0.13	0.10	-1.32	.186	-0.318 - 0.062
	Age group: 30-39	-0.13	0.12	-1.07	.284	-0.356 - 0.105	-0.12	0.10	-1.26	.209	-0.307 - 0.067
	Age group: 40-49	-0.16	0.11	-1.40	.162	-0.383 - 0.064	-0.13	0.10	-1.34	.181	-0.317 - 0.06
	Age group: 50 or older	-0.24	0.12	-1.96	.051	-0.477 - 0.001	-0.22	0.10	-2.24	.026	-0.409 - -0.027
	Ethnic minority: Yes	0.01	0.06	0.20	.838	-0.1 - 0.123	0.03	0.05	0.66	.509	-0.066 - 0.133
	Prison type	0.01	0.03	0.32	.749	-0.049 - 0.068	0.00	0.03	-0.15	.884	-0.058 - 0.05
	Copas	0.07	0.03	2.26	.024	0.009 - 0.133	0.07	0.03	2.18	.030	0.007 - 0.131
	IEP	-0.10	0.05	-2.01	.044	-0.203 - -0.003	-0.09	0.05	-2.03	.042	-0.186 - -0.003
	Adjudications (pre)	0.22	0.07	2.89	.004	0.069 - 0.362	0.19	0.07	2.96	.003	0.065 - 0.321
	Casenote balance (pre)	-0.01	0.01	-1.08	.281	-0.039 - 0.011	-0.02	0.01	-1.32	.188	-0.04 - 0.008
	Self-harm (pre)	0.25	0.14	1.80	.072	-0.022 - 0.513	0.24	0.13	1.86	.063	-0.013 - 0.503

Table A11

General Linear Model Predicting Adjudications Based on Treatment (Model 1) And Based on Treatment and Matching Parameters (Model 2) Following the Intent to Treat (ITT) and Protocol Adherence (PA) Approach

		ITT					PA				
Model	Effect	B	SE	t	*p*	95_CI	B	SE	t	*p*	95_CI
	IO: Drug offences	0.18	0.05	3.71	< .001	0.085 - 0.275	0.16	0.05	3.32	.001	0.067 - 0.26
	IO: Fraud offences	0.27	0.13	2.07	.038	0.015 - 0.534	0.26	0.14	1.89	.058	-0.009 - 0.537
	IO: Miscellaneous crimes against society	0.17	0.08	2.11	.035	0.013 - 0.335	0.16	0.09	1.75	.081	-0.019 - 0.338
	IO: Possession of weapons	0.15	0.09	1.68	.093	-0.024 - 0.314	0.14	0.09	1.55	.122	-0.037 - 0.314
	IO: Public order offences	0.23	0.13	1.76	.079	-0.027 - 0.481	0.22	0.13	1.70	.089	-0.033 - 0.472
	IO: Robbery	0.24	0.07	3.34	.001	0.101 - 0.388	0.22	0.07	3.04	.002	0.077 - 0.358
	IO: Sexual offences	0.36	0.32	1.11	.269	-0.277 - 0.991	0.34	0.33	1.03	.302	-0.305 - 0.983
	IO: Summary motoring	0.78	0.41	1.87	.061	-0.037 - 1.589	0.75	0.41	1.82	.068	-0.057 - 1.555
	IO: Summary non-motoring	0.11	0.07	1.64	.100	-0.021 - 0.241	0.10	0.07	1.52	.128	-0.03 - 0.236
	IO: Theft offences	0.09	0.06	1.46	.144	-0.032 - 0.222	0.09	0.06	1.37	.170	-0.037 - 0.21
	IO: Unknown	-0.05	0.09	-0.55	.579	-0.219 - 0.123	-0.08	0.08	-0.90	.366	-0.241 - 0.089
	IO: Violence against the person	0.28	0.07	4.06	< .001	0.147 - 0.423	0.24	0.06	3.86	< .001	0.12 - 0.369

Note. P tests are two-tailed. ITT Model 1, $F(1, 2548) = 4.82$, $p = .028$; ITT Model 2, , $F(24, 1345) = 4.72$, $p < .001$; PA Model 1, $F(1, 2493) = 6.17$, $p = .013$; ITT Model 2, $F(24, 1295) = 4.47$, $p < .001$.

Model type used to estimate treatment effects

The alternative linear model function resulted in highly similar result patterns, with some differences regarding standard errors and significance levels compared to the GLM model version. Specifically, while estimates remained unchanged, standard errors were smaller for the treatment variable and age, whereas they were larger for the levels of the index offence variable, and remained the same for prison behaviour, background indicators and Copas rate. Thus, compared to the main model, significance levels of some variables were more pronounced, i.e., the effect of age became statistically significant, whereas the effects of index offences were no longer statistically significant. Importantly, treatment effects remained for both the simple and doubly robust models following both the ITT and PA approach (Table A12).

Table A12

Linear Models Predicting Adjudications Based on Treatment (Model 1) And Based on Treatment and Matching Parameters (Model 2) Following the Intent to Treat (ITT) and Protocol Adherence (PA) Approach

		ITT					PA				
Model	Effect	B	SE	t	*p*	95_CI	B	SE	t	*p*	95_CI
Model 1	(Intercept)	0.31	0.02	13.03	< .001	0.265 - 0.359	0.32	0.02	13.44	< .001	0.275 - 0.369
	TP Treatment	-0.16	0.03	-5.16	< .001	-0.22 - -0.099	-0.18	0.03	-5.70	< .001	-0.238 - -0.116
Model 2	(Intercept)	0.52	0.39	1.32	.188	-0.252 - 1.283	0.48	0.41	1.19	.233	-0.313 - 1.281
	TP Treatment	-0.15	0.04	-3.67	< .001	-0.238 - -0.072	-0.18	0.04	-4.10	< .001	-0.26 - -0.092
	Age	-0.01	0.00	-3.38	.001	-0.015 - -0.004	-0.01	0.00	-3.66	< .001	-0.016 - -0.005
	Ethnicity – Black	0.21	0.09	2.36	.018	0.036 - 0.384	0.24	0.09	2.72	.007	0.068 - 0.417
	Ethnicity – Mixed	0.00	0.10	-0.04	.970	-0.2 - 0.192	0.01	0.10	0.14	.890	-0.181 - 0.208
	Ethnicity – White	0.11	0.08	1.37	.172	-0.048 - 0.269	0.12	0.08	1.53	.127	-0.035 - 0.282
	Ethnicity – Other	-0.08	0.22	-0.36	.722	-0.499 - 0.346	-0.07	0.22	-0.32	.751	-0.493 - 0.355
	Prison – D-Cat	-0.09	0.10	-0.83	.408	-0.287 - 0.117	-0.10	0.10	-0.96	.339	-0.299 - 0.103
	Prison – C-Cat	0.03	0.10	0.31	.755	-0.16 - 0.22	0.01	0.09	0.12	.908	-0.174 - 0.196
	Prison – B-Cat	-0.04	0.10	-0.37	.711	-0.238 - 0.163	-0.05	0.10	-0.50	.619	-0.245 - 0.146
	Prison – A-Cat	0.19	0.19	1.00	.316	-0.185 - 0.571	0.13	0.18	0.70	.483	-0.229 - 0.484
	Copas	0.07	0.03	2.14	.033	0.005 - 0.125	0.06	0.03	1.95	.051	0 - 0.121
	IEP – Standard	-0.13	0.20	-0.66	.513	-0.52 - 0.26	-0.06	0.24	-0.24	.810	-0.518 - 0.405
	IEP – Enhanced	-0.20	0.20	-1.01	.311	-0.587 - 0.187	-0.12	0.23	-0.52	.602	-0.582 - 0.337

Table A12

Linear Models Predicting Adjudications Based on Treatment (Model 1) And Based on Treatment and Matching Parameters (Model 2) Following the Intent to Treat (ITT) and Protocol Adherence (PA) Approach

		ITT					PA				
Model	Effect	B	SE	t	*p*	95_CI	B	SE	t	*p*	95_CI
	Adjudications (pre)	0.21	0.04	4.91	< .001	0.126 - 0.295	0.19	0.04	4.28	< .001	0.102 - 0.274
	Casenote balance (pre)	-0.01	0.01	-0.59	.556	-0.025 - 0.014	-0.01	0.01	-0.68	.495	-0.026 - 0.013
	Self-harm (pre)	0.29	0.08	3.44	.001	0.123 - 0.448	0.25	0.08	3.27	.001	0.101 - 0.404
	IO: Drug offences	0.19	0.31	0.60	.548	-0.428 - 0.806	0.18	0.31	0.58	.562	-0.426 - 0.784
	IO: Fraud offences	0.34	0.37	0.93	.353	-0.379 - 1.062	0.37	0.37	0.99	.323	-0.361 - 1.094
	IO: Miscellaneous crimes against society	0.16	0.33	0.48	.630	-0.489 - 0.808	0.16	0.33	0.49	.624	-0.481 - 0.801
	IO: Possession of weapons	0.15	0.33	0.45	.654	-0.494 - 0.787	0.15	0.32	0.46	.643	-0.481 - 0.778
	IO: Public order offences	0.27	0.35	0.76	.445	-0.419 - 0.953	0.26	0.34	0.77	.441	-0.408 - 0.937
	IO: Robbery	0.20	0.32	0.62	.535	-0.426 - 0.821	0.17	0.31	0.55	.581	-0.44 - 0.785
	IO: Sexual offences	0.40	0.38	1.05	.294	-0.349 - 1.154	0.40	0.38	1.07	.286	-0.337 - 1.142
	IO: Summary motoring	0.79	0.45	1.76	.079	-0.092 - 1.677	0.78	0.44	1.77	.077	-0.085 - 1.643
	IO: Summary non-motoring	0.07	0.42	0.16	.873	-0.759 - 0.894	0.06	0.42	0.15	.883	-0.758 - 0.881
	IO: Theft offences	0.07	0.32	0.20	.839	-0.566 - 0.697	0.06	0.32	0.20	.842	-0.557 - 0.682
	IO: Unknown	0.01	1.05	0.01	.996	-2.047 - 2.059	-0.01	1.15	-0.01	.990	-2.264 - 2.235
	IO: Violence against the person	0.30	0.31	0.94	.345	-0.32 - 0.913	0.28	0.31	0.92	.358	-0.321 - 0.888

Note. P tests are two-tailed. ITT Model 1, $F(1, 2548) = 26.66$, $p < .001$; ITT Model 2, $F(28, 1341) = 4.84$, $p < .001$; PA Model 1, $F(1, 2493) = 32.44$, $p < .001$; ITT Model 2, $F(28, 1291) = 4.49$, $p < .001$

Method to calculate the propensity score

The quality of balance and treatment effects can be sensitive to the method used to estimate propensity scores. As such, we tested logistic regression models to calculate propensity scores and compare the outcomes to the results of the analyses using gradient boosted models (GBM).

Cases with missing data were removed prior to fitting the psm model, which was otherwise identical to the main model, predicting membership in the treatment group based on age, ethnicity, prison type, Copas rate, IEP level, baseline adjudications, baseline case note balance, baseline self-harm incidents, and index offence code. The summary balance statistics (Table A13) show balance improvements compared to unweighted estimates across all indicators, highly similar to the balance statistics of the main model balance based on GBM. The slight improvement regarding the maximum standardised mean differences compared to the main model reflect the lack of missing values, which accounted for the highest SMD levels of the GBM model.

Table A13

Summary of Balance Indicators Using Logistic Regression PSM

	Treatment N	Control N	Control Effective N	Max. SMD	Mean SMD	Max. KS	Mean KS
ITT							
Unw.	606	764	764	1.00	0.29	0.43	0.08
Weigh.	606	764	191.73	0.12	0.04	0.09	0.02
PA							
Unw.	556	764	764	0.98	0.29	0.42	0.08
Weigh.	556	764	190.18	0.11	0.04	0.08	0.02

Note. Results of group balance testing based on mean standardised bias stopping rule for the intent to treat approach and protocol adherence approach.

ITT = Intent to Treat approach, PA = Protocol Adherence approach, Unw = un-weighted, Weigh. = weighted, SMD = standardised mean difference, KS = Kolmogorov Smirnov statistic.

Applying the corresponding weights to the treatment effect models, showed highly similar results to the main models based on the weights extracted via GBM, with significant treatment effects on the number of adjudications post treatment in both the simple and doubly robust models and for both the ITT and PA approach (Table A14).

Table A14

General Linear Model Predicting Adjudications Based on Treatment (Model 1) And Based on Treatment and Matching Parameters (Model 2) Using Weights Extracted From Logistic Regression PSM Following the Intent to Treat (ITT) and Protocol Adherence (PA) Approach

		ITT					PA				
Model	Effect	B	SE	t	*p*	95_CI	B	SE	t	*p*	95_CI
Model 1	(Intercept)	0.33	0.07	4.37	< .001	0.18 - 0.474	0.33	0.07	4.50	< .001	0.188 - 0.48
	TP Treatment	-0.17	0.08	-2.18	.029	-0.324 - -0.017	-0.18	0.08	-2.37	.018	-0.334 - -0.031
Model 2	(Intercept)	0.75	0.25	3.05	.002	0.267 - 1.231	0.73	0.26	2.81	.005	0.222 - 1.245
	TP Treatment	-0.16	0.07	-2.36	.018	-0.298 - -0.028	-0.18	0.07	-2.57	.010	-0.319 - -0.043
	Age	-0.01	0.01	-1.34	.181	-0.025 - 0.005	-0.01	0.01	-1.42	.157	-0.024 - 0.004
	Ethnicity – Black	0.24	0.11	2.08	.038	0.013 - 0.461	0.26	0.12	2.25	.025	0.033 - 0.489
	Ethnicity – Mixed	-0.06	0.08	-0.76	.448	-0.21 - 0.093	-0.05	0.08	-0.65	.515	-0.204 - 0.102
	Ethnicity – White	0.09	0.09	1.04	.299	-0.084 - 0.272	0.10	0.09	1.08	.278	-0.079 - 0.274
	Ethnicity – Other	-0.07	0.10	-0.70	.481	-0.276 - 0.13	-0.07	0.10	-0.71	.476	-0.266 - 0.124
	Prison – D-Cat	-0.26	0.15	-1.74	.081	-0.547 - 0.032	-0.26	0.15	-1.82	.069	-0.55 - 0.021
	Prison – C-Cat	-0.13	0.19	-0.68	.496	-0.497 - 0.241	-0.15	0.18	-0.80	.421	-0.505 - 0.211
	Prison – B-Cat	-0.24	0.15	-1.60	.110	-0.534 - 0.054	-0.24	0.15	-1.63	.103	-0.533 - 0.049
	Prison – A-Cat	0.21	0.53	0.40	.691	-0.834 - 1.258	0.21	0.55	0.39	.696	-0.86 - 1.287
	Copas	0.07	0.04	1.99	.047	0.001 - 0.144	0.07	0.04	1.81	.071	-0.006 - 0.143
	IEP – Standard	-0.14	0.15	-0.88	.379	-0.437 - 0.166	-0.11	0.18	-0.60	.546	-0.457 - 0.242
	IEP – Enhanced	-0.24	0.16	-1.51	.131	-0.547 - 0.071	-0.20	0.18	-1.14	.253	-0.549 - 0.145

Table A14

General Linear Model Predicting Adjudications Based on Treatment (Model 1) And Based on Treatment and Matching Parameters (Model 2) Using Weights Extracted From Logistic Regression PSM Following the Intent to Treat (ITT) and Protocol Adherence (PA) Approach

		ITT					PA				
Model	Effect	B	SE	t	*p*	95_CI	B	SE	t	*p*	95_CI
	Adjudications (pre)	0.16	0.07	2.29	.022	0.022 - 0.288	0.12	0.06	2.23	.026	0.015 - 0.235
	Casenote balance (pre)	0.00	0.01	-0.13	.896	-0.031 - 0.027	0.00	0.01	-0.24	.813	-0.033 - 0.026
	Self-harm (pre)	0.26	0.07	3.75	< .001	0.124 - 0.397	0.27	0.07	3.73	< .001	0.129 - 0.416
	IO: Drug offences	0.20	0.07	2.86	.004	0.063 - 0.339	0.20	0.07	2.88	.004	0.063 - 0.335
	IO: Fraud offences	0.48	0.18	2.65	.008	0.126 - 0.842	0.47	0.18	2.65	.008	0.121 - 0.811
	IO: Miscellaneous crimes against society	0.17	0.10	1.69	.091	-0.028 - 0.374	0.17	0.11	1.52	.128	-0.048 - 0.379
	IO: Possession of weapons	0.10	0.10	1.01	.314	-0.099 - 0.309	0.11	0.11	1.01	.312	-0.1 - 0.312
	IO: Public order offences	0.22	0.13	1.73	.085	-0.03 - 0.465	0.22	0.13	1.72	.086	-0.031 - 0.462
	IO: Robbery	0.21	0.10	2.22	.027	0.024 - 0.401	0.19	0.10	1.94	.052	-0.002 - 0.374
	IO: Sexual offences	0.32	0.32	1.00	.316	-0.306 - 0.945	0.31	0.32	0.97	.335	-0.321 - 0.942
	IO: Summary motoring	0.72	0.31	2.32	.021	0.112 - 1.333	0.74	0.31	2.42	.016	0.14 - 1.34
	IO: Summary non-motoring	0.08	0.10	0.77	.441	-0.122 - 0.281	0.07	0.10	0.72	.472	-0.126 - 0.272
	IO: Theft offences	0.07	0.08	0.78	.433	-0.099 - 0.231	0.07	0.08	0.85	.394	-0.09 - 0.229
	IO: Unknown	0.05	0.11	0.41	.682	-0.177 - 0.27	0.03	0.11	0.26	.796	-0.187 - 0.243
	IO: Violence against the person	0.29	0.09	3.24	.001	0.116 - 0.47	0.28	0.09	3.09	.002	0.101 - 0.455

Note. P tests are two-tailed. ITT Model 1, $F(1, 1368) = 4.76$, $p = .029$; ITT Model 2, $F(28, 1341) = 4.53$, $p < .001$; PA Model 1, $F(1, 1318) = 5.61$, $p = .018$; ITT Model 2, $F(28, 1291) = 4.30$, $p < .001$

Method to calculate control group observation/"treatment" period

We tested alternative approaches to assign the "treatment" start time for the control cases, a) using the median (instead of the mean) treatment-to-release time of the treatment group, b) assigning randomly selected dates within the treatment period to each control case using a bootstrapping procedure (10.000 iterations).

A) We tested the same model as for the main analysis, but used the median to set the average time between treatment start and release for treatment cases (13 months) to calculate the equivalent pre/post-treatment periods for control cases which reduces the sample of eligible control cases to n = 1600. Initially we included the 'time until release' variable as an additional matching parameter to estimate the PSM. The balance summary statistics showed improved but problematic balance levels (Model 1, Table A15). Unsurprisingly, 'time until release' between treatment and control cases was extremely unbalanced (Table A). The extracted 16weights could not overcome this imbalance, and it further led to statistically significant and substantial sized differences between the groups on other covariates (e.g., ethnicity, prison type, Copas rate, IEP-level, self-harm incidents).

Table A15

Summary of Balance Indicators Fitting PSM Based on ITT Using Median Approach to Construct Control Group, Including 'Time Until Release' as Matching Parameter (Model 1) and Excluding 'Time Until Release' as Matching Parameter (Model 2)

	Treatment N	Control N	Control Effective N	Max. SMD	Mean. SMD	Max. KS	Mean.KS	Iterations
Model 1								
Unw.	676	1241	676	1.17	0.30	0.51	0.11	
Weigh.	676	1241	151.16	0.70	0.21	0.50	0.08	1143
Model 2								
Unw.	676	1241	676	1.17	0.30	0.51	0.11	
Weigh.	676	1241	175.25	0.27	0.08	0.09	0.02	1424

Note. Results of group balance testing based on mean standardised bias stopping rule for the intent to treat approach and protocol adherence approach.

ITT = Intent to Treat approach, PA = Protocol Adherence approach, Unw = un-weighted, Weigh. = weighted, SMD = standardised mean difference, KS = Kolmogorov Smirnov statistic.

Thus, we decided to exclude 'time until release' as a matching parameter, and only include it as a covariate in the doubly-robust model testing for treatment effects. The summary of balance indicators (Model 2, Table A15) showed maximum and average balance indicators similar to the main analysis and the individual balance statistics show all matching parameters (other than some NAs) to have standardised mean differences < .20 and distribution differences (KS) < .10 (Table A17). Subsequent models to estimate treatment effects showed effect patterns that were highly similar to the main models (Table A18), where TP participants showed significantly lower levels of adjudications after the treatment period, both in the simple and doubly robust models, and for both the ITT and PA approaches. The effects of covariates were also stable, with the same effect patterns for criminal history covariates (Copas rate, baseline adjudications). Interestingly, 'time until release' was not a significant predictor of adjudications and showed a small negative correlation with the outcome variable (B = -0.0012, SE = 0.0006, p = .090).

Table A16

Balance Indicators After Fitting PSM Based on ITT Using Median Approach to Construct Control Group, Including 'Time Until Release' as Matching Parameter

	Unweighted									Weighted								
	Treatment		Control							Treatment		Control						
Variable	M	SD	M	SD	SMD	t-test/ $\chi2$	SMD *p*	KS	KS *p*	M	SD	M	SD	SMD	t-test/ $\chi2$	SMD *p*	KS	KS *p*
Age	31.17	7.75	34.83	10.40	-0.47	-8.73	< .001	0.18	< .001	31.17	7.75	31.26	9.50	-0.01	-0.12	.907	0.11	.102
Ethnicity - Asian	0.07	0.26	0.08	0.27	0.00	14.55	< .001	0.00	< .001	0.07	0.26	0.06	0.24	0.05	4.57	.003	0.01	.003
Ethnicity – Black	0.22	0.41	0.10	0.30	0.27			0.11		0.22	0.41	0.33	0.47	-0.28			0.12	
Ethnicity – Mixed	0.09	0.29	0.05	0.22	0.14			0.04		0.09	0.29	0.04	0.20	0.17			0.05	
Ethnicity – White	0.59	0.49	0.74	0.44	-0.31			0.15		0.59	0.49	0.55	0.50	0.09			0.04	
Ethnicity – Other	0.01	0.09	0.02	0.14	-0.12			0.01		0.01	0.09	0.01	0.10	-0.02			0.00	
Ethnicity - NA	0.02	0.13	0.00	0.07	0.09			0.01		0.02	0.13	0.00	0.05	0.11			0.01	
Prison – Yoi	0.06	0.23	0.11	0.32	-0.24	28.81	< .001	0.06	< .001	0.06	0.23	0.22	0.41	-0.70	17.42	< .001	0.16	< .001
Prison – D-Cat	0.24	0.42	0.07	0.26	0.38			0.16		0.24	0.42	0.11	0.32	0.29			0.12	
Prison – C-Cat	0.42	0.49	0.54	0.50	-0.23			0.11		0.42	0.49	0.44	0.50	-0.04			0.02	
Prison – B-Cat	0.27	0.44	0.27	0.44	0.00			0.00		0.27	0.44	0.21	0.41	0.13			0.06	
Prison – A-Cat	0.02	0.13	0.01	0.11	0.05			0.01		0.02	0.13	0.02	0.13	0.01			0.00	
Copas	-0.98	0.78	-0.56	0.81	-0.55	-10.95	< .001	0.22	< .001	-0.98	0.78	-0.48	0.77	-0.65	-8.15	< .001	0.28	< .001
Copas - NA	0.05	0.22	0.07	0.26	-0.09	-1.71	.088	0.02	.997	0.05	0.22	0.04	0.19	0.06	1.07	.286	0.01	1.000
IEP – Basic	0.01	0.09	0.05	0.21	-0.40	173.79	< .001	0.04	< .001	0.01	0.09	0.07	0.25	-0.61	18.94	< .001	0.06	< .001
IEP – Standard	0.21	0.41	0.70	0.46	-1.17			0.48		0.21	0.41	0.43	0.50	-0.52			0.22	

Table A16

Balance Indicators After Fitting PSM Based on ITT Using Median Approach to Construct Control Group, Including 'Time Until Release' as Matching Parameter

	Unweighted									Weighted								
	Treatment		Control							Treatment		Control						
Variable	M	SD	M	SD	SMD	t-test/ χ2	SMD *p*	KS	KS *p*	M	SD	M	SD	SMD	t-test/ χ2	SMD *p*	KS	KS *p*
IEP – Enhanced	0.71	0.46	0.19	0.39	1.13			0.52		0.71	0.46	0.46	0.50	0.54			0.24	
IEP – NA	0.07	0.25	0.06	0.25	0.02			0.00		0.07	0.25	0.04	0.20	0.11			0.03	
Adjudications (pre)	0.12	0.45	0.23	0.91	-0.24	-3.46	.001	0.03	.928	0.12	0.45	0.35	1.14	-0.51	-2.98	.003	0.06	.673
Casenote balance (pre)	0.66	2.27	-0.43	2.22	0.48	8.78	< .001	0.22	< .001	0.66	2.27	0.65	2.57	0.00	0.03	.973	0.19	.001
Casenote balance (pre) - NA	0.00		0.48	0.50		-34.02	< .001	0.48	< .001	0.00		0.12	0.33		-5.34	< .001	0.12	.041
Self-harm (pre)	0.01	0.21	0.10	0.96	-0.43	-3.17	.002	0.03	.836	0.01	0.21	0.10	0.80	-0.42	-3.06	.002	0.04	.967
IO: Criminal damage and arson	0.00	0.07	0.01	0.12	-0.16	20.83	< .001	0.01	< .001	0.00	0.07	0.01	0.10	-0.08	49.55	< .001	0.00	< .001
IO: Drug offences	0.32	0.47	0.14	0.34	0.40			0.19		0.32	0.47	0.32	0.47	0.00			0.00	
IO: Fraud offences	0.01	0.11	0.00	0.07	0.06			0.01		0.01	0.11	0.00	0.04	0.09			0.01	
IO: Miscellaneous crimes against society	0.04	0.18	0.06	0.23	-0.12			0.02		0.04	0.18	0.04	0.18	0.00			0.00	
IO: Possession of weapons	0.05	0.21	0.07	0.25	-0.09			0.02		0.05	0.21	0.04	0.20	0.02			0.00	
IO: Public order offences	0.02	0.14	0.10	0.29	-0.56			0.08		0.02	0.14	0.06	0.23	-0.27			0.04	
IO: Robbery	0.12	0.32	0.06	0.24	0.17			0.06		0.12	0.32	0.10	0.30	0.06			0.02	
IO: Sexual offences	0.01	0.10	0.05	0.23	-0.43			0.04		0.01	0.10	0.02	0.15	-0.13			0.01	
IO: Summary motoring	0.01	0.08	0.02	0.14	-0.17			0.01		0.01	0.08	0.01	0.07	0.00			0.00	
IO: Summary non-motoring	0.01	0.09	0.04	0.19	-0.36			0.03		0.01	0.09	0.02	0.14	-0.16			0.01	

Table A16

Balance Indicators After Fitting PSM Based on ITT Using Median Approach to Construct Control Group, Including 'Time Until Release' as Matching Parameter

	Unweighted									Weighted								
	Treatment		Control							Treatment		Control						
Variable	M	SD	M	SD	SMD	t-test/ χ2	SMD *p*	KS	KS *p*	M	SD	M	SD	SMD	t-test/ χ2	SMD *p*	KS	KS *p*
IO: Theft offences	0.07	0.26	0.19	0.39	-0.45			0.12		0.07	0.26	0.14	0.35	-0.26			0.07	
IO: Unknown	0.00		0.00	0.06				0.00		0.00		0.00	0.04				0.00	
IO: Violence against the person	0.31	0.46	0.26	0.44	0.12			0.06		0.31	0.46	0.24	0.42	0.16			0.08	
IO: <NA>	0.03	0.17	0.00		0.17			0.03		0.03	0.17	0.00		0.17			0.03	
Time until release	18.89	27.98	12.97	0.02	0.21	5.05	< .001	0.50	< .001	18.89	27.98	12.97	0.02	0.21	5.06	< .001	0.50	< .001
Time until release: <NA>	0.16	0.36	0.00		0.43	11.15	< .001	0.16	< .001	0.16	0.36	0.00		0.43	11.15	< .001	0.16	.005

Table A17

Balance Indicators After Fitting PSM Based on ITT Using Median Approach to Construct Control Group, not Including 'Time Until Release' as Matching Parameter

	Unweighted									Weighted								
	Treatment		Control							Treatment		Control						
Variable	M	SD	M	SD	SMD	t-test/ $\chi2$	SMD *p*	KS	KS *p*	M	SD	M	SD	SMD	t-test/ $\chi2$	SMD *p*	KS	KS *p*
Age	31.17	7.75	34.83	10.40	-0.47	-8.73	< .001	0.18	< .001	31.17	7.75	31.75	7.68	-0.07	-1.08	.278	0.05	.847
Ethnicity - Asian	0.07	0.26	0.08	0.27	0.00	14.55	< .001	0.00	< .001	0.07	0.26	0.09	0.28	-0.04	1.12	.344	0.01	.344
Ethnicity – Black	0.22	0.41	0.10	0.30	0.27		< .001	0.11	< .001	0.22	0.41	0.17	0.37	0.12			0.05	
Ethnicity – Mixed	0.09	0.29	0.05	0.22	0.14		< .001	0.04	< .001	0.09	0.29	0.12	0.32	-0.08			0.02	
Ethnicity – White	0.59	0.49	0.74	0.44	-0.31		< .001	0.15	< .001	0.59	0.49	0.62	0.49	-0.05			0.03	
Ethnicity – Other	0.01	0.09	0.02	0.14	-0.12		< .001	0.01	< .001	0.01	0.09	0.01	0.12	-0.05			0.00	
Ethnicity - NA	0.02	0.13	0.00	0.07	0.09		< .001	0.01	< .001	0.02	0.13	0.00	0.06	0.10			0.01	
Prison – Yoi	0.06	0.23	0.11	0.32	-0.24	28.81	< .001	0.06	< .001	0.06	0.23	0.06	0.23	0.01	1.09	.355	0.00	.355
Prison – D-Cat	0.24	0.42	0.07	0.26	0.38		< .001	0.16	< .001	0.24	0.42	0.27	0.44	-0.08			0.04	
Prison – C-Cat	0.42	0.49	0.54	0.50	-0.23		< .001	0.11	< .001	0.42	0.49	0.46	0.50	-0.08			0.04	
Prison – B-Cat	0.27	0.44	0.27	0.44	0.00		< .001	0.00	< .001	0.27	0.44	0.20	0.40	0.16			0.07	
Prison – A-Cat	0.02	0.13	0.01	0.11	0.05		< .001	0.01	< .001	0.02	0.13	0.01	0.12	0.02			0.00	
Copas	-0.98	0.78	-0.56	0.81	-0.55	-10.95	< .001	0.22	< .001	-0.98	0.78	-0.86	0.71	-0.15	-2.11	.035	0.09	.222
Copas - NA	0.05	0.22	0.07	0.26	-0.09	-1.71	< .001	0.02	.997	0.05	0.22	0.02	0.14	0.14	2.90	.004	0.03	.999
IEP – Basic	0.01	0.09	0.05	0.21	-0.40	173.79	< .001	0.04	< .001	0.01	0.09	0.01	0.12	-0.06	17.48	< .001	0.01	< .001
IEP – Standard	0.21	0.41	0.70	0.46	-1.17			0.48		0.21	0.41	0.26	0.44	-0.10			0.04	

Table A17

Balance Indicators After Fitting PSM Based on ITT Using Median Approach to Construct Control Group, not Including 'Time Until Release' as Matching Parameter

	Unweighted									Weighted								
	Treatment		Control							Treatment		Control						
Variable	M	SD	M	SD	SMD	t-test/ χ2	SMD *p*	KS	KS *p*	M	SD	M	SD	SMD	t-test/ χ2	SMD *p*	KS	KS *p*
IEP – Enhanced	0.71	0.46	0.19	0.39	1.13			0.52		0.71	0.46	0.73	0.44	-0.05			0.02	
IEP – NA	0.07	0.25	0.06	0.25	0.02			0.00		0.07	0.25	0.00	0.04	0.27			0.07	
Adjudications (pre)	0.12	0.45	0.23	0.91	-0.24	-3.46	.001	0.03	.928	0.12	0.45	0.15	0.60	-0.05	-0.78	.434	0.01	1.000
Casenote balance (pre)	0.66	2.27	-0.43	2.22	0.48	8.78	< .001	0.22	< .001	0.66	2.27	0.65	2.13	0.00	0.07	.945	0.07	.540
Casenote balance (pre) - NA	0.00		0.48	0.50		-34.02	< .001	0.48	< .001	0.00		0.00	0.07		-10.80	< .001	0.00	1.000
Self-harm (pre)	0.01	0.21	0.10	0.96	-0.43	-3.17	.002	0.03	.836	0.01	0.21	0.03	0.65	-0.10	-1.22	.222	0.00	1.000
IO: Criminal damage and arson	0.00	0.07	0.01	0.12	-0.16	20.83	< .001	0.01	< .001	0.00	0.07	0.01	0.12	-0.15	1.49	.139	0.01	.139
IO: Drug offences	0.32	0.47	0.14	0.34	0.40			0.19		0.32	0.47	0.33	0.47	-0.02			0.01	
IO: Fraud offences	0.01	0.11	0.00	0.07	0.06			0.01		0.01	0.11	0.00	0.06	0.08			0.01	
IO: Miscellaneous crimes against society	0.04	0.18	0.06	0.23	-0.12			0.02		0.04	0.18	0.03	0.16	0.05			0.01	
IO: Possession of weapons	0.05	0.21	0.07	0.25	-0.09			0.02		0.05	0.21	0.04	0.19	0.04			0.01	
IO: Public order offences	0.02	0.14	0.10	0.29	-0.56			0.08		0.02	0.14	0.03	0.16	-0.05			0.01	
IO: Robbery	0.12	0.32	0.06	0.24	0.17			0.06		0.12	0.32	0.10	0.31	0.05			0.01	
IO: Sexual offences	0.01	0.10	0.05	0.23	-0.43			0.04		0.01	0.10	0.01	0.11	-0.03			0.00	

Table A17

Balance Indicators After Fitting PSM Based on ITT Using Median Approach to Construct Control Group, not Including 'Time Until Release' as Matching Parameter

	Unweighted									Weighted								
	Treatment		Control							Treatment		Control						
Variable	M	SD	M	SD	SMD	t-test/ χ2	SMD *p*	KS	KS *p*	M	SD	M	SD	SMD	t-test/ χ2	SMD *p*	KS	KS *p*
IO: Summary motoring	0.01	0.08	0.02	0.14	-0.17			0.01		0.01	0.08	0.00	0.06	0.02			0.00	
IO: Summary non-motoring	0.01	0.09	0.04	0.19	-0.36			0.03		0.01	0.09	0.00	0.06	0.04			0.00	
IO: Theft offences	0.07	0.26	0.19	0.39	-0.45			0.12		0.07	0.26	0.07	0.26	-0.01			0.00	
IO: Unknown	0.00		0.00	0.06				0.00		0.00		0.00	0.02				0.00	
IO: Violence against the person	0.31	0.46	0.26	0.44	0.12			0.06		0.31	0.46	0.35	0.48	-0.09			0.04	
IO: <NA>	0.03	0.17	0.00		0.17			0.03		0.03	0.17	0.00		0.17			0.03	

Table A18

General Linear Model Predicting Adjudications Based on Treatment (Model 1) And Based on Treatment And Matching Covariates (Model 2) Applying Weights Extracted From Median-Based Control Group Design Following the Intent To Treat (ITT) and Protocol Adherence (PA) Approach

		ITT					PA				
Model	Effect	B	SE	t	*p*	95_CI	B	SE	t	*p*	95_CI
Model 1	(Intercept)	0.39	0.08	4.72	< .001	0.225 - 0.546	0.41	0.09	4.42	< .001	0.228 - 0.59
	TP Treatment	-0.23	0.08	-2.77	.006	-0.398 - -0.068	-0.26	0.09	-2.79	.005	-0.449 - -0.079
Model 2	(Intercept)	0.70	0.39	1.79	.074	-0.068 - 1.463	0.67	0.41	1.64	.101	-0.13 - 1.465
	TP Treatment	-0.21	0.09	-2.45	.014	-0.377 - -0.042	-0.25	0.09	-2.62	.009	-0.432 - -0.062
	Age	0.00	0.00	-1.26	.210	-0.012 - 0.003	0.00	0.00	-1.22	.224	-0.011 - 0.003
	Ethnicity – Black	-0.21	0.30	-0.69	.489	-0.8 - 0.383	-0.26	0.36	-0.73	.464	-0.961 - 0.438
	Ethnicity – Mixed	-0.19	0.30	-0.65	.518	-0.784 - 0.395	-0.25	0.35	-0.72	.471	-0.946 - 0.438
	Ethnicity – White	-0.20	0.29	-0.70	.487	-0.765 - 0.365	-0.27	0.34	-0.79	.428	-0.938 - 0.398
	Ethnicity – Other	-0.37	0.31	-1.21	.225	-0.975 - 0.23	-0.43	0.36	-1.20	.231	-1.132 - 0.274
	Prison – D-Cat	0.06	0.07	0.90	.368	-0.07 - 0.189	0.05	0.07	0.77	.442	-0.079 - 0.182
	Prison – C-Cat	0.15	0.07	2.07	.038	0.008 - 0.297	0.13	0.07	1.79	.073	-0.012 - 0.263
	Prison – B-Cat	0.40	0.16	2.52	.012	0.087 - 0.703	0.42	0.16	2.61	.009	0.103 - 0.728
	Prison – A-Cat	0.74	0.39	1.89	.059	-0.028 - 1.512	0.77	0.40	1.92	.055	-0.016 - 1.554
	Copas	0.14	0.06	2.26	.024	0.018 - 0.253	0.13	0.06	2.18	.030	0.013 - 0.248
	IEP – Standard	-0.44	0.29	-1.53	.126	-1.009 - 0.124	-0.35	0.23	-1.53	.126	-0.79 - 0.098
	IEP – Enhanced	-0.45	0.27	-1.66	.097	-0.984 - 0.082	-0.36	0.21	-1.68	.094	-0.78 - 0.061

Table A18

General Linear Model Predicting Adjudications Based on Treatment (Model 1) And Based on Treatment And Matching Covariates (Model 2) Applying Weights Extracted From Median-Based Control Group Design Following the Intent To Treat (ITT) and Protocol Adherence (PA) Approach

		ITT					PA				
Model	Effect	B	SE	t	*p*	95_CI	B	SE	t	*p*	95_CI
	Adjudications (pre)	0.26	0.11	2.30	.022	0.037 - 0.475	0.17	0.07	2.32	.021	0.026 - 0.312
	Casenote balance (pre)	0.00	0.02	-0.13	.897	-0.034 - 0.03	-0.01	0.02	-0.34	.731	-0.038 - 0.027
	Self-harm (pre)	0.17	0.10	1.61	.107	-0.036 - 0.374	0.23	0.08	2.97	.003	0.076 - 0.374
	IO: Drug offences	0.41	0.14	2.89	.004	0.131 - 0.681	0.44	0.15	2.84	.005	0.135 - 0.74
	IO: Fraud offences	0.35	0.23	1.53	.126	-0.1 - 0.807	0.38	0.25	1.52	.128	-0.108 - 0.858
	IO: Miscellaneous crimes against society	0.13	0.13	0.99	.320	-0.13 - 0.398	0.18	0.15	1.19	.234	-0.118 - 0.481
	IO: Possession of weapons	0.32	0.13	2.47	.014	0.066 - 0.574	0.35	0.14	2.53	.011	0.078 - 0.612
	IO: Public order offences	0.18	0.12	1.53	.125	-0.051 - 0.418	0.22	0.13	1.73	.084	-0.03 - 0.467
	IO: Robbery	0.45	0.18	2.51	.012	0.099 - 0.81	0.47	0.19	2.43	.015	0.09 - 0.842
	IO: Sexual offences	0.51	0.30	1.68	.094	-0.086 - 1.102	0.58	0.33	1.74	.083	-0.075 - 1.231
	IO: Summary motoring	0.66	0.32	2.03	.043	0.021 - 1.289	0.65	0.32	2.00	.045	0.014 - 1.281
	IO: Summary non-motoring	0.23	0.11	2.16	.031	0.021 - 0.433	0.26	0.12	2.12	.034	0.02 - 0.495
	IO: Theft offences	0.24	0.12	2.00	.045	0.005 - 0.474	0.30	0.13	2.34	.019	0.048 - 0.543
	IO: Unknown	1.15	1.39	0.83	.409	-1.584 - 3.887	1.04	1.25	0.83	.404	-1.405 - 3.485
	IO: Violence against the person	0.38	0.13	2.89	.004	0.124 - 0.644	0.40	0.13	3.02	.003	0.14 - 0.66
	Time until release	0.00	0.00	-1.70	.090	-0.003 - 0	0.00	0.00	-1.49	.137	-0.003 - 0

Table A18

General Linear Model Predicting Adjudications Based on Treatment (Model 1) And Based on Treatment And Matching Covariates (Model 2) Applying Weights Extracted From Median-Based Control Group Design Following the Intent To Treat (ITT) and Protocol Adherence (PA) Approach

		ITT					PA				
Model	Effect	B	SE	t	*p*	95_CI	B	SE	t	*p*	95_CI

Note. P tests are two-tailed. ITT Model 1, *F*(1, 1915) = 7.66, p = *.006*; ITT Model 2, *F*(29, 1104) = 2.76, $p < .001$; PA Model 1, *F*(1, 1860) = 7.80, p = .005; ITT Model 2, *F*(29, 1056) = 2.82, $p < .001$.

B) Lastly, we estimated a propensity score model where the control group observation (i.e., “treatment”) period was based on randomly selected dates for each case within the research period. We ran 1000 iterations of this model using a bootstrap loop and calculated average balance and treatment effects. We present average estimates, p-values as well as the percentage of results with $p < .05$, to indicate the frequency of significant effects.

Similar to the median-based approach, we first estimated the PSM to predict membership in the treatment group based on demographic characteristics, criminal history and prison behaviour background, including the ‘time until release’ as a matching parameter. Unlike the median-based models, the models utilising randomly generated control group observation periods produced balance overall (Table A19), with averaged standardised mean differences below <0.20 and average mean KS scores <0.10.

Table A19

Summary Of Balance Indicators Fitting PSM Using Control Group Based on 1.000 Random Observation Period Calculations

	Treatment N	Control N	Control Effective N	Max. SMD	Mean SMD	Max. KS	Mean KS	Iterations
ITT								
Unw.	676	4334	4334	1.21	0.27	0.58	0.10	
Weigh.	676	4334	356.98	0.36	0.08	0.40	0.03	5022.72
PA								
Unw.	621	4334	4334	1.19	0.26	0.58	0.10	
Weigh.	621	4334	358.32	0.34	0.07	0.40	0.03	4910.61

Note. Results of group balance testing based on mean standardised bias stopping rule for the intent to treat approach and protocol adherence approach.

ITT = Intent to Treat approach, PA = Protocol Adherence approach, Unw = un-weighted, Weigh. = weighted, SMD = standardised mean difference, KS = Kolmogorov Smirnov statistic.

Moreover, the balance metrics of individual parameters show that the weights extracted led to good balance for all but the ‘time until release’ variable (Tables A20 and A21), which had been fixed to be equal in the main model. ‘Time until release’ as a covariate in the doubly robust model did not correlate with the outcome variable. The models estimating the treatment effects (Table A22) show that the treatment effect of Twinning Project was statistically significant in the vast majority of the simple effect models, following both the ITT and PA approaches (85% and 92% respectively). However, the treatment effect was significant in only 33% and 49% of the doubly robust models. Among the covariates in the doubly-robust models, Copas rate, baseline adjudications and certain index offences (drug offences, robbery offences, violence against the person offences) were the most reliable predictors ($p < .05 > = 75\%$).

Table A20

Average Balance Indicators of 1.000 PSM Based on ITT Using a Randomised Approach to Construct Control Group, Including 'Time Until Release' as Matching Parameter

	Unweighted									Weighted								
	Treatment		Control							Treatment		Control						
Variable	M	SD	M	SD	SMD	t-test/ χ2	SMD *p*	KS	KS *p*	M	SD	M	SD	SMD	t-test/ χ2	SMD *p*	KS	KS *p*
Age	31.05	7.72	35.45	11.423	-0.57	-12.76	< .001	0.12	0.000	31.05	7.72	32.05	8.14	-0.13	-2.19	.045	0.06	.473
Ethnicity - Asian	0.08	0.26	0.08	0.274	-0.03	18.03	< .001	0.01	0.000	0.08	0.26	0.08	0.27	-0.03	1.06	.391	0.01	.391
Ethnicity – Black	0.22	0.41	0.13	0.331	0.22			0.09		0.22	0.41	0.21	0.41	0.01			0.01	
Ethnicity – Mixed	0.09	0.29	0.05	0.217	0.14			0.04		0.09	0.29	0.10	0.30	-0.03			0.01	
Ethnicity – White	0.59	0.49	0.72	0.446	-0.27			0.13		0.59	0.49	0.59	0.49	-0.00			0.01	
Ethnicity – Other	0.01	0.09	0.02	0.124	-0.07			0.01		0.01	0.09	0.01	0.09	0.01			0.00	
Ethnicity - NA	0.02	0.13	0.00	0.055	0.11			0.01		0.02	0.13	0.00	0.05	0.11			0.01	
Prison – Yoi	0.06	0.23	0.15	0.358	-0.41	53.23	< .001	0.10	0.000	0.06	0.23	0.07	0.25	-0.05	1.24	.343	0.01	.343
Prison – D-Cat	0.24	0.42	0.07	0.257	0.39			0.16		0.24	0.42	0.22	0.41	0.04			0.02	
Prison – C-Cat	0.42	0.49	0.48	0.497	-0.11			0.06		0.42	0.49	0.46	0.50	-0.09			0.04	
Prison – B-Cat	0.27	0.44	0.27	0.444	-0.02			0.01		0.27	0.44	0.22	0.42	0.09			0.04	
Prison – A-Cat	0.02	0.13	0.03	0.158	-0.06			0.01		0.02	0.13	0.02	0.15	-0.03			0.00	
Copas	-0.98	0.78	-0.80	0.836	-0.23	-5.30	< .001	0.11	0.000	-0.98	0.78	-0.97	0.76	-0.02	-0.23	.743	0.04	.868
Copas - NA	0.05	0.22	0.11	0.314	-0.27	-6.08	< .001	0.06	0.031	0.05	0.22	0.03	0.18	0.09	1.91	.077	0.02	.996
IEP – Basic	0.01	0.09	0.04	0.193	-0.32	271.02	< .001	0.03	< .001	0.01	0.09	0.02	0.13	-0.10	12.18	.037	0.01	.037
IEP – Standard	0.21	0.41	0.71	0.451	-1.21			0.50		0.21	0.41	0.25	0.43	-0.09			0.04	
IEP – Enhanced	0.70	0.45	0.20	0.396	1.12			0.51		0.70	0.45	0.72	0.45	-0.04			0.02	

Table A20

Average Balance Indicators of 1.000 PSM Based on ITT Using a Randomised Approach to Construct Control Group, Including 'Time Until Release' as Matching Parameter

	Unweighted									Weighted								
	Treatment		Control							Treatment		Control						
Variable	M	SD	M	SD	SMD	t-test/ χ2	SMD *p*	KS	KS *p*	M	SD	M	SD	SMD	t-test/ χ2	SMD *p*	KS	KS *p*
IEP – NA	0.07	0.25	0.05	0.220	0.07			0.02		0.07	0.25	0.01	0.06	0.25			0.06	
Adjudications (pre)	0.12	0.45	0.19	0.811	-0.15	-3.08	0.004	0.02	0.963	0.12	0.45	0.16	0.59	-0.07	-1.36	.215	0.01	.995
Casenote balance (pre)	0.65	2.26	-0.19	2.737	0.37	7.77	< .001	0.14	< .001	0.65	2.26	0.53	2.15	0.06	1.02	.364	0.04	.750
Casenote balance (pre) - NA	0.00		0.58	0.492		-76.97	< .001	0.58	< .001	0.00		0.00	0.02		- 10.10	.001	0.00	.996
Self-harm (pre)	0.01	0.21	0.09	1.129	-0.39	-4.28	< .001	0.02	0.951	0.01	0.21	0.03	0.63	-0.09	-1.51	.151	0.00	.996
IO: Criminal damage and arson	0.00	0.07	0.02	0.153	-0.30	20.82	< .001	0.02	< .001	0.00	0.07	0.00	0.05	0.02	2.22	.023	0.00	.023
IO: Drug offences	0.32	0.47	0.19	0.391	0.28			0.13		0.32	0.47	0.30	0.46	0.04			0.02	
IO: Fraud offences	0.01	0.11	0.01	0.101	0.01			0.00		0.01	0.11	0.01	0.08	0.05			0.01	
IO: Miscellaneous crimes against society	0.04	0.18	0.03	0.179	0.01			0.00		0.04	0.18	0.03	0.16	0.05			0.01	
IO: Possession of weapons	0.05	0.21	0.04	0.191	0.04			0.01		0.05	0.21	0.04	0.20	0.02			0.01	
IO: Public order offences	0.02	0.14	0.05	0.215	-0.22			0.03		0.02	0.14	0.01	0.12	0.03			0.01	
IO: Robbery	0.12	0.32	0.10	0.292	0.08			0.03		0.12	0.32	0.12	0.33	-0.01			0.01	
IO: Sexual offences	0.01	0.10	0.09	0.285	-0.79			0.08		0.01	0.10	0.01	0.09	0.01			0.00	
IO: Summary motoring	0.01	0.08	0.01	0.090	-0.03			0.00		0.01	0.08	0.00	0.05	0.04			0.00	
IO: Summary non-motoring	0.01	0.09	0.02	0.134	-0.13			0.01		0.01	0.09	0.00	0.06	0.04			0.00	

Table A20

Average Balance Indicators of 1.000 PSM Based on ITT Using a Randomised Approach to Construct Control Group, Including 'Time Until Release' as Matching Parameter

	Unweighted									Weighted								
	Treatment		Control							Treatment		Control						
Variable	M	SD	M	SD	SMD	t-test/ χ2	SMD *p*	KS	KS *p*	M	SD	M	SD	SMD	t-test/ χ2	SMD *p*	KS	KS *p*
IO: Theft offences	0.07	0.26	0.12	0.326	-0.19			0.05		0.07	0.26	0.08	0.26	-0.01			0.01	
IO: Unknown	0.00		0.00	0.057				0.00		0.00		0.00	0.02				0.00	
IO: Violence against the person	0.31	0.46	0.32	0.464	-0.01			0.01		0.31	0.46	0.37	0.49	-0.16			0.08	
IO: <NA>	0.03	0.17	0.00		0.17			0.03		0.03	0.17	0.00		0.17			0.03	
Time until release	18.81	27.87	32.36	26.544	-0.48	-10.72	< .001	0.45	< .001	18.81	27.87	28.84	22.35	-0.36	-6.14	< .001	0.40	< .001
Time until release: <NA>	0.15	0.36	0.28	0.449	-0.36	-8.29	< .001	0.13	< .001	0.15	0.36	0.21	0.41	-0.16	-2.31	.027	0.06	.474

Table A21

Average Balance Indicators of 1.000 PSM Based on PA Using a Randomised Approach to Construct Control Group, Including 'Time Until Release' as Matching Parameter

	Unweighted									Weighted								
	Treatment		Control							Treatment		Control						
Variable	M	SD	M	SD	SMD	t-test/ $\chi 2$	SMD p	KS	KS p	M	SD	M	SD	SMD	t-test/ $\chi 2$	SMD p	KS	KS p
Age	31.08	7.84	35.59	11.47	-0.58	-12.54	< .001	0.20	< .001	31.08	7.84	32.24	8.24	-0.15	-2.48	.022	0.06	.357
Ethnicity - Asian	0.08	0.27	0.08	0.28	-0.01	18.56	< .001	0.00	< .001	0.08	0.27	0.08	0.28	-0.02	0.79	.550	0.01	.550
Ethnicity – Black	0.22	0.42	0.13	0.33	0.23			0.10		0.22	0.42	0.22	0.41	0.01			0.01	
Ethnicity – Mixed	0.10	0.30	0.05	0.22	0.16			0.05		0.10	0.30	0.11	0.31	-0.03			0.01	
Ethnicity – White	0.58	0.49	0.72	0.45	-0.30			0.15		0.58	0.49	0.58	0.49	-0.01			0.01	
Ethnicity – Other	0.01	0.10	0.02	0.12	-0.06			0.01		0.01	0.10	0.01	0.09	0.00			0.00	
Ethnicity - NA	0.01	0.12	0.00	0.06	0.10			0.01		0.01	0.12	0.00	0.06	0.09			0.01	
Prison – Yoi	0.06	0.24	0.15	0.36	-0.38	35.88	< .001	0.09	< .001	0.06	0.24	0.07	0.26	-0.04	0.83	.531	0.01	.531
Prison – D-Cat	0.21	0.40	0.07	0.26	0.33			0.13		0.21	0.40	0.20	0.40	0.01			0.01	
Prison – C-Cat	0.44	0.50	0.48	0.50	-0.08			0.04		0.44	0.50	0.47	0.50	-0.06			0.03	
Prison – B-Cat	0.28	0.45	0.27	0.45	0.01			0.01		0.28	0.45	0.24	0.43	0.09			0.04	
Prison – A-Cat	0.02	0.14	0.03	0.16	-0.05			0.01		0.02	0.14	0.02	0.15	-0.03			0.00	
Copas	-0.99	0.79	-0.80	0.84	-0.23	-5.24	< .001	0.11	< .001	-0.99	0.785	-0.98	0.76	-0.01	-0.19	.763	0.04	.879
Copas - NA	0.05	0.22	0.11	0.32	-0.27	-5.95	< .001	0.06	.040	0.05	0.221	0.03	0.16	0.11	2.34	.024	0.02	.999
IEP – Basic	0.01	0.08	0.04	0.19	-0.41	250.89	< .001	0.03	< .001	0.01	0.08	0.01	0.11	-0.09	11.53	.029	0.01	.029
IEP – Standard	0.22	0.42	0.71	0.45	-1.19			0.49		0.22	0.42	0.26	0.44	-0.08			0.03	
IEP – Enhanced	0.70	0.46	0.20	0.40	1.10			0.50		0.70	0.46	0.73	0.45	-0.06			0.03	

Table A21

Average Balance Indicators of 1.000 PSM Based on PA Using a Randomised Approach to Construct Control Group, Including 'Time Until Release' as Matching Parameter

	Unweighted									Weighted								
	Treatment		Control							Treatment		Control						
Variable	M	SD	M	SD	SMD	t-test/ χ2	SMD *p*	KS	KS *p*	M	SD	M	SD	SMD	t-test/ χ2	SMD *p*	KS	KS *p*
IEP – NA	0.07	0.26	0.05	0.22	0.08			0.02		0.07	0.26	0.01	0.06	0.26			0.07	
Adjudications (pre)	0.13	0.45	0.19	0.81	-0.14	-2.88	0.01	0.02	.960	0.13	0.45	0.157	0.59	-0.06	-1.05	.333	0.01	1.000
Casenote balance (pre)	0.68	2.31	-0.19	2.75	0.38	7.73	< .001	0.14	< .001	0.68	2.31	0.55	2.19	0.06	0.99	.370	0.04	.805
Casenote balance (pre) - NA	0.00		0.58	0.49		-77.24	< .001	0.58	< .001	0.00		0.00	0.02		-10.40	.001	0.00	1.000
Self-harm (pre)	0.01	0.22	0.097	1.13	-0.37	-4.17	< .001	0.02	.975	0.01	0.22	0.04	0.65	-0.09	-1.43	.175	0.00	1.000
IO: Criminal damage and arson	0.01	0.07	0.02	0.15	-0.28	19.88	< .001	0.02	< .001	0.01	0.07	0.00	0.05	0.03	2.20	.023	0.00	.024
IO: Drug offences	0.32	0.47	0.19	0.39	0.28			0.13		0.32	0.47	0.30	0.45	0.04			0.02	
IO: Fraud offences	0.01	0.11	0.01	0.10	0.01			0.00		0.01	0.11	0.01	0.08	0.05			0.01	
IO: Miscellaneous crimes against society	0.03	0.18	0.03	0.18	-0.01			0.00		0.03	0.18	0.02	0.15	0.05			0.01	
IO: Possession of weapons	0.05	0.22	0.04	0.19	0.05			0.01		0.05	0.22	0.05	0.21	0.02			0.02	
IO: Public order offences	0.02	0.14	0.05	0.22	-0.20			0.03		0.02	0.14	0.02	0.12	0.04			0.00	
IO: Robbery	0.11	0.32	0.10	0.29	0.06			0.02		0.11	0.32	0.12	0.32	-0.02			0.01	
IO: Sexual offences	0.01	0.11	0.09	0.29	-0.75			0.08		0.01	0.11	0.01	0.10	0.02			0.00	
IO: Summary motoring	0.01	0.08	0.01	0.09	-0.02			0.00		0.01	0.08	0.00	0.05	0.04			0.00	
IO: Summary non-motoring	0.01	0.09	0.02	0.14	-0.12			0.01		0.01	0.09	0.00	0.07	0.04			0.00	

Table A21

Average Balance Indicators of 1.000 PSM Based on PA Using a Randomised Approach to Construct Control Group, Including ‘Time Until Release’ as Matching Parameter

	Unweighted									Weighted								
	Treatment		Control							Treatment		Control						
Variable	M	SD	M	SD	SMD	t-test/ χ2	SMD *p*	KS	KS *p*	M	SD	M	SD	SMD	t-test/ χ2	SMD *p*	KS	KS *p*
IO: Theft offences	0.08	0.26	0.12	0.33	-0.17			0.05		0.08	0.26	0.08	0.27	-0.01			0.01	
IO: Unknown	0.00		0.00	0.06				0.00		0.00		0.00	0.02				0.00	
IO: Violence against the person	0.31	0.46	0.32	0.47	-0.01			0.00		0.31	0.46	0.39	0.49	-0.16			0.08	
IO: <NA>	0.03	0.17	0.00		0.18			0.03		0.03	0.17	0.00		0.18			0.03	
Time until release	19.38	28.55	32.49	26.65	-0.46	-9.80	< .001	0.44	< .001	19.38	28.55	29.18	22.34	-0.34	-5.81	< .001	0.40	< .001
Time until release: <NA>	0.16	0.37	0.28	0.45	-0.34	-7.59	< .001	0.12	< .001	0.16	0.37	0.22	0.41	-0.15	-2.20	.036	0.06	.486

Table A22

Average Effects of 1.000 General Linear Models Predicting Post Treatment Adjudications Based on Treatment (Model 1) and Based on Treatment and Matching Covariates (Model 2) Drawing on Randomly Generated Control Group Observation Periods Following the Intent To Treat (ITT) and Protocol Adherence (PA) Approach

		ITT					PA				
Model	Effect	B	SE	t	*p*	% < .05	B	SE	t	*p*	% < .05
Model 1	(Intercept)	0.25	0.03	8.45	< .001	1.00	0.24	0.03	8.27	<.001	1.00
	TP Treatment	-0.09	0.04	-2.53	.027	0.85	-0.10	0.04	-2.68	.017	0.92
Model 2	(Intercept)	0.41	0.23	1.75	.116	0.35	0.32	0.23	1.33	.234	0.10
	TP Treatment	-0.08	0.04	-1.65	.163	0.33	-0.09	0.04	-1.93	.097	0.48
	Age	0.00	0.00	-1.07	.321	0.01	0.00	0.00	-1.25	.247	0.04
	Ethnicity – Black	0.12	0.06	2.11	.096	0.62	0.14	0.06	2.34	.067	0.73
	Ethnicity – Mixed	0.05	0.06	0.77	.454	0.01	0.06	0.06	1.02	.360	0.04
	Ethnicity – White	0.09	0.05	2.04	.127	0.59	0.09	0.05	2.05	.119	0.57
	Ethnicity – Other	-0.10	0.11	-0.99	.359	0.01	-0.09	0.10	-0.99	.363	0.03
	Prison – D-Cat	0.00	0.08	-0.02	.645	0.00	0.00	0.08	0.04	.652	0.00
	Prison – C-Cat	-0.01	0.07	-0.16	.602	0.00	-0.02	0.07	-0.28	.605	0.00
	Prison – B-Cat	0.05	0.08	0.69	.490	0.02	0.07	0.08	0.82	.438	0.02
	Prison – A-Cat	0.19	0.28	0.62	.555	0.00	0.19	0.28	0.63	.548	0.00
	Copas	0.08	0.03	2.47	.019	0.94	0.07	0.03	2.12	.045	0.68
	IEP – Standard	-0.22	0.19	-1.09	.320	0.02	-0.12	0.20	-0.55	.567	0.00
	IEP – Enhanced	-0.34	0.19	-1.78	.106	0.34	-0.24	0.20	-1.16	.287	0.02
	Adjudications (pre)	0.25	0.10	2.47	.018	0.96	0.20	0.08	2.38	.025	0.87
	Casenote balance (pre)	-0.02	0.01	-1.32	.219	0.06	-0.02	0.01	-1.47	.171	0.11
	Self-harm (pre)	0.09	0.06	1.55	.208	0.24	0.10	0.06	1.72	.173	0.29
	IO: Drug offences	0.16	0.07	2.50	.080	0.73	0.15	0.07	2.32	.089	0.68
	IO: Fraud offences	0.30	0.16	1.87	.080	0.44	0.30	0.17	1.82	.085	0.37
	IO: Miscellaneous crimes against society	0.15	0.11	1.42	.211	0.16	0.17	0.12	1.44	.196	0.13
	IO: Possession of weapons	0.27	0.12	2.22	.054	0.74	0.27	0.13	2.20	.053	0.73
	IO: Public order offences	0.17	0.12	1.48	.185	0.13	0.17	0.12	1.50	.175	0.15
	IO: Robbery	0.25	0.11	2.38	.051	0.77	0.21	0.10	2.14	.079	0.65

Table A22

Average Effects of 1.000 General Linear Models Predicting Post Treatment Adjudications Based on Treatment (Model 1) and Based on Treatment and Matching Covariates (Model 2) Drawing on Randomly Generated Control Group Observation Periods Following the Intent To Treat (ITT) and Protocol Adherence (PA) Approach

		ITT					PA				
Model	Effect	B	SE	t	*p*	% < .05	B	SE	t	*p*	% < .05
	IO: Sexual offences	0.57	0.40	1.43	.160	0.01	0.57	0.41	1.39	.173	0.01
	IO: Summary motoring	0.69	0.35	2.00	.051	0.60	0.70	0.34	2.03	.048	0.65
	IO: Summary non-motoring	0.17	0.08	2.14	.105	0.62	0.15	0.09	1.94	.132	0.54
	IO: Theft offences	0.12	0.08	1.57	.205	0.34	0.14	0.08	1.84	.151	0.50
	IO: Unknown	0.39	0.38	1.01	.202	0.40	0.36	0.40	0.73	.210	0.36
	IO: Violence against the person	0.24	0.07	3.55	.024	0.89	0.23	0.07	3.34	.027	0.88
	Time until release	0.00	0.00	-0.91	.405	0.01	0.00	0.00	-0.58	.546	0.00

Note. Estimates and p values are averages of 1.000 Bootstrapp iterations. In addition to average unstandardised estimates, standard errors, t-values and p values we provide the percentage of iterations with $p < .05$ in the table (%<.05) on a scale from 0 to 1. *P tests* are two-tailed. ITT Model 1, $F(1, 4908) = 6.77$, $p = .019$ [%< .05 = 0.87]; ITT Model 2, $F(29, 1790) = 5.09$, $p < .001$ [%< .05 = 0.99]; PA Model 1, $F(1, 4853) = 7.35$, $p = .016$ [%< .05 = 0.93]; ITT Model 2, $F(29, 1742) = 7.65$, $p < .001$ [%< .05 = 0.99].

Supplementary Information B (Study 2): The impact of identity fusion to positive and negative social groups on future optimism and prison behaviour among Twinning Project participants

Sample

Independent from the prison behaviour data for Study 1, a subsample of n = 543 individuals was recruited to take part in a longitudinal survey (Study 2) distributed across 19 prisons, which were selected to represent the regions and categories of prison in the UK. Coaches and prison staff received research training prior to data collection. The research period was defined as between September 2021 and March 2023. All participants enrolled on identified cohorts were eligible to participate. Each participant created a unique identifying code, which was used to match survey responses across timepoints and, later, with their prison behaviour. 52 responses could not be identified based on their unique code. Of the remaining n = 491 surveys, the data of 103 cases was removed prior to further analyses (12 cases were outside of the research period and not eligible, 25 cases did not include confirmation of informed consent, 66 female participants), leading to a final subsample of n = 388 male longitudinal T0 survey responses (T1 n = 283, T2 n = 213). Due to the unique needs of the women’s population and the unbalanced sample it presented for this paper, the results of the women’s data will be written up in a separate paper.

Independent sample proportion tests showed that the group recruited for studies into social bonding experiences was highly similar to the remaining sample in terms of age distribution (p’s > .072) and ethnicity (only the proportion of individuals who declared their ethnicity as “other” was higher in the subsample, 2% vs 0%, Z = 2.94, p = .003). Regarding the sample’s criminal justice background, differences mostly related to prison status: the survey-subsample contained more category B prisoners, and significantly fewer category D, C or A prisoners (p’s < .003), as well as more prisoners housed in the greater London area (13% vs 3%, p < .001), and in privately run institutions (29% vs 12%, p < .001), however there were no significant differences with regard to the months until release (p = .051). Individuals in the subsample had on average spent 7.45 months less in prison (across their lifespan) (t(724,71) = -2.16, p = .032), but there were no differences regarding numbers of convictions or COPAS rate (index reflecting length and intensity of criminal career) (p’ s > .176).

Materials

Prison behavioural indicators were captured with the measures described in SI A. Survey data was collected as part of a larger project and here we report only the variables that were used in the analyses reported for this pre-registered study.

Identity fusion (adjusted version Swann et al., 2009). Participants completed a pictorial identity fusion scale. They read the following text “In the below pictures, you will see two circles. One of the circles represents you, and the other circle represents a group to which you belong. The overlap between the circles represents the degree of closeness between yourself and a group.” Participants where then prompted to select one of the choices (A-E) with reference to the following groups: The Twinning Project, My family, All football fans everywhere, My country, A criminal group (in or outside prison, such as a gang or friends who deal drugs).

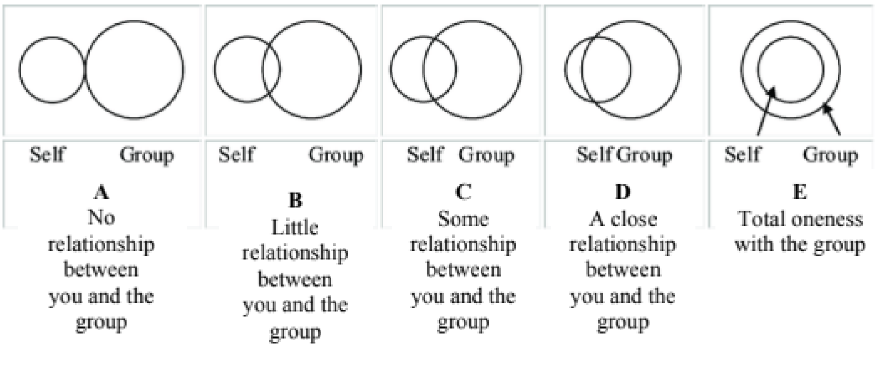

Future optimism. Participants indicated their Future optimism with two items: “What do you think your chances are to have/keep a good job?” and “What do you think your chances are to stay out of trouble with the

law?”. They responded to the items on a scale from 1 = poor, 2 = quite poor, 3 = neither good nor bad, 4 = good, 5 = excellent.

Identification (Postmes et al., 2013). Participants indicated their agreement to the statement “I identify with…” in reference to each of the following groups, using a scale from 1 = Strongly agree, 2 = Agree, 3 = Neither agree nor disagree, 4 = Disagree, 5 = Strongly disagree: The Twinning Project, My family, All football fans everywhere, My country, A criminal group (in or outside prison, such as a gang or friends who deal drugs).

Demographics. Participants indicated their **age** (“How old are you?”) in years. They indicated their **ethnicity** (“How do you describe your ethnicity?”) by selecting one of the following options: Asian, Black, Mixed, North African, White, Prefer not to say. Participants indicated their **level of education** (“What is your education level?”) by selecting from the following options: 1 = without qualifications; 2 = GCSEs or similar; 3 = College / apprenticeship.

Time left to serve. Participants were asked ”How long do you have left to serve?” and indicated their answer in years and months.

Analyses

Deviations from Pre-registration

In line with the pre-registered hypothesis that behaviour would improve over time and the significant treatment effect of TP in Study 1, we additionally explored the pre-post differences in adjudications among a sub-sample of the treatment group which had the potential to show improvements over time, i.e., with a pre-treatment count of adjudications >0. The results are reported with an asterisk in Table B1.

Similarly, and in line with the pre-registered hypothesis that future optimism would improve over time, we additionally explored the pre-post differences in future optimism among a subsample of the treatment group which did not score at ceiling level at the start of the treatment (i.e., with a T0 score of future optimism <5). The results are reported with an asterisk in Table B2.

The planned analyses of the hypothesis that “De-fusion from criminal groups will be moderated by fusion to the Twinning Project, i.e., changes in levels of criminal fusion/ prevalence of criminal fusion will correlate with ‘fusion to the TP’ after the treatment (T2).” was flawed as the hypothesised decrease in fusion to criminal groups was not observed in the sample. To test whether changes in social bonding correlate overall, we conducted additional bivariate correlation analyses for associations between fusion and identification change scores, which are reported in Table B3.

In line with the pre-registered hypothesis that social cohesion experiences correlate with prison behaviour and informed by the exploratory analysis that improvement for adjudications was observed among a sub-sample (as per Table B1), we also explored whether such cases with improved behaviour, i.e., fewer adjudications after vs before the treatment (coded binary 1 = yes, 0 =no) would be associated with the hypothesised increases in social bonding to the intervention programme. The results are reported in Table B6.

Differences over time

We conducted a series of paired samples t-tests to compare levels of prison behaviour indicators in the 2 months before and after treatment (Table B1), as well as indicators of social bonding and future optimism at the treatment start, treatment end and at a 2 months follow-up (Table B2).

Table B1
Paired Sample T-Tests of Prison Behaviour Indicators Among Twinning Project Participants

Outcome variable	Pre		Post		*t*(df)	*p*	Cohen’s *d*
	M	SD	M	SD			
Adjudications	0.13	0.44	0.15	0.55	-1.28 (833)	.101	.04

Adjudications*	1.30	0.70	0.48	1.04	7.23 (80)	< .001	.80
Case note balance	0.66	2.27	0.49	2.58	1.57 (675)	.058	.06
Self-harm	0.01	0.19	0.02	0.24	0.58 (833)	.249	.02

Note. * Among subsample of prisoners with at least 1 adjudication pre-treatment.
p tests are one-tailed and not adjusted for multiple comparisons.
The normality assumption for each paired sample t-test was violated. A sensitivity analysis using the Wilcoxon Signed-Rank Test showed that the results did not change substantively, ensuring robustness despite non-normal data.

Table B2

Paired Sample T-Tests of Social Bonding Indicators Among Twinning Project Participants between the Start (T0), End (T1) and Follow-Up (T2)

Outcome variable	T0		T1		*t*(df)	*p*	Cohen's *d*
	M	*SD*	*M*	*SD*			
Identification TP	3.63	0.96	4.00	0.82	*-5.08 (243)*	*< .001*	.33
Identification Criminals	1.95	1.10	2.04	1.19	*-1.24 (254)*	*.108*	.08
Fusion TP	3.29	1.26	3.74	1.01	*-4.96 (255)*	*< .001*	.31
Fusion Criminals	1.69	1.01	1.86	1.18	*-2.12 (260)*	*.018*	.13
Future optimism Job	4.09	0.85	4.14	0.82	*-1.10 (266)*	*.137*	.07
Future optimism Desistance	4.26	0.82	4.27	0.80	*-0.15 (253)*	*.439*	.01
Future optimism Job*	3.61	0.65	3.91	0.81	*-5.06 (174)*	*< .001*	.38
Future optimism Desistance*	3.66	0.65	4.01	0.79	*-5.46 (139)*	*< .001*	.46
	T0		T2		*t*(df)	*p*	Cohen's *d*
	M	*SD*	*M*	*SD*			
Identification TP	3.65	0.97	3.96	0.77	-4.09 (186)	< .001	.30
Identification Criminals	1.91	1.04	1.94	1.20	-0.39 (198)	.349	.03
Fusion TP	3.30	1.27	3.49	1.09	-1.65 (192)	.050	.12
Fusion Criminals	1.66	0.98	1.77	1.14	-1.24 (199)	.108	.09
Future optimism Job	4.06	0.84	4.23	0.75	-3.01 (204)	.001	.21
Future optimism Desistance	4.36	0.71	4.35	0.77	0.17 (196)	.431	.01
Future optimism Job*	3.62	0.65	4.06	0.77	6.62 (138)	< .001	.56
Future optimism Desistance*	3.75	0.48	4.10	0.80	-4.26 (101)	< .001	.42

Note. *Among subsample of prisoners below maximum levels of optimism (> 5) at treatment start (T0).
p tests are one-tailed and not adjusted for multiple comparisons.
The normality assumption for each paired sample t-test was violated. A sensitivity analysis using the Wilcoxon Signed-Rank Test showed that the results did not change substantively, ensuring robustness despite non-normal data.

Table B3

Pearson Correlations Between Social Bonding and Future optimism Difference Scores

	N	M	SD		2.	3.	4.	5.	6.
1. Identification TP (T0-T1)	244	0.38	1.16	*r*	.39	-.15	.05	.16	.24
				p	<.001	.020	.402	.015	<.001
2. Fusion TP (T0-T1)	256	0.45	1.44	*r*		-.04	.07	.15	.16
				p		.533	.308	.022	.01
3. Identification Criminals (T0-T1)	255	0.09	1.11	*r*			.37	-.10	-.03
				p			<.001	.11	.64
4. Fusion Criminals (T0-T1)	261	0.17	1.29	*r*				-.15	.00
				p				.014	1.000
5. Future optimism job (T0-T1)	267	0.06	0.84	*r*					.34
				p					<.001
6. Future optimism desistance (T0-T1	254	0.01	0.82	*r*					

Note. p tests are two-tailed and not adjusted for multiple comparisons.

Table B4

Descriptive Statistics and Pearson Correlations Between Variables in Study 2

		M	SD	N		2.	3.	4.	5.	6.	7.	8.	9.	10.	11.	12.	13.	14.	15.	16.	17.	18.	19.	20.	21.	22.	23.	24.
1.	Fusion TP T0	3.29	1.29	372	*r*	.06	.47	-.15	.08	.16	.03	.22	-.06	.19	-.08	-.00	.06	-.04	.07	.00	.22	-.19	.10	.16	.03	.08	-.10	.01
					p	.232	<.001	.004	.130	.002	.628	<.001	.312	.002	.197	.945	.365	.426	.336	.951	.002	.006	.158	.027	.649	.122	.075	.869
2.	Fusion crim. T0	1.66	1.03	378	*r*		-.12	.55	-.23	-.28	.07	-.09	.32	-.07	.42	-.26	-.26	-.08	-.01	.31	-.04	.34	-.28	-.34	.01	-.00	-.17	-.06
					p		.027	<.001	<.001	<.001	.225	.177	<.001	.255	<.001	<.001	<.001	.135	.945	<.001	.590	<.001	<.001	<.001	.830	.976	.001	.346
3.	Identification TP T0	3.65	0.95	356	*r*			-.02	.11	.20	-.04	.17	-.02	.15	-.02	.09	.09	.08	.29	-.16	.31	-.14	.23	.26	.07	.04	-.02	.07
					p			.749	.047	<.001	.463	.007	.786	.016	.721	.156	.159	.187	<.001	.025	<.001	.058	.001	<.001	.217	.404	.765	.307
4.	Ident. crim. T0	1.98	1.13	368	*r*				-.23	-.29	.01	-.03	.42	-.10	.53	-.19	-.30	-.03	-.05	.31	.15	.36	-.12	-.18	.08	-.08	-.18	-.05
					p				<.001	<.001	.882	.698	<.001	.892	<.001	.002	<.001	.593	.486	<.001	.034	<.001	.081	.013	.140	.131	<.001	.423
5.	Future op. job T0	4.07	0.85	385	*r*					.49	-.08	-.04	-.04	-.06	-.15	.50	.36	-.02	-.07	-.19	.10	-.08	.48	.27	-.02	.07	.16	-.08
					p					<.001	.162	.524	.523	.332	.019	<.001	<.001	.685	.337	.006	.177	.254	<.001	<.001	.688	.164	.003	.204
6.	Future op. desist T0	4.30	0.81	382	*r*						-.05	.06	-.28	-.00	-.31	.33	.49	-.01	.13	-.22	.05	-.23	.27	.39	-.00	-.02	.20	-.02
					p						.364	.342	<.001	.944	<.001	<.001	<.001	.927	.078	.002	.514	<.001	<.001	<.001	.991	.715	<.001	.746
7.	Adjudications prior	0.15	0.52	337	*r*							-.06	.13	.09	.05	-.10	-.18	.16	-.12	-.01	-.06	.02	.01	-.05	.19	-.06	-.09	.07
					p							.326	.042	.151	.447	.118	.007	.003	.100	.920	.412	.739	.899	.545	<.001	.283	.100	.220
8.	Fusion TP T1	3.73	1.01	264	*r*								-.10	.43	.03	.06	.14	.02	.20	.11	.28	-.02	-.01	.04	.02	-.02	.02	-.01
					p								.121	<.001	.638	.340	.034	.714	.010	.161	<.001	.798	.887	.586	.790	.787	.768	.872
9.	Fusion crim. T1	1.89	1.18	267	*r*									.04	.57	-.18	-.22	-.03	-.04	.38	.06	.39	-.15	-.23	.05	.03	-.17	.05
					p									.572	<.001	.003	<.001	.660	.602	<.001	.417	<.001	.050	.003	.393	.631	.007	.522
10.	Identification TP T1	4.00	0.83	262	*r*										-.12	.11	.17	.01	.08	-.02	.36	-.06	-.01	-.07	.12	.10	.09	.06
					p										.065	.072	.007	.845	.338	.824	<.001	.475	.932	.373	.068	.100	.165	.439
11.	Identification crim. T1	2.03	1.18	264	*r*											-.24	-.34	-.08	-.03	.41	.09	.47	-.21	-.32	.10	-.10	-.14	-.16
					p											<.001	<.001	.23	.716	<.001	.247	<.001	.007	<.001	.109	.116	.027	.025
12.	Future op. job T1	4.14	0.82	270	*r*												.52	.069	-.04	-.32	.12	-.14	.43	.31	-.04	.08	.18	.03
					p												<.001	.28	.656	<.001	.130	.066	<.001	<.001	.535	.179	.004	.726

13.	Future op. desist T1	4.28	0.79	258	*r*	.07	.11	-.24	-.03	-.18	.25	.24	-.12	.13	.21	.06
					p	.283	.160	.002	.679	.019	.001	.002	.058	.038	<.001	.441
14.	Adjudications during	0.15	0.71	337	*r*		.01	.16	.09	.09	.14	.10	.17	-.02	.04	-.07
					p		.889	.028	.217	.230	.049	.202	.002	.746	.512	.267
15.	Fusion TP T2	3.49	1.09	197	*r*			-.18	.31	-.21	.09	.27	-.05	.08	-.01	.15
					p			.012	<.001	.003	.218	<.001	.510	.262	.942	.085
16.	Fusion crim. T2	1.77	1.17	204	*r*				.00	.49	-.27	-.35	.04	-.07	-.12	.03
					p				.957	<.001	<.001	<.001	.553	.340	.087	.727
17.	Identification TP T2	3.98	0.76	201	*r*					-.02	.23	.10	.06	.11	.09	.06
					p					.799	.001	.180	.422	.130	.235	.486
18.	Identification crim. T2	1.94	1.19	206	*r*						-.28	-.49	.25	-.11	-.08	-.11
					p						<.001	<.001	<.001	.134	.272	.188
19.	Future op. job T2	4.23	0.75	2074	*r*							.55	.02	.13	.18	-.08
					p							<.001	.795	.067	.014	.328
20.	Future op. desist T2	4.33	0.79	198	*r*								-.03	.18	.05	-.02
					p								.725	.011	.507	.810
21.	Adjudications post	0.15	0.52	337	*r*									-.04	-.07	-.03
					p									.495	.217	.672
22.	Age	30.20	7.12	387	*r*										.01	.09
					p										.846	.125
23.	Education	2.08	0.68	358	*r*											-.02
					p											.749
24.	Months to release	9.56	25.97	278	*r*											
					p											

Note. *p* tests are two-tailed and not adjusted for multiple comparisons.

Table B5

Linear Regression Analyses Predicting Post-Treatment Case Note Balance and Poisson Regression Analysis Predicting Post-Treatment Adjudications

Variables	Case Note Balance				Adjudications				
	Estimate	SE	95% CI	*p*	Estimate	SE	*Exp (B)*	95% CI	*p*
Intercept	-3.57	2.85	-9.154, 2.020	.211	-.81	1.46	.45	-3.667, 2.052	.580
Fusion Twinning Project (T1)	.15	.21	-.256, .550	.475	.13	.18	1.14	-.231, .484	.488
Fusion Criminals (T1)	-.19	.17	-.531, .148	.269	-.02	.15	.98	-.314, .269	.881
Case note balance (Pre)	.37	.09	.191, .552	<.001	.66	.28	1.94	.117, 1.206	.017
Fusion Twinning Project (T0)	.04	.15	-.267, .340	.814	.13	.16	1.13	-.187, .438	.432
Fusion Criminals (T0)	-.05	.19	-.410, .319	.805	.00	.17	1.00	-.330, .324	.985
Age	.04	.03	-.020, .099	.197	-.04	.04	.96	-.107, .034	.311
Ethnicity – Black	2.55	2.45	-2.243, 7.349	.297	.	.	.	.	.
Ethnicity – Asian	1.92	2.36	-2.714, 6.544	.417	.	.	.	.	.
Ethnicity – Mixed	1.43	2.42	-3.313, 6.173	.555	.	.	.	.	.
Ethnicity – White	1.14	2.37	-3.503, 5.773	.632	.37	.45	1.45	-.510, 1.256	.407
Ethnicity – Other	0	.	.	.	.	.	.	.	.
Education	.44	.22	.005, .867	.048	-.36	.25	.70	-.857, .136	.155
Incentives earned privileges (basic)	-1.34	1.70	-4.681, 1.997	.431	.44	1.13	1.55	-1.780, 2.661	.697
Incentives earned privileges (standard)	-.30	.49	-1.258, .651	.533	.68	.47	1.97	-.237, 1.597	.146
Incentives earned privileges (enhanced)	0	.	.	.	.00	.	1	.	
Prison Security level B	-.79	.75	-2.261, .692	.297	-.56	.79	.57	-2.101, .982	.477
Prison Security level C	-.36	.45	-1.246, .526	.426	-.89	.46	.41	-1.795, .021	.055
Prison Security level D	0	.	.	.	.00	.	1	.	
Time until release (in months)	-.00	.01	-.017, .925	.639	.00	.01	1.00	-.013, .014	.938
Copas score	.37	.28	-.187, .925	.193	-.26	.31	.77	-.861, .340	.396

Note. For the model predicting adjudications, ethnicity was collapsed to a binary indicator due to singularities in the Hessian matrix
p tests are two-tailed.

Table B6

Logistic Regression Analysis Predicting Membership in Group of 'Prisoners With Reduced Number of Adjudications Post Treatment'

						95% C.I. for EXP(B)	
		B	*SE*	*p*	Exp(B)	Lower	Upper
Model 1[a]	Identification with TP (T0-T1)	0.63	0.29	.031	1.88	1.058	3.345
	Constant	-2.99	0.45	< .001	0.05		
Model 2[b]	Identification with TP (T0-T1)	2.15	1.03	.037	8.58	1.139	64.624
	Adjudications (pre)	2.96	1.40	.034	19.33	1.248	299.439
	Case note balance (pre)	0.48	0.35	.164	1.62	.820	3.210
	Self-harm incidents (pre)	-12.87	26232.95	1.000	0.00	.000	.
	Age	0.17	0.10	.106	1.18	.965	1.446
	Incentives earned privileges (basic)			.162			
	Incentives earned privileges (standard)	-4.23	10.77	.694	0.02	.000	21246244.937
	Incentives earned privileges (enhanced)	-10.25	11.47	.371	0.00	.000	204029.939
	Time until release	0.09	0.04	.033	1.09	1.007	1.176
	COPAS	-0.80	0.87	.358	0.45	.082	2.475
	Prison security level D			.107			
	Prison security level C	-4.37	2.35	.064	0.01	.000	1.279
	Prison security level B	-5.38	2.58	.037	0.01	.000	.724
	Constant	-3.38	10.90	.756	0.03		

Note. [a]. $\chi 2$ (1) = 4.61, p = .032 Nagelkerke R^2 = .08, [b]. $\chi 2$ (11) = 51.09, p < .001 Nagelkerke R^2 = .76

p tests are two-tailed

Supplementary Information C (Study 3): Associations between fusion to positive/negative groups and criminal behaviour among formerly incarcerated people

Sample

A sample of N = 250 formerly incarcerated people from the UK and US was recruited via the crowdsourcing platform Prolific. One participant was excluded based on their short completion time. The final sample consisted of N = 249 participants (M_{age} = 43.88, SD_{age} = 11.63). The majority of participants were from the United States (66.3%), and the sample was predominantly male (70.3% male, 27.3% female, 2.4% non-binary/third gender) and white (81.5%) (10.8% Black, 2.8% Asian, 2.4% Mixed, 2% Other, 0.4% prefer not to say). Participants reported the reasons for their imprisonment as crimes related to drugs (20.1%), theft (19.7%), violence against others (17.3%), sexual offences (5.2%), arson or criminal damages (1.2%), and other categories (22.5%), while 14.1 % did not want to disclose the type of crime committed.

Materials

Prisoner status confirmation. At the beginning of the survey participants were asked to confirm "Have you ever been to prison?". Participants selecting "no", were removed from the survey.

Future optimism. We used the same measures as S2, i.e., "What do you think your chances are to have/keep a good job?", "What do you think your chances are to stay out of trouble with the law?".

Desistance time. Participants answered "How much time has passed since your last prison sentence? (in years and months)".

Self-reported deviant behaviour. Participants read the following prompt "Most people break the rules at some time or other" and were asked "Since you were released from prison, how often (if at all) have you… ": … committed a minor traffic offence?, driven drunk?, consumed illegal substances?, been violent to someone?, been involved in any other kind of antisocial behaviour (e.g., noise complaints, vandalism, threatening or harassing others)?, done something fraudulent that could get you in trouble with the law?, stolen something?, committed arson (illegally set fire to something)?, been arrested?, been convicted of a crime?". They then indicated the frequency of the behaviours using the following scale: 1 = never, 2 = once or twice, 3 = a few times, 4 = often, 5 = prefer not to answer. Scores were combined into a single indicator with an internal reliability score (Cronbach's α) = .80.

Pro-criminal attitudes (based on Shields & Simourd, 1991). Participants were asked to "indicate whether you agree or disagree with the following statements" using a scale from 1 = Disagree, 2 = Undecided, 3 = Agree: 1. The police are honest. 2. Judges are honest and kind. 3. The police are crooked.4. The law is rotten to the core. 5. The law only helps a few people. 6. Laws are usually bad. 7. Successful people break the law to get ahead. 8. It's ok to break the law, but don't get caught. 9.People like me must break the law to get ahead." Scores were combined into a single indicator with an internal reliability score (Cronbach's α) = .84.

Group-hobby/leisure activity. Participants were asked whether they "have a favourite hobby or leisure activity that you do with other people or as part of a group?" and selected an answer from the list: 1. A team sport. 2. Art 3. Health & fitness 4.Travelling 5.Video games 6. Music 7.Gardening 8. Volunteering 9. Cooking 10. Other, please specify (Text entry) 11. I don't have one." The subsequent questions were displayed in accordance with the groups they selected.

Time spent with groups. Participants were asked: "How much time you spend with the group or people that represent this group?" (Days per week). They answered using a scale from 1-7 for the following groups: 1. My family 2. My close friends 3. My country 4. A criminal group (in or outside prison, such as a gang or friends who deal drugs). 5. People from my favourite hobby/leisure activity 6. My religious community 7.People from my job/profession 8.Another group that is important to me: (please specify)

Identity fusion (based on Swann et al., 2009). Participants read the following text and were shown the picture below: "In the below pictures, you will see two circles. One of the circles represents you, and the other circle represents a group to which you belong. The overlap between the circles represents the degree of closeness between yourself and a group."

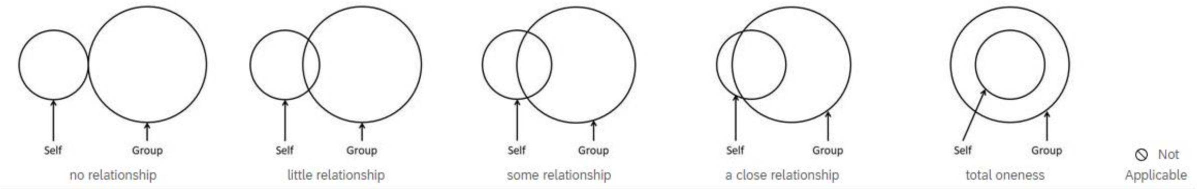

Participants selected one option for each of the following group: 1. My family 2. My close friends 3. A criminal group (in or outside prison, such as a gang or friends who deal drugs). 4. People from my favourite hobby/leisure activity 5. Another group that is important to me: (please specify) 6. My religious community 7. People from my job/profession 8. My country

Perceived values of group. Participants read the following text and question: ”Different groups can stand for different things, and people can associate them with different characteristics. To what extent do you associate “law abiding/ support/ honesty” with the respective groups?”. 1. My family 2. My close friends 3. A criminal group (in or outside prison, such as a gang or friends who deal drugs). 4. People from my favourite hobby/leisure activity 5. Another group that is important to me: (please specify) 6. My religious community 7. People from my job/profession 8. My country
Participants selected an answer for each value and each group using the following scale: 1 = not at all, 2 = A little, 3 = A moderate amount, 4 = a lot.

Demographics. Participants indicated their **age** (“How old are you?”) via a dropdown menu, in years. Participants were asked “How do you describe yourself?” and selected one option to describe their **gender** (male, female, non-binary/third gender, prefer to self-describe, prefer not to say). Participants indicated their e**thnicity** (“How do you describe your ethnicity?”) via a dropdown menu, containing the following options: Asian, Black, Mixed, North African, White, Prefer not to say. Participants indicated their **nationality**, choosing from the options “United States citizen”, “United Kingdom citizen”. Participants indicated their **relationship status** from the following options: single, stable relationship, married, divorced, widowed. Participants indicated their **parenthood status** by indicating the number of children they have, and how many currently live in their household. Participants indicated their current **employment status** (“How would you describe your current employment situation?”) choosing from the following options**:** Unemployed, Part time employed, Full time employment, Self-employed.

Subjective socio-economic status (based on Adler et al., 2000). Participants read the following text and then indicated a number from 1 – 10 on a dropdown menu. “Think of the ladder below as representing **where people stand in society**. At the top of the ladder are the people who are best off – those who have the most money, most education, and the best jobs. At the bottom are the people who are worst off – who have the least money, least education, and the worst jobs or no job. The higher up you are on this ladder, the closer you are to people at the very top and the lower you are, the closer you are to the bottom. Where would you put yourself on the ladder? **<u>Please select the number in the box below</u>** which corresponds to the rung where you think you stand.”

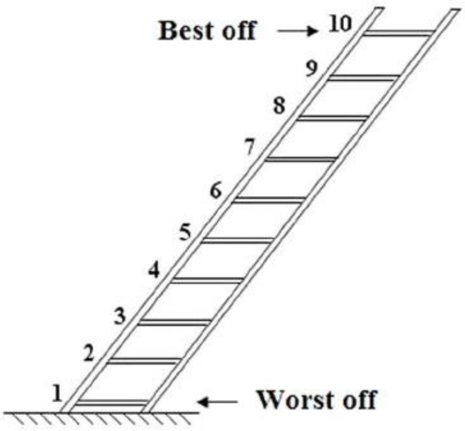

Criminal history background. Participants indicated what type of crime they were imprisoned for: "If you feel comfortable doing so, in your own words, please describe what you served time in prison for (text entry)". They were further asked "How long were you incarcerated?" (in years and months).

Social Desirability Scale-17 (Stoeber, 2001). Participants read the following prompt: "Below you will find a list of statements. Please read each statement carefully and decide if that statement describes you or not. If it describes you, check the word "true"; if not, check the word "false"." *(R) indicates reverse coded items.*
1. I sometimes litter. (R) 2. I always admit my mistakes openly and face the potential negative consequences.3. In traffic I am always polite and considerate of others. 5. I always accept others' opinions, even when they don't agree with my own. 6. I take out my bad moods on others now and then. (R) 7. There has been an occasion when I took advantage of someone else. (R) 8. In conversations I always listen attentively and let others finish their sentences. 9. I never hesitate to help someone in case of emergency. 10. When I have made a promise, I keep it--no ifs, ands or buts. 11. I occasionally speak badly of others behind their back. (R) 12. I would never live off other people. 13. I always stay friendly and courteous with other people, even when I am stressed out. 14. During arguments I always stay objective and matter-of-fact. 15. There has been at least one occasion when I failed to return an item that I borrowed. (R) 16. I always eat a healthy diet. 17. Sometimes I only help because I expect something in return. (R) Scores were combined into a single indicator with an internal reliability score (Cronbach's α) = .79.

Analysis

Given the importance of social, economic and environmental factors for criminal justice outcomes, we first estimated full and partial correlation coefficients between our independent and dependent variables (see Table C1), taking into account relevant (i.e. significant) socio-economic and demographic covariates (see Table C2 for an overview of criminal justice – demographic control correlates). Partial correlations showed that fusion to criminals or friends involved in crime stood out as a consistent, significant correlate of shorter desistance time, stronger pro-criminal attitudes, and more frequent instances of self-reported criminal behaviour, arrest and conviction after release from prison.

Table C1

Descriptive Statistics and Pearson Correlation Coefficients Between Independent and Dependent Variables of Study 3

		M	SD	N		2.	3.	4.	5.	6.	7.	8.	9.	10.	11.	12.	13.	14.	15.
1.	Fusion to family	3.52	1.15	248	*r*	.27	.23	.30	.26	.35	.28	.09	.02 (-.05)	-.12 (-.10)	.03 (.02)	.01 (-.06)	.06 (.00)	.20 (.04)	.22 (.15)
					p	<.001	.002	<.001	<.001	<.001	<.001	.176	.820 (.486)	.067 (.111)	.598 (.779)	.824 (.385)	.369 (.964)	.002 (.577)	<.001 (.022)
2.	Fusion to friends	3.28	0.94	241	*r*		.36	.24	.33	.34	.28	.15	-.01 (.00)	.06 (.07)	.05 (.07)	.02 (-.02)	.07 (.03)	.12 (-.01)	.05 (-.01)
					p		<.001	.001	<.001	<.001	<.001	.031	.913 (.976)	.377 (.278)	.436 (.296)	.818 (.801)	.289 (.689)	.070 (.839)	.467 (.855)
3.	Fusion to people from hobby group	2.89	0.93	182	*r*			.31	.40	.19	.26	.19	.02 (.01)	.10 (.10)	.13 (.12)	.15 (.06)	.09 (.01)	-.02 (-.21)	.02 (-.06)
					p			<.001	<.001	.050	<.001	.014	.807 (.857)	.168 (.164)	.089 (.123)	.048 (.468)	.229 (.913)	.808 (.005)	.790 (.447)
4.	Fusion to religious community	1.81	1.12	180	*r*				.41	.24	.28	.24	-.07 (-.12)	-.11 (-.09)	.07 (.05)	.20 (.12)	.23 (.16)	.03 (-.10)	.06 (-.03)
					p				<.001	.008	<.001	.002	.351 (.131)	.127 (.241)	.359 (.502)	.009 (.109)	.002 (.034)	.696 (.188)	.452 (.706)
5.	Fusion to job/profession	2.40	1.02	211	*r*					.31	.39	.27	.09 (.02)	-.07 (-.03)	.04 (.07)	.14 (.11)	.17 (.13)	.28 (.08)	.05 (-.08)
					p					<.001	<.001	<.001	.212 (.778)	.314 (.706)	.565 (.339)	.039 (.120)	.015 (.065)	<.001 (.231)	.482 (231)
6.	Fusion to other significant group	2.24	1.34	132	*r*						.25	.25	.06 (-.02)	-.05 (.00)	-.01 (.02)	.01 (-.00)	.06 (.04)	.14 (.04)	.06 (-.02)
					p						.004	.005	.532 (.810)	.606 (.962)	.923 (.810)	.884 (.990)	.479 (.672)	.113 (.688)	.493 (.806)
7.	Fusion to country	2.62	1.12	242	*r*							.15	.14 (.05)	-.24 (-.19)	.07 (.09)	.07 (.01)	.08 (.02)	.28 (.13)	.14 (.03)
					p							.033	.035 (.436)	<.001 (.003)	.296 (.180)	.312 (.842)	.205 (.711)	<.001 (.045)	.035 (.630)
8.	Fusion to criminal group	1.80	1.07	242	*r*								-.16 (.01)	.30 (.24)	.45 (.41)	.26 (.24)	.31 (.28)	-.06 (-.09)	-.28 (-.22)
					p								.023 (.938)	<.001 (<.001)	<.001 (<.001)	<.001 (<.001)	<.001 (<.001)	.425 (.220)	<.001 (.001)
9.	Desistance time	10.89	9.00	212	*r*									-.11	.09	.07	.00	.21	.12
					p									.074	.181	.292	.964	<.001	.053
10.	Pro-criminal attitudes	9.80	4.50	249	*r*										.27	.09	.13	-.21	-.33
					p										<.001	.160	.042	<.001	<.001
11.	Post prison criminal behaviour	0.63	0.55	249	*r*											.52	.45	-.04	-.28
					p											<.001	<.001	.487	<.001
12.	Post prison arrest	0.46	0.80	429	*r*												.76	.04	-.14
					p												<.001	.521	.026

13. Post prison conviction	0.37	.070	248	*r*	.01	-.09
				p	.896	.173
14. Future optimism employment	3.26	1.32	249	*r*		.40
				p		<.001
15. Future optimism desistance	4.14	0.91	249	*r*		
				p		

Note. Partial correlations are presented in parentheses: 9. Desistance from crime (age, US, n children, relationship: yes); 10. Pro-criminal attitudes (age, SES); 11. Post prison criminal behaviour (age, relationship: yes, social desirability); 12. Post prison arrest frequency (relationship: yes, SES); 13. Post prison reconviction (SES); 14. Future optimism employment (relationship: yes, employment: yes, SES, social desirability) ; Future optimism desistance (age, SES, social desirability).
p tests are two-tailed and not adjusted for multiple comparisons.

Table C2

Descriptive Statistics and Pearson Correlation Coefficients Between Dependent Variables and Criminal Justice Relevant Control Variables of Study 3

	M	SD	N		2.	3.	4.	5.	6.	7.	8.	9.	10.	11.	12.	13.	14.	15.	16.
1. Desistance time	10.89	9.00	249	*r*	-.11	.09	.07	.00	.21	.12	.49	.10	-.20	.16	.14	-.06	.07	-.09	.07
				p	.074	.181	.292	.964	<.001	.053	<.001	.135	.002	.012	.023	.343	.293	.143	.284
2. Pro-criminal attitudes	9.80	4.50	249	*r*		.27	.09	.13	-.21	-.34	-.34	-.02	.11	.03	-.08	.03	-.18	-.09	-.05
				p		<.001	.160	.042	<.001	<.001	<.001	.738	.095	.619	.221	.608	.004	.147	.449
3. Criminal behaviour	0.63	0.55	249	*r*			.52	.45	-.04	-.28	-.19	.05	.09	.20	.01	.08	.06	-.04	-.31
				p			<.001	<.001	.487	<.001	.003	.475	.182	.001	.941	.191	.325	.540	<.001
4. Re-arrest	0.46	0.80	249	*r*				.76	.04	-.14	-.09	.09	.01	.14	-.04	.00	.15	-.05	-.05
				p				<.001	.521	.026	.137	.174	.887	.023	.515	.995	.020	.468	.392
5. Re-conviction	0.37	.070	249	*r*					.01	-.09	-.12	.07	.10	.12	.00	.01	.15	-.01	-.00
				p					.896	.173	.059	.278	.138	.071	.975	.887	.015	.860	.975
6. Future optimism job	3.26	1.32	249	*r*						.40	.12	.03	-.01	.20	.06	.31	.49	-.03	.14
				p						<.001	.067	.598	.832	.002	.336	<.001	<.001	.660	.033
7. Future optimism desistance	4.14	0.91	249	*r*							.25	-.02	-.09	.02	.09	.01	.26	.07	.30
				p							<.001	.773	.181	.726	.149	.899	<.001	.296	<.001
8. Age	43.88	11.63	249	*r*								-.03	-.04	.00	.28	-.14	.15	.10	.13
				p								.558	.530	.975	<.001	.027	.019	.120	.042
9. Gender	0.70	0.46	249	*r*									-.20	.02	-.15	.01	.04	.18	.07
				p									.002	.774	.020	.855	.554	.005	.310
10. Nationality	0.67	0.47	247	*r*										-.06	-.04	.03	.02	.17	-.11
				p										.345	.534	.671	.726	.010	.090
11. Relationship	0.51	0.50	249	*r*											.19	.16	.27	-.05	.02
				p											.003	.012	<.001	.400	.809

12.	Number of children	2.37	1.47	249	*r*	-.08	.09	.10	.02
					p	-.081	.090	.103	.019
13.	Employment	0.71	0.45	249	*r*		.22	.03	.02
					p		<.001	.654	.728
14.	Socioeconomic status	4.22	1.65	249	*r*			-.02	.15
					p			.689	.017
15.	Time spent in prison (months)	30.89	41.54	246	*r*				-.06
					p				-.058
16.	Social desirability	8.98	3.71	249	*r*				
					p				

Note. *Gender* 1 = male, Nationality 1 = United States, Relationship 1 = married or in stable relationship, Employment 1 = employed (part time, full time or self-employed).
p tests are two-tailed and not adjusted for multiple comparisons.

We further examined the relative impact of fusion for desistance outcomes. We conducted multiple linear regression analyses to predict dependent variables based on fusion to relevant target groups (i.e., significant correlates identified in Table C1), and taking into account demographic controls. Results showed that fusion to criminals and country both significantly predicted stronger and weaker pro-criminal attitudes respectively, whereas only fusion to criminals, significantly predicted self-reported criminal behaviour, rearrest and reconviction after prison release (Table C3).

Table C3

Multiple Linear Regression Predicting Pro-Criminal Attitudes, Self-Reported Criminal Behaviour, Frequency Of Re-Arrest After Release From Prison, and Frequency of Reconviction After Release From Prisons

Variables	Pro-criminal attitudes					Criminal behaviour					Re-arrest					Reconviction				
	B	robust SE	95% CI		*p*	B	robust SE	95% CI		*p*	B	robust SE	95% CI		*p*	B	robust SE	95% CI		*p*
Fusion criminals	1.23	.27	.694	1.767	<.001	.27	.06	.102	.322	<.001	.24	.07	.092	.388	.002	.22	.07	.091	.351	.001
Fusion country	-.93	.30	-1.526	-.337	.002	-	-	-	-	-	-	-	-	-	-	-	-	-	-	-
Fusion hobby/leisure group	-	-	-	-	-	.03	.06	-.073	.128	.591	-	-	-	-	-	-	-	-	-	-
Fusion religious community	-	-	-	-	-	-	-	-	-	-	.06	.07	-.082	.205	.396	.09	.07	-.048	.224	.204
Age	-.09	.03	-.143	-.035	.001	.00	.00	-.013	.006	.450	-.01	.01	-.018	.004	.242	-.01	.00	-.018	.001	.088
Gender	.34	.67	-.990	1.666	.616	.09	.10	-.111	.279	.395	.18	.12	-.052	.415	.127	.14	.12	-.097	.375	.246
Nationality	.93	.66	-.382	2.240	.164	.02	.09	-.201	.142	.736	.05	.14	-.231	.323	.742	.20	.10	-.006	.404	.057
Relationship	.66	.60	-.524	1.845	.273	.21	.09	.091	.430	.003	.13	.13	-.129	.395	.318	.12	.12	-.115	.363	.308
Children	.28	.21	-.130	.687	.180	.05	.03	-.046	.069	.694	.00	.04	-.079	.072	.924	.00	.04	-.071	.073	.971
Employment	.47	.67	-.845	1.795	.479	.12	.10	-.234	.159	.708	-.15	.15	-.441	.147	.325	-.08	.15	-.376	.221	.609
SES	-.32	.21	-.731	.090	.125	.03	.03	-.032	.081	.396	.08	.05	-.008	.174	.073	.05	.04	-.035	.139	.236
Social desirability	.06	.08	-.100	.211	.483	-.06	.01	-.054	-.006	.013	.01	.02	-.027	.039	.710	.02	.01	-.014	.043	.316
Model summary	$F(10,194) = 6.39$, $p < .001$, $R^2 = .25$					$F(10,131) = 5.06$, $p < .001$, $R^2 = .28$					$F(10,155) = 3.75$, $p < .001$, $R^2 = .20$					$F(10,154) = 4.71$, $p < .001$, $R^2 = .23$				

Note. Gender 1 = male, Nationality 1 = United States, Relationship 1 = married or in stable relationship, Employment 1 = employed (part time, full time or self-employed).
p tests are two-tailed. The assumption of homoscedasticity was tested and found to be violated. Robust standard errors were used to obtain reliable inference (Cameron & Miller, 2015).

Testing the pre-registered hypotheses that future optimism toward having a good job and stay out of trouble with the law mediated the link between fusion and criminal justice outcomes, we entered both variables in the abovementioned models (See Tables C4 - C6). Only the effect of future optimism about desistance on pro-criminal attitudes was significant, and, testing for mediation effects using the PROCESS macro (Hayes, 2017) (Model 4) in SPSS, we found a significant indirect effect from fusion to criminals on criminal attitudes via desistance related future optimism (Table C4).

Table C4
Multiple Linear Regression Analyses Predicting Pro-Criminal Attitudes based on Fusion, Future Optimism and Control Variables (Model 1) and Testing for Possible Indirect Effects of Fusion via Future Optimism (Mediation analyses)

Variables	Future Optimism: Desistance					Pro-Criminal Attitudes				
	Estimate	Robust SE	95% CI		*p*	Estimate	Robust SE	95% CI		*p*
Fusion criminals	-.17	.06	-.294	-.039	.011	1.05	.27	.509	1.592	<.001
Fusion country	.04	.06	-.071	.156	.464	-.85	.30	-1.438	-.261	.005
Age	.01	.01	-.002	.020	.104	-.08	.03	-.133	-.027	.004
Gender	-.08	.14	-.351	.189	.554	.27	.67	-1.054	1.600	.685
Nationality	-.10	.12	-.346	.140	.403	.81	.65	-.475	2.099	.215
Relationship	-.15	.12	-.387	.093	.228	.57	.59	-.597	1.741	.335
Children	-.01	.04	-.086	.085	.984	.29	.21	-.117	.699	.161
Employment	-.32	.15	-.607	-.026	.033	.48	.71	-.921	1.889	.497
SES	.04	.04	-.032	.120	.255	-.15	.23	-.594	.302	.521
Social desirability	.04	.02	.005	.072	.024	.10	.08	-.052	.246	.201
Future optimism: Job	.28	.06	.151	.402	<.001	-.21	.32	-.839	.414	.505
Future optimism: Desistance	-	-	-	-	-	-.86	.36	-1.574	-.152	.018
Model summary	$F(11,193) = 8.67, p < .001, R^2 = .33$					$F(12,192) = 6.20, p < .001, R^2 = .28$				
Mediation analyses										
Indirect effect of fusion criminals via future optimism: desistance						.14	.08	.018	.334	

Total effect of fusion criminals via Future optimism: Desistance	1.19	.27	.667	1.721	<.001
Indirect effect of fusion country via future optimism: desistance	-.04	.05	-.146	.063	
Total effect of fusion country via future optimism: desistance	-.89	.30	-1.481	-2.901	.004
Total effect model summary	$F(11, 193) = 6.12, p < .001, R^2 = .26$				

Note. p tests are two-tailed. *Gender* 1 = male, Nationality 1 = United States, Relationship 1 = married or in stable relationship, Employment 1 = employed (part time, full time or self-employed). The assumption of homoscedasticity was tested and found to be violated. Robust standard errors were used to obtain reliable inference (Cameron & Miller, 2015). Indirect effects of mediation models were estimated using Hayes' (2022) PROCESS macro (model 4), which uses a bootstrap method and does not provide p values. Statistical significance is derived from confidence intervals.

Table C5

Multiple Linear Regression Analyses Predicting Criminal Behaviour Based on Fusion, Future Optimism and Control Variables (Model 1)

Variables	Model 1				
	Estimate	Robust SE	95% CI		*p*
Fusion criminals	.20	.06	.092	.314	<.001
Fusion leisure/hobby group	.02	.05	-.082	.131	.655
Age	.00	.00	-.012	.007	.561
Gender	.08	.10	-.124	.278	.451
Nationality	-.04	.09	-.215	.128	.619
Relationship	.25	.09	.075	.418	.005
Children	.01	.03	-.047	.071	.682
Employment	-.04	.10	-.234	.152	.675
SES	.04	.03	-.022	.097	.214
Social desirability	-.03	.01	-.051	-.004	.021
Future optimism: Job	.00	.04	-.083	.075	.921
Future optimism: Desistance	-.08	.07	-.210	.047	.211
Model 1 summary	$F(12, 146) = 5.56$, p <.001 $R^2 = .31$				

Note. p tests are two-tailed. *Gender* 1 = male, Nationality 1 = United States, Relationship 1 = married or in stable relationship, Employment 1 = employed (part time, full time or self-employed). The assumption of homoscedasticity was tested and found to be violated. Robust standard errors were used to obtain reliable inference (Cameron & Miller, 2015).

Table C6

Multiple Linear Regression Analyses Predicting Re-Arrest Based on Fusion, Future Optimism and Control Variables (Model 1)

Variables	Model 1				
	Estimate	Robust SE	95% CI		*p*
Fusion criminals	.22	.08	.070	.375	.005
Fusion leisure/hobby group	.07	.08	-.080	.218	.364
Age	-.01	.01	-.017	.006	.340
Gender	.17	.12	-.063	.412	.148
Nationality	.01	.14	-.269	.299	.918
Relationship	.11	.13	-.147	.370	.397
Children	.00	.04	-.074	.077	.963
Employment	-.17	.15	-.478	.133	.268
SES	.10	.05	.002	.205	.045
Social desirability	.01	.02	-.020	.044	.468
Future optimism: Job	-.01	.06	-.118	.102	.891
Future optimism: Desistance	-.12	.10	-.313	.082	.249
Model 1 summary	$F(12, 153) = 3.38$, $p <.001$, $R^2 = .21$				

Note. p tests are two-tailed. *Gender* 1 = male, Nationality 1 = United States, Relationship 1 = married or in stable relationship, Employment 1 = employed (part time, full time or self-employed).
The assumption of homoscedasticity was tested and found to be violated. Robust standard errors were used to obtain reliable inference (Cameron & Miller, 2015).

Table C7
Multiple Linear Regression Analyses Predicting Reconviction Based on Fusion, Future Optimism and Control Variables (Model 1)

Variables	Model 1				
	Estimate	Robust SE	95% CI		*p*
Fusion criminals	.22	.07	.081	.364	.002
Fusion leisure/hobby group	.08	.07	-.054	.223	.230
Age	-.01	.00	-.018	.002	.107
Gender	.14	.12	-.099	.378	.251
Nationality	.19	.11	-.021	.401	.077
Relationship	.13	.13	-.123	.373	.320
Children	.00	.04	-.068	.078	.897
Employment	-.05	.15	-.337	.238	.734
SES	.07	.05	-.027	.167	.154
Social desirability	.02	.01	-.013	.044	.288
Future optimism: Job	-.05	.06	-.169	.064	.376
Future optimism: Desistance	.00	.08	-.159	.165	.970
Model 1 summary	$F(12, 152) = 4.00$, $p < .001$, $R^2 = .24$				

Note. p tests are two-tailed. *Gender* 1 = male, Nationality 1 = United States, Relationship 1 = married or in stable relationship, Employment 1 = employed (part time, full time or self-employed).
The assumption of homoscedasticity was tested and found to be violated. Robust standard errors were used to obtain reliable inference (Cameron & Miller, 2015).

We also tested whether the effects of fusion are dependent on the values associated, or time spent, with the target group. While there were no significant interactions between time spent and fusion to criminals and associated values and fusion to criminals, we found a significant interaction between fusion to one's country and the associated value support. Specifically, there was a significant negative moderation effect of perceived support, on the relationship between fusion and pro-criminal attitudes (Table C8). The visualisation of this effect (Figure C1) shows that criminal attitudes are highest among those who do not associate their country with 'support' and who score lower on fusion.

Table C8
Multiple Linear Regression Analyses Predicting Pro-Criminal Attitudes Based on Fusion to Groups, Time Spent With Groups, Values Associated With Groups and Their Respective Interaction Terms

Variable	B	Robust SE	95% CI		*p*
Fusion criminals	-.92	.92	-2.743	.896	.318
Time spent with criminals	.40	.80	-1.184	1.986	.618
Criminal group value: law-abiding	.52	.74	-.946	1.987	.484
Criminal group value: honest	-.74	.85	-2.422	.945	.388
Criminal group value: support	-.34	1.08	-2.466	1.795	.756

Fusion criminals * time spent with criminals	.08	.28	-.485	.637	.790
Fusion criminals * criminals value law-abiding	.56	.44	-.311	1.440	.205
Fusion criminals * criminals value honest	.15	.56	-.948	1.251	.786
Fusion criminals * criminals value support	-.25	.31	-.856	.361	.423
Fusion to country	-1.12	1.04	-3.175	.937	.284
Country group value: Law-abiding	.49	.70	-.894	1.877	.485
Country group value: honest	.63	1.23	-1.802	3.056	.611
Country group value: support	-4.07	1.31	-6.652	-1.480	.002
Fusion Country * Country value law-abiding	-.22	.39	-1.000	.553	.570
Fusion Country * Country value honest	-.38	.27	-.906	.148	.158
Fusion Country * Country value support	1.10	.45	.212	1.998	.016
Age	-.09	.03	-.146	-.036	.001
Gender	-.35	.68	-1.697	1.001	.611
Nationality	.85	.63	-.399	2.097	.181
Relationship	.72	.63	-.517	1.958	.252
Children	.39	.22	-.036	.813	.073
Employment	.89	.71	-.501	2.283	.208
SES	-.36	.21	-.775	.055	.089
Social desirability	.09	.08	-.059	.247	.227
Model summary	$F(24,176) = 4.53$, $p < .001$, $R^2 = .38$				

Note. Gender 1 = male, Nationality 1 = United States, Relationship 1 = married or in stable relationship, Employment 1 = employed (part time, full time or self-employed).

Figure C1

Conditional effects of fusion to country on pro-criminal attitudes at +/- 1SD of perceived group value ‘support’

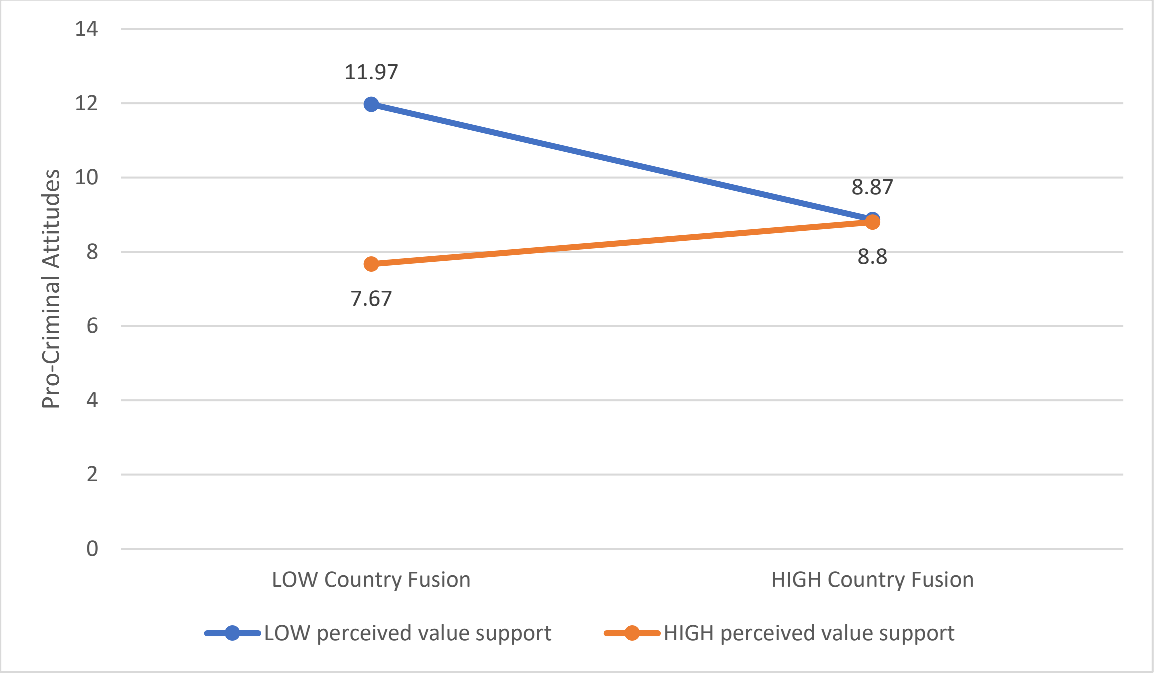

Note. N = 240. Analysis is based on Haye’s (2022) PROCESS macro (Model 1), predicting pro-criminal attitudes based on fusion to country, perceived country value support, their interaction term and socio-demographic controls.

Supplementary Information D (Study 4): Willingness to hire formerly incarcerated people based on prison education & shared interests among the receiving community

Sample

A sample of n = 282 British citizens, both soccer fans and non-fans, was recruited via the crowdsourcing platform Prolific. Due to a technical issue, 2 additional participants completed the survey but were not tracked as such by prolific. 48 participants did not pass an attention check and were excluded from the analyses. The final sample consisted of n = 234 participants (M_{age} = 44.52, SD_{age} 14.05), the majority of whom were male (65.4%; 34.2% female; 0.4% non-binary/third gender) and white (90.2%; 5.1% Asian, 2.1% Black, 2.1% Mixed, 0.4% Prefer not to say). The sample was fairly evenly split into soccer fans (46.6%), and those who did not consider themselves fans (53.4%).

Materials

Prisoner background information [within participant conditions]

- Introduction text

  We are going to show you three stories, each about a different person who has been to prison. Please read the content carefully, as you will be asked to recall some of the basic information later in the survey. After seeing each story, you will be asked to rate the person on different employment criteria. There are no right or wrong answers, we are simply interested in your honest opinion and the impression you formed.

- Twinning Project condition

  Tom is 25 and has just finished a 22 month sentence for a non-violent crime. Like most people in prison he worked during his sentence and attended several educational programmes. He also decided to apply for the Twinning Project. Twinning Project is a partnership between the prison service and professional football clubs. On the programme, professional coaches and football club staff, supported by prison PE officers co-deliver accredited coaching and employability-based qualifications to prisoners to better prepare them for life after their release and can increase their chances of employment. Courses are completed in groups and combine class work and practical skills for 3-12 weeks. Tom successfully completed the project and received the accredited FA Level 1 coaching qualification (Introduction to Coaching Football).

- Vocational programme-condition

  Jamie is 26 and has been released after a 21 month sentence for a non-violent crime. Like most people in prison he worked during his sentence and attended several educational programmes. While in prison he chose to apply for the Professional Gardening course. Professional Gardening courses are provided by the Horticultural Correspondence College and lead to Royal Horticultural Society (RHS) qualifications, which are sought after by employers and can improve someone's chances of employment. In both theoretical and practical sessions, participants can learn about the structure and function of different plants, their nutrition and growing systems and the management of plant health (including dealing with pets, diseases and disorders). The RHS level 2 programme takes the equivalent of 3 - 5 weeks. Jamie successfully completed the course and received the Certificate in Principles of Plant Growth and Development.

- No programme-condition

  Dan is 24 years old and has just finished a 21 month sentence for a non-violent crime. Like most people in prison he worked during his sentence and attended several educational programmes. Dan did not apply for any specialist programmes and did not complete an educational programme during this time. He left prison without receiving any qualifications.

At the end of the survey, participants completed an attention check where they were asked to recall which person did which programme.

Willingness to hire (based on Buhrmester et al. (under review)). Participants were presented with the following scenario and question: “Imagine you are a hiring manager at a building supply store. When reviewing a list of job applicants for an open salesperson position, [Jamie/Tom/Dan] is one of the qualified job candidates. To what extent would you be willing to hire [Jamie/Tom/Dan] ?” Participants selected one of the following options: 1 = definitely unwilling, 2 = Mostly unwilling, 3 = Somewhat unwilling, 4 = Somewhat willing, 5 = Mostly willing, 6 = definitely willing.

Perceived future chances of the applicant. Participants were asked two questions: “How would you rate [Jamie/Tom/Dan]’s …chances to have a good job? …chances to stay out of trouble with the law?” They answered the question choosing one of the following options: 1 = poor, 2 = quite poor, 3 = neither good or bad, 4 = good, 5 = excellent.

Perceived transferrable skills gained in prison programme. Participants read the following question: “Based on what you just read about [Jamie/Tom/Dan] and his prison education, to what extent do you think he has the following skills?” 1. Problem-solving 2. Leadership 3. Teamwork 4. Communication 5. Listening 6. Creativity 7. Attention to detail 8. Relationship building. For each skill participants selected one of the following options: 1 = not at all, 2 = a little, 3 = a moderate amount, 4 = a lot. We estimate mean scores reflecting average levels of perceived transferrable skills for each candidate (TP α = .87, gardening α = 85, no programme α = .91)

Soccer fandom. Participants indicated their status as a soccer fan with one binary question (“Do you consider yourself a football fan? Yes, No”).

Identity fusion (Gómez et al., 2011). Participants indicated to what extent they agree or disagree with the following statements from the verbal fusion scale on a scale from 1 = strongly disagree to 7 = strongly agree. 1. I am one with other fans of my team. 2. I have a deep emotional bond with other fans of my team. 3. I am strong because of other fans of my team. 4. I make other fans of my team strong. Scores were combined into a single indicator with an internal reliability score (Cronbach’s α) = .93.

Interpersonal contact with formerly incarcerated persons (based on Hirschfield & Piquero, 2010). Participants were asked “How many people have you known personally or professionally who have been to prison?” and indicated their response on a scale from 1 = none, 2 = a few, 3 = several, 4 = many.

Political ideology (based on Klingemann, 1997). Participants read the following prompt “In political matters, people talk of ‘the left’ and ‘the right’ “ and then placed themselves on an 11-point scale (0 = left, 1, 2, 3, 4, 5 = center, 6, 7, 8, 9, 10 = right) in response to the following items: “How would you place your views on this scale in general? How would you place your views on this scale when you think about social issues? How would you place your views on this scale when you think about economic issues?”. Scores were combined into a single indicator with an internal reliability score (Cronbach’s α) = .95.

Open-mindedness/ Implicit Person Theory Measure (Levy et al., 1998). Participants indicated to what extent they agreed or disagreed with the following statements on a scale from 1 = strongly disagree to 6 = strongly agree. “1. The kind of person someone is, is something very basic about them and it can’t be changed very much. 2. People can do things differently, but the important parts of who they are can’t really be changed. 3. Everyone, no matter who they are, can significantly change their basic characteristics. 4. As much as I hate to admit it, you can’t teach an old dog new tricks. People can’t really change their deepest attributes. 5. People can always substantially change the kind of person they are. 6. Everyone is a certain kind of person, and there is not much that can be done to really change that. 7.No matter what kind of person someone is, they can always change very much. 8. All people can change even their most basic qualities.” Scores were combined into a single indicator with an internal reliability score (Cronbach’s α) = .93.

Criminal victimisation. Participants were asked: “Have you or a family member ever been a victim of crime?” (1 = Yes, 0 = No)

Hiring experience. Participants indicated their hiring experience with one item: “How many hiring decisions do you estimate you have been involved in?” None, 1-10, 11-20, 21-30, 31-40, 41-50, more than 50.
Additionally participants completed questions on social desirability and socioeconomic status as described in S3 (except for the question about parenting roles, which was not deemed relevant).

Procedure

The study used a 2 (fan vs non-fan) x 3 (programme type: Twinning Project, gardening, no programme) mixed model design. Following the invitation link, participants read the information sheet and provided informed consent. Then participants were introduced to the within-participant manipulation and the three programme types. After reading each vignette, participants completed the relevant measures of perceived future chances, willingness to hire, and perceived transferrable skills. The order in which the three vignettes and respective outcome measures were shown was randomised. Afterwards participants completed the remaining measures capturing their criminal justice experiences, their engagement with the programme contexts, and their demographic characteristics, before receiving a debrief sheet and being automatically returned to the crowdsourcing platform prolific to record their participation.

Analyses

We first conducted repeated measure ANOVAs to test for the effects of programme type and fan status (fan vs non-fan), and their respective interaction, on ratings of employability, skilfulness and future chances in life.

Deviations from pre-registered

A pre-registered regression analysis to test whether perceived skilfulness mediates the relationship between participant group (fan vs non-fan) and perceived employability was not conducted because there was no significant difference for employability between the groups.

Perceived employability.

For the analysis of perceived employability, Mauchly's test indicated a violation of the sphericity assumption, $\chi2(2) = 59.88$, $p < .001$. Since sphericity was violated ($\varepsilon = 0.814$), Greenhouse-Geisser corrected results are reported. There was a significant effect of programme type, $F(1.63, 377.74) = 373.20$, $p < .001$, $\eta2 = 0.617$, as both alumni of the Twinning Project ($M = 4.38$, $SE = 0.06$)- and gardening programme ($M = 4.38$, $SE = 0.06$), were rated as significantly more employable than the person described as not having completed a programme ($M = 2.68$, SE = 0.07) (p's < .001). However, there was no significant difference between fans and non-fans, $F(1, 232) = 1.08$, $p = .301$, and thus, no significant within-between interaction, $F(1.63, 477.74) = 0.78$, $p = .435$.

Perceived skilfulness of alumni.

For the analysis of perceived skilfulness of alumni, Mauchly's test indicated a violation of the sphericity assumption, $\chi2(2) = 42.22$, $p < .001$. Since sphericity was violated ($\varepsilon = 0.857$), Greenhouse-Geisser corrected results are reported. There was a significant effect of programme type, $F(1.71, 397.59) = 602.68$, $p < .001$, $\eta2 = 0.722$, as both alumni of the TP ($M = 3.73$, $SE = 0.05$)- and gardening programme ($M = 3.74$, $SE = 0.04$), were rated as significantly more employable than the case without a completed programme (M = 2.27, SE = 0.05) (p's < .001). Again, there was no significant between subject effect, $F(1, 232) = 1.15$, $p = .285$, and thus, no significant within-between interaction, $F(1.71, 397.59) = 0.194$, $p = .790$.

Perceived chances in life.

The same pattern was evident for the analysis of perceived chances in life. Again, Mauchly's test indicated a violation of the sphericity assumption, $\chi2(2) = 52.93$, $p < .001$. Since sphericity was violated ($\varepsilon = 0.830$), Greenhouse-Geisser corrected results are reported. There was a significant effect of programme type, $F(1.66, 385.14) = 865.08$, $p < .001$, $\eta2 = 0.789$, as both alumni of the TP (M = 2.91, SE = 0.03)- and gardening programme ($M = 2.72$, $SE = 0.03$), were rated as significantly more employable than the case without a completed programme ($M = 1.60$, $SE = 0.03$) (p's < .001). However, there was no significant between subject effect, $F(1, 232) = 2.33$, $p = .128$, and thus, no significant within-between interaction, $F(1.66, 385.14) = 1.90$, $p = .158$.

Covariate analyses.

We also tested repeated measures ANOVAs without the between subject factor (fan status) and with additional relevant covariates (open-mindedness and political ideology for employability; open-mindedness for skilfulness; open-mindedness and SES for future chances) (see Table D1).

Perceived employability.

For the analysis of perceived employability, Mauchly's test indicated a violation of the sphericity assumption, $\chi2(2) = 57.12$ $p < .001$. Since sphericity was violated ($\varepsilon = 0.820$), Greenhouse-Geisser corrected results are reported. The main effect of programme type on employability no longer reached statistical significance, $F(1.64, 378.71) = 2.38$, $p = .105$. There were significant interactions of programme type * open-mindedness $F(1.64, 361.72) = 9.79$, $p < .001$, and programme type * political ideology $F(1.64, 361.72) = 4.22$, $p = .021$. More open orientation was associated with higher ratings of employability for TP ($B = 0.40$, $SE = 0.07$, $t = 5.46$, $p < .001$) and gardening alumni ($B = 0.27$, $SE = 0.08$, $t = 3.52$, $p < .001$), but not for cases without a completed programme ($B = 0.08$, $SE = 0.09$, $t = 0.94$, $p = .347$). In contrast, (more conservative) political ideology significantly predicted lower ratings of employability for the applicant without completed programme ($B = -0.10$, $SE = 0.04$, $t = -2.71$, $p = .007$), but was unrelated to employability in the other two conditions.

Perceived skilfulness of alumni.

For the analysis of perceived skilfulness of alumni, Mauchly's test indicated a violation of the sphericity assumption, $\chi2(2) = 47.83$, $p < .001$. Since sphericity was violated ($\varepsilon = 0.842$), Greenhouse-Geisser corrected results are reported. The abovementioned the main effect of programme type on perceived skilfulness was still significant $F(1.68, 390.89) = 26.32$, $p < .001$, $\eta2 = 0.102$. There was a significant interaction pattern for programme type * open-mindedness $F(1.68, 390.89) = 3.86$, $p = .029$, $\eta2 = 0.016$, where more open-minded participants rated the applicants in the two experimental conditions as more skilful, but not the applicant in the control condition.

Perceived chances in life.

Mauchly's test indicated a violation of the sphericity assumption, $\chi2(2) = 41.70$, $p < .001$. Since sphericity was violated ($\varepsilon = 0.858$), Greenhouse-Geisser corrected results are reported. There was a significant effect of programme type, $F(1.72, 396.29) = 7.63$, $p = .001$, $\eta2 = 0.032$, as both alumni of the Twinning Project (M = 3.73, SE = 0.04)- and gardening programme ($M = 3.74$, $SE = 0.04$), were rated as having significantly better chances in the future than the case without a completed programme ($M = 2.26$, $SE = 0.05$). There was a significant interaction effect between open-mindedness and programme type $F(1.72, 396.29) = 9.31$, $p <.001$, $\eta2 = 0.039$. Parameter estimates showed that open-mindedness was significantly linked with perceived future chances of applicants who completed the football programme ($B = 0.28$, $SE = 0.05$, $t = 5.40$, $p < .001$), and the gardening programme ($B = 0.17$, $SE = 0.05$, $t = 3.48$, $p < .001$), but not the applicant who completed no programme ($B = 0.04$, $SE = 0.06$, $t = 0.66$, $p = .510$), There was also a significant interaction effect between SES and programme type $F(1.72, 396.29) = 3.21$, $p = .049$, $\eta2 = 0.014$, as people who reported higher socioeconomic status were more optimistic towards the alumni of the football programme ($B = 0.06$, $SE = 0.03$, $t = 1.98$, $p = .049$), but not towards the applicants from the other two conditions.

Table D1

Descriptive Statistics and Pearson Correlation Coefficients Between Variables of Study 4

		M	SD	N		2.	3.	4.	5.	6.	7.	8.	9.	10.	11.	12.	13.	14.	15.	16.	17.	18.
1.	Soccer fan	0.47	0.50	234	*r*	.09	.07	.00	.15	.02	.07	.07	.03	.06	-.07	.30	.15	.13	.15	.02	.02	-
					p	.161	.280	.956	.021	.820	.302	.284	.627	.364	.320	<.001	.022	.050	.026	.686	.794	.
2.	TP hiring	4.37	0.98	234	*r*		.67	.31	.60	.41	.12	.73	.52	.24	-.12	-.10	.09	.03	.35	-.06	-.08	.05
					p		<.001	<.001	<.001	<.001	.080	<.001	<.001	<.001	.062	.148	.197	.685	<.001	.390	.252	.597
3.	Garden hiring	4.38	0.97	234	*r*			.32	.41	.53	.16	.46	.67	.23	-.07	-.07	-.02	.01	.23	-.10	-.08	-.04
					p			<.001	<.001	<.001	.016	<.001	<.001	<.001	.304	.321	.803	.883	<.001	.117	.237	.685
4.	control hiring	2.68	1.10	234	*r*				.25	.27	.58	.20	.21	.75	.02	-.13	-.05	.03	.06	-.17	-.04	-.08
					p				<.001	<.001	<.001	.003	.001	<.001	.818	.038	.455	.691	.346	.008	.591	.406
5.	TP skills	2.90	0.49	234	*r*					.67	.26	.65	.46	.25	-.08	-.06	.07	.03	.23	.00	-.05	.11
					p					<.001	<.001	<.001	<.001	<.001	.235	.344	.285	.628	<.001	.968	.481	.270
6.	Garden skills	2.72	0.48	234	*r*						.31	.44	.56	.23	-.11	-.03	-.02	.04	.20	-.09	-.03	.07
					p						<.001	<.001	<.001	<.001	.085	.636	.778	.508	.002	.153	.671	.455
7.	Control skills	1.59	0.48	234	*r*							.08	.13	.57	-.06	.04	-.08	.01	.05	-.05	-.04	-.10
					p							.199	.057	<.001	.358	.712	.254	.877	.431	.471	.503	.292
8.	TP future	3.77	0.71	234	*r*								.64	.30	-.09	.01	.13	.01	.34	-.01	.04	.08
					p								<.001	<.001	.165	.914	.041	.889	<.001	.883	.513	.405
9.	Garden future	3.74	0.64	234	*r*									.30	-.04	-.05	-.02	.04	.22	-.03	.02	.05
					p									<.001	.515	.435	.807	.572	<.001	.620	.821	.581
10.	control future	2.26	0.70	234	*r*										-.04	-.00	-.03	-.03	.04	-.03	-.02	-.02
					p										.503	.781	.673	.619	.519	.643	.786	.879
11.	Age	44.52	14.05	234	*r*											-.08	.10	.23	-.21	.23	.19	.10
					p											.315	.135	<.001	.002	<.001	.004	.324
12.	Gender	0.65	0.48	234	*r*												.02	-.03	.01	.11	.07	-.03
					p												.703	.472	.928	.082	.377	.904

13.	SES	5.71	1.47	234	*r*	.17	.04	.13	-.01	.07
					p	.010	.598	.048	.932	.497
14.	Hiring experience	2.75	1.74	234	*r*		.07	.07	.05	-.06
					p		.274	.288	.423	.555
15.	Open-mindedness	3.39	0.83	234	*r*			-.22	.00	.04
					p			<.001	.980	.681
16.	Political ideology	4.16	2.20	234	*r*				.08	.03
					p				.249	.757
17.	Social desirability	10.14	2.82	109	*r*					.06
					p					.531
18.	Fusion soccer fans[a]	2.95	1.05		*r*					
					p					

Note. Soccer fan 1 = Yes. Gender 1 = male. a Fusion was only measured among soccer fans. *p* tests are two-tailed and not adjusted for multiple comparisons.

Supplementary Information E (Study 5): Fusion and willingness to hire/perceived chances to desist among UK soccer fans

Sample

331 British citizens who reported an interest in soccer were recruited via the crowdsourcing platform Prolific. The sample's interest in soccer was ensured pre-screening for English Premier League fans. Twenty eight participants were excluded because they did not pass an attention check. The final sample consisted of n = 303 participants, (M_{age} = 41.12, SD_{age} 14.25), the majority of whom were male (80.5%; 18.8% female; 0.7% prefer not to say), and white (82.5%; 9.2% Asian, 4% Mixed, 1.7% Black, 1% Other, 0.3% North African, 1.3% Prefer not to say).

Materials

Data was collected as part of a larger project and we report only the variables that were used in the analyses reported for this study.

Willingness to hire, perceived future chances of the applicant, political ideology (Cronbach's α = .95), open-mindedness (Cronbach's α = .92), , interpersonal contact with formerly incarcerated persons, demographics, socio-economic status, hiring experience, victim status, and social desirability (Cronbach's α = .73) were measured as per S4, with the exception of:

Identity fusion (Gomez et al., 2011). Identity fusion was measured with four items: 1. I am one with the applicant. 2. I have a deep emotional bond with the applicant. 3. I am strong because of the applicant. 4. I make the applicant strong. Participants indicated their agreement to the statements with a scale from 1 = strongly disagree to 7 = strongly agree. Scores were combined into a single indicator with an internal reliability score (Cronbach's α) = .84.

Analyses

Deviation from pre-registration

The association between identity fusion (to a job applicant) and willingness to hire/ perceived future chances of the applicant was originally pre-registered to be tested as part of a larger model i.e., as a mediator between experimental triggers of fusion and reintegration support for the applicant. The evaluation of the experimental effects on identity fusion and indirect effects on reintegration support for an formerly incarcerated person are reported as part of another manuscript (REDACTED, under review). The results reported here do not change when tested as part of the original model. Any overlap in reporting of results has been/will be declared.

Correlational analyses

We first estimated bivariate correlation coefficients (Table E1) before testing predictors of willingness to hire and perceived future chances simultaneously via multiple linear regression analyses (Table E2). Results show that fusion to the applicant was the key predictor of both outcome variables, outperforming all other covariates.

Table E1

Descriptive Statistics and Pearson Correlation Coefficients Between Variables of Study 5

	M	SD	N		2.	3.	4.	5.	6.	7.	8.	9.	10.	11.	12.	13.
1. Willingness to hire	4.60	0.98	303	*r*	.49	.35	-.10	.01	-.07	-.07	-.06	-.19	.26	.13	-.07	.11
				p	<.001	<.001	.075	.810	.245	.241	.296	<.001	<.001	.029	.249	.049
2. Perceived chances to desist	3.85	0.69	303	*r*		.30	.00	.05	.03	-.01	.04	-.09	.19	.09	.03	.04
				p		<.001	.997	.355	.553	.922	.479	.127	<.001	.129	.598	.515
3. Identity fusion	2.43	0.84	303	*r*			.03	.09	-.08	-.11	.01	.06	.17	.01	-.12	.14
				p			.566	.110	.171	.052	.812	.272	.004	.866	.046	.016
4. Age	41.12	14.25	303	*r*				-.03	.17	-.09	.45	.19	-.13	.14	.21	.13
				p				.578	.003	.130	<.001	<.001	.021	.018	<.001	.027
5. Gender	0.81	0.40	303	*r*					.01	-.07	.06	.11	-.05	-.00	-.08	.06
				p					.812	.263	.342	.054	.434	.978	.145	.321
6. SES	5.82	1.40	303	*r*						.23	.24	.09	-.06	.01	-.00	.19
				p						<.001	<.001	.117	.326	.811	.978	.001
7. Education	3.68	0.84	303	*r*							.12	-.16	.03	-.11	.05	-.01
				p							.032	.004	.606	.048	.411	.899
8. Hiring experience	1.52	1.61	303	*r*								.07	-.09	.08	.11	.06
				p								.201	.111	.166	.052	.288
9. Political ideology	4.50	2.07	303	*r*									-.26	-.04	-.03	.00
				p									<.001	.449	.569	.976
10. Open mindedness	3.44	0.82	303	*r*										-.02	-.07	.08
				p										.785	.231	.162
11. Contact prisoner	1.57	0.62	303	*r*											.15	-.09
				p											.009	.136
12. Victim of crime	0.59	0.49	303	*r*												-.12
				p												.040

13. Social desirability	10.18	3.17	303	*r*	
				p	

Note. *p* tests are two-tailed and not adjusted for multiple comparisons. Gender 1 = male. Victim *status* 1 = Yes.

Table E2

Multiple Linear Regression Analyses Predicting Willingness to Hire Applicant and Perceived Chances of Applicant to Stay out of Trouble

Variable	Willingness to hire					Perceived chances to desist				
	Estimate	SE	β	95% CI [LL, UL]	*p*	Estimate	SE	β	95% CI [LL, UL]	*p*
Fusion to Applicant	.38	.06	.32	.251, .502	< .001	.24	.05	.29	.149, .335	< .001
Open-mindedness	.18	.07	.15	.047, .308	.008	.12	.05	.14	.020, .214	.018
Political Ideology	-.08	.03	-.16	-.127, -.022	.005	-.03	.02	-.08	-.064, .014	.206
Contact with formerly incarcerated persons	.21	.09	.13	.039, .377	.016	.08	.06	.07	-.045, .207	.207
Victim status	-.02	.11	-.01	-.235, .197	.862	.09	.08	.07	-.070, .251	.266
Age	-.01	.00	-.09	-.015, .002	.131	-.00	.00	-.03	-.008, .005	.690
Gender	-.02	.13	-.01	-.283, .237	.860	.08	.10	.04	-.119, .268	.449
SES	-.00	.04	-.01	-.082, .075	.925	.04	.03	.07	-.023, .093	.240
Education	-.06	.07	-.05	-.192, .067	.346	-.00	.05	-.00	-.100, .093	.940
Hiring Experience	-.00	.04	-.01	-.076, .068	.912	.02	.03	.04	-.038, .069	.571
Social desirability	.02	.02	.08	-.009, .058	.152	-.00	.01	-.02	-.028, .022	.797
Model summary	$F(11,290) = 7.48$, $p < .001$, $R^2 = .22$					$F(11,290) = 4.12$, $p < .001$, $R^2 = .14$				

Note. Gender 1 = male. Victim status 1 = Yes. *p* tests are two-tailed.

Supplementary Information F (Study 6): Fusion and willingness to hire/perceived future chances among US American Football fans

Sample

330 US citizens who reported an interest in American Football were recruited via the crowdsourcing platform Prolific. The sample's interest in the sport was ensured pre-screening for watching American Football on TV and having played American Football in High-school. 36 participants were excluded because they did not pass an attention check crucial to the experimental manipulation. The final sample consisted of N = 294 participants, (M_{age} = 40.92, SD_{age} 12.47), the majority of whom were male (96.9%; 2.4% female, 0.3% non-binary/third gender, 0.3% prefer not to say), and white (76.2%; 7.1 % Black, 7.1% Hispanic, 4.1% Mixed, 3.7% Asian, 1.4% Other, 0.3% prefer not to say). Data was collected as part of a larger project investigating the link between identity fusion and re-integration support formerly incarcerated persons.

Materials

Participants completed the same measures as for S5, with the exception of the measure for education, which was adjusted to the US context. Identity fusion (Cronbach's α = .88), Willingness to hire, perceived future chances of the applicant, political ideology (Cronbach's α = .94), open-mindedness (Cronbach's α = .95), interpersonal contact with formerly incarcerated persons, demographics, socio-economic status, hiring experience, victim status, and social desirability (Cronbach's α = .82).

Education. To indicate their level of education participants chose one of the following options from a dropdown list: 1. No formal qualifications, 2. School qualifications (e.g., High School diploma), 3. Vocational or technical qualifications (e.g., Association degree, certificate), 4.Undergraduate degree or equivalent, 5. Postgraduate degree, 6. Don't know, 7.Prefer not to say.

Analyses

Deviation from pre-registration

The association between identity fusion (to a job applicant) and willingness to hire/ perceived future chances of the applicant was originally pre-registered to be tested as part of a larger model i.e., as a mediator between experimental triggers of fusion and reintegration support for the applicant. The evaluation of the experimental effects on identity fusion and indirect effects on reintegration support for an formerly incarcerated person are reported as part of another manuscript (REDACTED, under review). The results reported here do not change when tested as part of the original model. Any overlap in reporting of results has been/will be declared.

Correlational analysis

We first estimated biavariate correlation coefficients (Table F1) before testing predictors of willingness to hire and perceived future chances simultaneously via multiple linear regression analyses (Table F2). Results show that fusion to the applicant was the key predictor of both outcome variables, outperforming all other covariates.

Table F1

Descriptive Statistics and Pearson Correlation Coefficients Between Variables of Study 6

	M	SD	N		2.	3.	4.	5.	6.	7.	8.	9.	10.	11.	12.	13.
1. Willingness to hire	4.60	1.00	294	*r*	.57	.34	-.16	-.03	-.03	-.09	.11	-.22	.20	.24	.16	-.03
				p	<.001	<.001	.006	.585	.607	.119	.059	<.001	<.001	<.001	.005	.620
2. Perceived chances to desist	3.56	0.84	294	*r*		.37	-.01	.00	.03	-.12	.10	.00	.19	.15	.03	-.04
				p		<.001	.935	.984	.583	.040	.104	.983	.001	.008	.672	.547
3. Identity fusion	2.18	0.89	294	*r*			.06	-.12	.07	-.05	.04	-.06	.04	.25	-.02	.13
				p			.331	.043	.225	.421	.467	.323	.499	<.001	.793	.029
4. Age	40.92	12.47	294	*r*				-.03	.12	.08	.45	.12	-.02	.09	.06	-.08
				p				.556	.035	.158	<.001	.044	.728	.132	.320	.171
5. Gender	0.97	0.17	294	*r*					.05	.04	-.00	-.05	.06	.02	.02	-.01
				p					.366	.527	.970	.426	.275	.715	.724	.918
6. SES	5.37	1.62	294	*r*						.37	.12	.15	.00	-.08	-.09	.12
				p						<.001	.036	.011	.996	.162	.126	.037
7. Education	3.65	1.00	294	*r*							.01	-.08	-.07	-.04	.04	.03
				p							.862	.188	.260	.538	.524	.666
8. Hiring experience	2.09	1.81	294	*r*								.12	.08	.07	.13	-.02
				p								.034	.158	.232	.024	.798
9. Political ideology	4.69	2.61	294	*r*									-.18	-.08	-.13	.05
				p									.002	.171	.023	.418
10. Open-mindedness	3.61	0.92	294	*r*										.08	-.05	.10
				p										.149	.428	.077
11. Contact prisoner	1.86	0.69	294	*r*											.35	-.10
				p											<.001	.098
12. Victim of crime	0.61	0.49	294	*r*												-.17
				p												.003

13. Social desirability	9.20	3.93	294	*r*	
				p	

Note. *p* tests are two-tailed and not adjusted for multiple comparisons. Gender 1 = male. Victim *status* 1 = Yes.

Table F2

Multiple Linear Regression Analyses Predicting Willingness to Hire Applicant and Perceived Chances of Applicant to Stay out of Trouble

Variable	Willingness to hire					Perceived chances to desist				
	Estimate	SE	β	95% CI [LL, UL]	*p*	Estimate	SE	β	95% CI [LL, UL]	*p*
Fusion to Applicant	.35	.06	.31	.227, .464	< .001	.35	.05	.37	.243, .454	< .001
Open-mindedness	.15	.06	.14	.034, .259	.011	.15	.05	.16	.049, .250	.004
Political Ideology	-.06	.02	-.16	-.102, -.021	.003	.01	.02	.04	-.022, .050	.451
Contact with formerly incarcerated persons	.16	.08	.11	-.005, .317	.057	.04	.07	.04	-.101, .186	.559
Victim status	.20	.11	.10	-.029, .420	.087	.02	.10	.01	-.181, .217	.858
Age	-.02	.01	-.26	-.030, -.012	< .001	-.00	.00	-.06	-.012, .004	.303
Gender	-.14	.30	-.03	-.724, .438	.628	.16	.26	.03	-.356, .675	.543
SES	.02	.04	.04	-.047, .091	.530	.03	.03	.06	-.033, .089	.363
Education	-.07	.06	-.07	-.181, .038	.201	-.09	.05	-.10	-.183, .011	.083
Hiring experience	.11	.03	.20	.048, .173	< .001	.04	.03	.08	-.018, .094	.180
Social desirability	-.02	.01	-.06	-.042, .010	.229	-.02	.01	-.10	-.044, .003	.082
Model summary	$F(11,281) = 10.08$, $p < .001$, $R^2 = .28$					$F(11,281) = 6.42$, $p < .001$, $R^2 = .20$				

Note. *p* tests are two-tailed. Gender 1 = male. *Victim status* 1 = Yes.

Supplementary Information G (Study 7): Fusion and willingness to hire/ perceived future chances among US nationals

Sample

333 US citizens were recruited via the crowdsourcing platform Prolific. Given the content of one experimental manipulation referring to a national tragedy occurring in 2005, we pre-screened the sample to include only US nationals who were at least 10 years old at that time, i.e. who were at least 28 years old at the time of data collection. 14 participants were excluded because they did not pass an attention check. The final sample consisted of n = 319 participants, (M_{age} = 42.49, SD_{age} 11.69), the majority of whom were female (57.7%; 41.4% male, 0.6% non-binary, 0.3% prefer not to say), and white (74%; 7.8% Black, 6.3% Hispanic, 5% Asian, 3.4% Mixed, 2.5% Other, 0.9% Prefer not to say). Data was collected as part of a larger project investigating the link between identity fusion and re-integration support for formerly incarcerated people.

Materials

Participants completed the same measures as for S6. Identity fusion (Cronbach's α = .91), Willingness to hire, perceived future chances of the applicant, political ideology (Cronbach's α = .97), open-mindedness (Cronbach's α = .95), criminal victimisation, interpersonal contact with formerly incarcerated persons, demographics, socio-economic status, hiring experience, victim status, and social desirability (Cronbach's α = .82).

Analyses

Deviation from pre-registration

The association between identity fusion (to a job applicant) and willingness to hire/ perceived future chances of the applicant was originally pre-registered to be tested as part of a larger model i.e., as a mediator between experimental triggers of fusion and reintegration support for the applicant. The evaluation of the experimental effects on identity fusion and indirect effects on reintegration support for an formerly incarcerated person are reported as part of another manuscript (REDACTED, under review). The results reported here do not change when tested as part of the original model. Any overlap in reporting of results has been/will be declared.

Correlational analysis

We first estimated bivariate correlation coefficients (Table G1) before testing predictors of willingness to hire and perceived future chances simultaneously via multiple linear regression analyses (Table G2). Results show that fusion to the applicant was the key predictor of both outcome variables, outperforming all other covariates.

Table G1

Descriptive Statistics and Pearson Correlation Coefficients Between Variables of Study 7

	M	SD	N		2.	3.	4.	5.	6.	7.	8.	9.	10.	11.	12.	13.
1. Willingness to hire	4.65	1.03	319	*r*	.65	.30	-.13	-.06	-.13	-.11	-.03	-.20	.28	.22	.11	-.04
				p	<.001	<.001	.021	.318	.017	.057	.579	<.001	<.001	<.001	.045	.502
2. Perceived chances to desist	3.57	.81	319	*r*		.33	-.03	-.04	-.01	-.05	-.01	-.13	.24	.12	-.00	.05
				p		<.001	.654	.505	.881	.366	.821	.018	<.001	.033	.980	.347
3. Identity fusion	2.06	.95	319	*r*			-.06	.15	.09	.03	.12	-.03	.12	.11	-.05	.17
				p			.294	.007	.131	.627	.034	.570	.038	.049	.377	.003
4. Age	42.49	11.69	319	*r*				-.17	.03	.07	.18	.30	-.05	.02	-.06	.11
				p				.003	.568	.238	.001	<.001	.334	.721	.277	.059
5. Gender	.41	.49	319	*r*					.04	.01	.08	.08	.04	.01	-.00	.01
				p					.480	.914	.148	.161	.475	.851	.954	.864
6. SES	4.92	1.64	319	*r*						.38	.15	.04	-.13	-.16	-.14	.12
				p						<.001	.006	.417	.025	.004	.016	.027
7. Education	3.42	1.08	319	*r*							.19	-.12	-.04	-.08	-.09	.04
				p							<.001	.029	.456	.139	.113	.488
8. Hiring experience	1.51	1.69	319	*r*								.09	-.13	.14	.07	.06
				p								.109	.024	.016	.247	.304
9. Political ideology	3.64	2.96	319	*r*									-.18	.06	-.14	.08
				p									<.001	.316	.010	.159
10. Open mindedness	3.46	.95	319	*r*										.16	.10	-.02
				p										.004	.072	.694
11. Contact prisoner	1.79	.69	319	*r*											.22	-.10
				p											<.001	.068
12. Victim of crime	.65	.48	319	*r*												-.13
				p												.020

13.	Social desirability	8.74	3.92	319	*r*
					p

Note. *p* tests are two-tailed and not adjusted for multiple comparisons. Gender 1 = male. Victim *status* 1 = Yes.

Table G2

Multiple Linear Regression Analyses Predicting Willingness to Hire Applicant and Perceived Chances of Applicant to Stay out of Trouble

Variable	Willingness to hire					Perceived chances to desist				
	Estimate	SE	β	95% CI [LL, UL]	*p*	Estimate	SE	β	95% CI [LL, UL]	*p*
Fusion to Applicant	.31	.06	.28	.193, .423	< .001	.26	.05	.30	.165, .350	< .001
Open-mindedness	.20	.06	.18	.086, .313	< .001	.15	.05	.18	.062, .244	.001
Political Ideology	-.05	.02	-.14	-.086, -.009	.015	-.03	.02	-.12	-.063, -.001	.045
Contact with formerly incarcerated persons	.21	.08	.14	.049, .371	.011	.08	.07	.07	-.051, .208	.235
Victim status	.07	.12	.03	-.158, .294	.555	-.05	.09	-.03	-.232, .132	.589
Age	-.01	.01	-.06	-.015, .004	.248	.00	.00	.03	-.006, .010	.639
Gender	-.22	.11	-.10	-.433, .001	.051	-.12	.09	-.07	-.294, .055	.179
SES	-.04	.04	-.06	-.108, .032	.288	.02	.03	.03	-.041, .072	.593
Education	-.08	.05	-.08	-.184, .029	.153	-.06	.04	-.07	-.141, .030	.204
Hiring Experience	.00	.03	.00	-.064, .067	.972	-.01	.03	-.01	-.060, .046	.796
Social desirability	-.01	.01	-.03	-.036, .019	.543	.00	.01	.02	-.019, .025	.773
Model summary	$F(11,304) = 8.13$, $p < .001$, $R^2 = .22$					$F(11,304) = 5.75$, $p < .001$, $R^2 = .17$				

Note. *p* tests are two-tailed. Gender 1 = male. Victim status 1 = Yes.

Supplementary Information H (Study 8): Fusion and willingness to hire among UK nationals

Sample

A sample of $N = 330$ British citizens was recruited via the crowdsourcing platform Prolific. Eight participants were excluded because they did not pass an attention check. The final sample consisted of $N = 327$ participants, ($M_{age} = 42.09$, SD_{age} 11.17), the majority of whom were male (54.7%; 44.6% female, 0.6% prefer to say), and white (92%; 4.9% Asian, 1.8% Mixed, 0.6% Black, 0.6% prefer not to say).

Materials

Participants completed the same measures as for S6, excluding ‘perceived future chances’ and ‘social desirability’. Identity fusion (Cronbach’s $\alpha = .85$), Willingness to hire, political ideology (Cronbach’s $\alpha = .93$), open-mindedness (Cronbach’s $\alpha = .93$), criminal victimisation, contact with formerly incarcerated persons, demographics, socio-economic status, hiring experience, and victim status.

Analyses

Deviation from pre-registration

The association between identity fusion (to a job applicant) and willingness to hire/ perceived future chances of the applicant was originally pre-registered to be tested as part of a larger model i.e., as a mediator between experimental triggers of fusion and reintegration support for the applicant. The evaluation of the experimental effects on identity fusion and indirect effects on reintegration support for an formerly incarcerated person are reported as part of another manuscript (REDACTED, under review). The results reported here do not change when tested as part of the original model. Any overlap in reporting of results has been/will be declared.

Correlational analysis

We first estimated bivariate correlation coefficients (Table H1) before testing predictors of willingness to hire simultaneously via multiple linear regression analyses (Table H2). Results show that fusion to the applicant was the key predictor of willingness to hire, outperforming all other covariates.

Table H1

Descriptive Statistics and Pearson Correlation Coefficients Between Variables of Study 8

	M	SD	N		2.	3.	4.	5.	6.	7.	8.	9.	10.	11.
1. Willingness to hire	4.30	1.03	327	*r*	.41	-.15	-.04	-.14	-.01	.01	-.19	.33	.05	.05
				p	<.001	.007	.475	.012	.876	.834	<.001	<.001	.414	.378
2. Identity fusion	3.07	1.19	327	*r*		-.03	.10	-.09	.02	-.10	-.07	.28	.12	-.03
				p		.589	.079	.097	.686	.073	.204	<.001	.036	.565
3. Age	42.09	11.17	327	*r*			.05	.24	-.01	.23	.21	-.10	.10	.11
				p			.421	<.001	.932	<.001	<.001	.066	.074	.052
4. Gender	0.55	0.50	327	*r*				.07	-.01	.03	.08	.07	.11	.03
				p				.203	.860	.576	.166	.241	.050	.625
5. SES	5.75	1.43	327	*r*					.30	.17	.13	-.04	-.08	-.05
				p					<.001	.002	.022	.463	.158	.346
6. Education	3.92	0.85	327	*r*						-.01	-.08	-.01	-.03	.01
				p						.806	.174	.883	.589	.919
7. Hiring exp	2.04	1.57	327	*r*							.15	.02	.10	.11
				p							.009	.747	.079	.054
8. Ideology	3.97	2.07	327	*r*								-.21	.04	-.02
				p								<.001	.521	.664
9. Open mind	3.87	0.91	327	*r*									.01	-.02
				p									.872	.790
10. Contact prisoner	0.60	0.61	327	*r*										.06
				p										.323
11. Victim of crime	0.71	0.46	327	*r*										
				p										

Note. *p* tests are two-tailed and not adjusted for multiple comparisons.. Gender 1 = male. Victim *status* 1 = Yes.

Table H2

Multiple Linear Regression Analyses Predicting Willingness to Hire Applicant

Variable	Willingness to hire				
	Estimate	SE	β	95% CI [LL, UL]	*p*
Fusion to Applicant	.31	.05	.35	.221, .398	< .001
Open-mindedness	.24	.06	.21	.121, .353	< .001
Political Ideology	-.05	.03	-.10	-.101, .000	.048
Contact with formerly incarcerated persons	.01	.08	.01	-.157, .175	.915
Victim status	.13	.11	.06	-.086, .353	.231
Age	-.01	.01	-.11	-.019, .000	.044
Gender	-.16	.10	-.08	-.362, .039	.113
SES	-.05	.04	-.07	-.124, .029	.222
Education	-.00	.06	-.00	-.126, .118	.949
Hiring Experience	.06	.03	.09	-.006, .127	.075
Model summary	$F(10,315) = 11.35, p < .001, R^2 = .27$				

Note. *p* tests are two-tailed. Gender 1 = male. Victim status 1 = Yes.

Supplementary Information I (Study 9): Fusion and willingness to hire among US nationals

Sample

A sample of N = 330 American citizens was recruited via the crowdsourcing platform Prolific. Ten participants were excluded because they did not pass a manipulation check. The final sample consisted of n = 320 participants, (M_{age} = 44.84, SD_{age} 13.06), the majority of whom were male (56.6%; 42.5% female, 0.6% non-binary/ third gender, 0.3% prefer not to say), and white (82.5%; 6.3% Asian, 5% Black, 3.1% Hispanic, 1.6% Mixed, 1.3% Other, 0.3% Prefer not to say).

Materials

Participants completed the same measures as for S8. Identity fusion (Cronbach's α = .88), Willingness to hire, political ideology (Cronbach's α = .96), open-mindedness (Cronbach's α = .96), criminal victimisation, contact with formerly incarcerated persons, demographics, socio-economic status, hiring experience, and victim status.

Analyses

Deviation from pre-registration

The association between identity fusion (to a job applicant) and willingness to hire/ perceived future chances of the applicant was originally pre-registered to be tested as part of a larger model i.e., as a mediator between experimental triggers of fusion and reintegration support for the applicant. The evaluation of the experimental effects on identity fusion and indirect effects on reintegration support for a formerly incarcerated person are reported as part of another manuscript (REDACTED, under review). The results reported here do not change when tested as part of the original model. Any overlap in reporting of results has been/will be declared.

Correlational analysis

We first estimated bivariate correlation coefficients (Table I1) before testing predictors of willingness to hire simultaneously via multiple linear regression analyses (Table I2). Results show that fusion to the applicant was the key predictor of willingness to hire, outperforming all other covariates.

Table I1

Descriptive Statistics and Pearson Correlation Coefficients Between Variables of Study 9

	M	SD	N		2.	3.	4.	5.	6.	7.	8.	9.	10.	11.
1. Willingness to hire	4.06	1.23	320	*r*	.53	-.09	-.07	-.11	-.02	.11	-.19	.33	.15	.11
				p	<.001	.099	.190	.049	.681	.057	<.001	<.001	.009	.041
2. Identity fusion	2.67	1.30	320	*r*		-.05	-.06	-.05	-.10	.04	-.15	.21	.09	.09
				p		.408	.300	.412	.066	.448	.006	<.001	.112	.098
3. Age	44.84	13.06	320	*r*			-.08	-.01	.03	.25	.09	-.03	.00	.02
				p			.134	.879	.613	<.001	.125	.634	.995	.786
4. Gender	0.57	0.50	320	*r*				.14	.00	.08	.20	-.02	-.00	-.03
				p				.011	.970	.167	<.001	.711	.987	.619
5. SES	5.33	1.73	320	*r*					.47	.12	.06	-.08	-.17	-.10
				p					<.001	.035	.271	.143	.002	.087
6. Education	3.71	1.01	320	*r*						.06	-.05	-.14	-.18	-.07
				p						.293	.406	.012	<.001	.206
7. Hiring exp	2.25	1.65	320	*r*							.01	.09	.16	.08
				p							.843	.128	.005	.162
8. Ideology	4.09	2.96	320	*r*								-.08	.04	-.04
				p								.148	.479	.435
9. Open mind	3.96	1.08	320	*r*									.15	.05
				p									.007	.358
10. Contact prisoner	0.86	0.76	320	*r*										.33
				p										<.001
11. Victim of crime	0.60	0.49	320	*r*										
				p										

Note. *p* tests are two-tailed and not adjusted for multiple comparisons. Gender 1 = male. Victim *status* 1 = Yes.

Table I2

Multiple Linear Regression Analyses Predicting Willingness to Hire Applicant

Variable	Willingness to hire				
	Estimate	SE	β	95% CI [LL, UL]	*p*
Fusion to applicant	.44	.05	.46	.351, .528	< .001
Open-mindedness	.24	.05	.21	.136, .347	< .001
Political ideology	-.03	.02	-.07	-.070, .008	.117
Contact with formerly incarcerated persons	.09	.08	.06	-.070, .249	.272
Victim status	.08	.12	.03	-.161, .316	.523
Age	-.01	.00	-.09	-.017, .001	.074
Gender	-.06	.12	-.02	-.287, .173	.627
SES	-.08	.04	-.12	-.156, -.009	.028
Education	.14	.06	.12	.014, .266	.029
Hiring experience	.07	.04	.09	-.004, .138	.065
Model summary	$F(10,308) = 18.04, p < .001, R^2 = .37$				

Note. *p* tests are two-tailed. Gender 1 = male. Victim status 1 = Yes.

Supplementary Information Reference List

Adler, N. E., Epel, E. S., Castellazzo, G., & Ickovics, J. R. (2000). Relationship of subjective and objective social status with psychological and physiological functioning: Preliminary data in healthy, White women. *Health psychology*, *19*(6), 586.

Copas, J., & Marshall, P. (1998). The offender group reconviction scale: a statistical reconviction score for use by probation officers. *Journal of the Royal Statistical Society Series C: Applied Statistics*, *47*(1), 159-171.

Early, K. W., Chapman, S. F., & Hand, G. A. (2013). Family-focused juvenile reentry services: A quasi-experimental design evaluation of recidivism outcomes. *Journal of Juvenile Justice*, *2*(2), 1-22.

Gómez, Á., Brooks, M. L., Buhrmester, M. D., Vázquez, A., Jetten, J., & Swann, W. B., Jr. (2011). On the nature of identity fusion: Insights into the construct and a new measure. Journal of Personality and Social Psychology, 100(5), 918–933. doi:10.1037/a0022642

Hayes, A. F. (2022). *Introduction to mediation, moderation, and conditional process analysis: A regression-based approach* (3rd ed.). Guilford publications.

Hewson, T., Shepherd, A., Hard, J., & Shaw, J. (2020). Effects of the COVID-19 pandemic on the mental health of prisoners. *The Lancet Psychiatry*, *7*(7), 568-570.

Hirschfield, P. J., & Piquero, A. R. (2010). Normalization and legitimation: Modeling stigmatizing attitudes toward ex-offenders. *Criminology*, *48*(1), 27-55.

Howard, P. (2015). OGRS4: The revised Offender Group Reconviction Scale. In R. Moore (Ed.), *A compendium of research ana analysis on the Offender Assessment System (OASys) 2009-2013* (pp. 152-178). Ministry of Justice Analytical Series July 2015. chrome-extension://efaidnbmnnnibpcajpcglclefindmkaj/https://assets.publishing.service.gov.uk/media/5a7f676fed915d74e33f6380/research-analysis-offender-assessment-system.pdf

HPPS. (2019). *HM Prison & Probation Services.* Incentives Policy Framework. *https://assets.publishing.service.gov.uk/government/uploads/system/uploads/attachment_data/file/1105714/incentives-policy-framework.pdf*

King, G., & Nielsen, R. (2019). Why Propensity Scores Should Not Be Used for Matching. Political Analysis, 27(4), 435–454. https://doi.org/10.1017/pan.2019.11

Klingemann, H. D. (1997). The left-right self-placement question in face to face and telephone surveys. In W. E. Saris, & M. Kaase (Eds.), Eurobarometer: measurement instruments for opinions in Europe (pp. 100-110). Mannheim: Zentrum für Umfragen, Methoden und Analysen. https://nbn-resolving.org/urn:nbn:de:0168-ssoar-50511-4

Levy, S. R., Stroessner, S. J., & Dweck, C. S. (1998). Stereotype formation and endorsement: The role of implicit theories. *Journal of personality and social psychology*, *74*(6), 1421.

McCaffrey, D. F., Griffin, B. A., Almirall, D., Slaughter, M. E., Ramchand, R., & Burgette, L. F. (2013). A tutorial on propensity score estimation for multiple treatments using generalized boosted models. *Statistics in medicine*, *32*(19), 3388-3414.

McDougall, C., Pearson, D. A., Torgerson, D. J., & Garcia-Reyes, M. (2017). The effect of digital technology on prisoner behavior and reoffending: a natural stepped-wedge design. *Journal of Experimental Criminology*, *13*(4), 455-482.Postmes, T., Haslam, S. A., & Jans, L. (2013). A single-item measure of social identification: Reliability, validity, and utility. *British journal of social psychology*, *52*(4), 597-617.

Postmes, T., Haslam, S. A., & Jans, L. (2013). A single-item measure of social identification: Reliability, validity, and utility. *British journal of social psychology*, *52*(4), 597-617.

Ridgeway, G., McCaffrey, D., Morral, A., Cefalu, M., Burgette, L., Pane, J., & Griffin, B. A. (2021). Toolkit for Weighting and Analysis of Nonequivalent Groups: A guide to the twang package. *Vignette*, *2021*, 26.

Shields, I. W., & Simourd, D. J. (1991). Predicting predatory behavior in a population of incarcerated young offenders. *Criminal Justice and Behavior*, *18*(2), 180-194.

Stöber, J. (2001). The Social Desirability Scale-17 (SDS-17): Convergent validity, discriminant validity, and relationship with age. *European Journal of Psychological Assessment*, *17*(3), 222.

Suhomlinova, O., Ayres, T. C., Tonkin, M. J., O'Reilly, M., Wertans, E., & O'Shea, S. C. (2022). Locked up while locked down: Prisoners' experiences of the COVID-19 pandemic. *The British Journal of Criminology*, *62*(2), 279-298.

Swann, W. B. Jr., Gómez, A., Seyle, D. C., Morales, J., and Huici, C. (2009). Identity fusion: the interplay of personal and social identities in extreme group behavior. *J. Personal. Soc. Psychol.* 96:995. doi: 10.1037/a0013668
