# Supplementary material for: A soccer-based intervention improves incarcerated individuals’ behaviour and public acceptance through group bonding
Source: Nat Hum Behav. 2024 Oct 14;8(12):2304–13. doi: 10.1038/s41562-024-02006-3 (PMC11659165; doi:10.1038/s41562-024-02006-3)
Supplement: Supplementary file 2 — Reporting Summary [file 41562_2024_2006_MOESM2_ESM.pdf]

nature portfolio

Corresponding author(s): Martha Newson

Last updated by author(s): Jul 25, 2024

Reporting Summary

Nature Portfolio wishes to improve the reproducibility of the work that we publish. This form provides structure for consistency and transparency in reporting. For further information on Nature Portfolio policies, see our Editorial Policies and the Editorial Policy Checklist.

Statistics

For all statistical analyses, confirm that the following items are present in the figure legend, table legend, main text, or Methods section.

n/a	Confirmed	
☐	☒	The exact sample size (*n*) for each experimental group/condition, given as a discrete number and unit of measurement
☐	☒	A statement on whether measurements were taken from distinct samples or whether the same sample was measured repeatedly
☐	☒	The statistical test(s) used AND whether they are one- or two-sided<br>*Only common tests should be described solely by name; describe more complex techniques in the Methods section.*
☐	☒	A description of all covariates tested
☐	☒	A description of any assumptions or corrections, such as tests of normality and adjustment for multiple comparisons
☐	☒	A full description of the statistical parameters including central tendency (e.g. means) or other basic estimates (e.g. regression coefficient) AND variation (e.g. standard deviation) or associated estimates of uncertainty (e.g. confidence intervals)
☐	☒	For null hypothesis testing, the test statistic (e.g. *F*, *t*, *r*) with confidence intervals, effect sizes, degrees of freedom and *P* value noted<br>*Give P values as exact values whenever suitable.*
☒	☐	For Bayesian analysis, information on the choice of priors and Markov chain Monte Carlo settings
☐	☒	For hierarchical and complex designs, identification of the appropriate level for tests and full reporting of outcomes
☐	☒	Estimates of effect sizes (e.g. Cohen's *d*, Pearson's *r*), indicating how they were calculated

Our web collection on statistics for biologists contains articles on many of the points above.

Software and code

Policy information about availability of computer code

Data collection	No software or code was used to collect data.
Data analysis	The following statistical software packages were used: Rstudio v.2023.06.1 (package TWANG v.2.6), SPSS v. 28 (Hayes 2022 PROCESS macro v.4.2). Analysis code is available from: https://osf.io/u74bf/?view_only=d27b1d321455470d8dab9a5ae0932029.

For manuscripts utilizing custom algorithms or software that are central to the research but not yet described in published literature, software must be made available to editors and reviewers. We strongly encourage code deposition in a community repository (e.g. GitHub). See the Nature Portfolio guidelines for submitting code & software for further information.

Data

Policy information about availability of data

All manuscripts must include a data availability statement. This statement should provide the following information, where applicable:

- Accession codes, unique identifiers, or web links for publicly available datasets
- A description of any restrictions on data availability
- For clinical datasets or third party data, please ensure that the statement adheres to our policy

For S1-S2, prison data is controlled by HMPPS and requests to obtain it should be directed to HMPPS following National Research Committee (NRC) approvals, https://apply-for-hmpps-research.service.justice.gov.uk/Introduction-and-Guidance/. Survey data for this population, which was collected by the researchers, can be obtained from the research team on request.

Data that is not on OSF will be held by the researchers for 10 years as per standard University retention guidelines.
Datasets and output generated for studies 3–9 can be found on OSF at https://osf.io/u74bf/?view_only=d27b1d321455470d8dab9a5ae0932029.

Research involving human participants, their data, or biological material

Policy information about studies with human participants or human data. See also policy information about sex, gender (identity/presentation), and sexual orientation and race, ethnicity and racism.

Reporting on sex and gender	Findings from S1-2 apply to a male population only, which is explained in the SI. The use of gender as a covariate in S3-9 is justified based on established evidence for gender differences in the perception of persons involved in the criminal justice system.
Reporting on race, ethnicity, or other socially relevant groupings	We report the distribution of samples' ethnic background composition in all studies.<br>We include ethnicity as a matching criteria & covariate in Study 1, which is necessary to accurately conduct the comparison group analyses.
Population characteristics	Studies 1-3 include vulnerable groups (prisoners, ex-prisoners). Studies 3-9 include members of the general population. For sample characteristics, see study design reporting.
Recruitment	Samples for S1-2 were recruited from the UK prison population. Samples for S3-9 were recruited from the crowdsourcing platform Prolific. Details on recruitment and reimbursement are provided in the manuscript.
Ethics oversight	S1-2 were approved by the ethics board of the University of Oxford and the National Reseach Committee. S3-9 were approved by the ethics board of the School of Anthropology and Connservation at the University of Kent. Details are provided in the manuscript.

Note that full information on the approval of the study protocol must also be provided in the manuscript.

Field-specific reporting

Please select the one below that is the best fit for your research. If you are not sure, read the appropriate sections before making your selection.

☐ Life sciences ☒ Behavioural & social sciences ☐ Ecological, evolutionary & environmental sciences

For a reference copy of the document with all sections, see nature.com/documents/nr-reporting-summary-flat.pdf

Behavioural & social sciences study design

All studies must disclose on these points even when the disclosure is negative.

Study description	Studies are labelled according to their research design: S1 (quasi-experimental), S2-3, 5-9 (cross-sectional), S4 (experimental)
Research sample	Samples for S1 were drawn from the UK prison poulation (provided by the UK Ministry of Justice, from their database P-Nomis), including a group of prisoners who took part in the Twinning Project between 09/2021 and 03/2023 (n = 676, Mage = 31.17, s.d.age = 7.75, White = 59%, Black = 22%, Asian = 7%, Mixed = 9%, Other = 1%, Unknown = 2%) and a control group of prisoners who did not take part. Treatment group (n = 1874, Mage = 34.49, s.d.age = 10.53, White = 73%, Black = 11%, Asian = 8%, Mixed = 5%, Other = 1%, Unknown = 0%).<br>The sample of S2 consisted of people who took part in the Twinning Project in S1 and who took part in a longitudinal survey (n = 388, Mage = 30.20, s.d.age = 7.12, White = 60%, Black = 20%, Asian = 9%, Mixed = 10%, Other = 2%).<br>The samples for S3-9 were recruited via the crowdsourcing platform Prolific, and distinct recruitment are specified.<br>S3, US and UK nationals who have served time in prison (recruited N = 250, final sample N = 249, Mage = 43.88, SDage = 11.63, background US = 66.3%, male (70% male, 27% female, 2% non-binary/third gender, white = 82%, Black = 11%, Asian = 3%, Mixed = 2%, Other = 2%, Unknown = 0%).<br>S4, UK nationals with and without interest in soccer (recruited N = 282, final sample N = 234, 47% soccer fans, Mage = 44.52, SDage 14.05, 65.4% male; 34.2% female; 0.4% non-binary/third gender; 90.2% white, 5.1% Asian, 2.1% Black, 2.1% Mixed, 0.4% Unknown).<br>S5, UK nationals with interest in soccer (recruited N = 331, final sample N = 303; Mage = 41.12, SDage 14.25; 80.5% male; 18.8% female, 0.7% prefer not to say; 82.5% white; 9.2% Asian, 4% Mixed, 1.7% Black, 1% Other, 0.3% North African, 1.3% Unknown).<br>S6, US nationals with an interest in American Football (recruited N = 330, final sample N = 294; Mage = 40.92, SDage 12.47; 96.9% male, 2.4% female, 0.3% non-binary/third gender, 0.3% prefer not to say; 76.2% white, 7.1 % Black, 7.1% Hispanic, 4.1% Mixed, 3.7% Asian, 1.4% Other, 0.3% prefer not to say).<br>S7, US nationals born before 1995 (recruited N = 333, final sample N = 319; Mage = 42.49, SDage 11.69; 57.7% female, 41.4% male, 0.6% non-binary, 0.3% prefer not to say; 74% white,7.8% Black, 6.3% Hispanic, 5% Asian, 3.4% Mixed, 2.5% Other, 0.9% Prefer not to say).<br>S8, UK nationals (recruited N = 330, final sample N = 327; Mage = 42.09, SDage 11.17); 54.7% male, 44.6% female, 0.6% prefer to say); 92% white, 4.9% Asian, 1.8% Mixed, 0.6% Black, 0.6% prefer not to say).<br>S9, US nationals (recruited N = 330, final sample N = 320, Mage = 44.84, SDage 13.06; 56.6% male, 42.5% female, 0.6% non-binary/third gender, 0.3% prefer not to say; 82.5% white, 6.3% Asian, 5% Black, 3.1% Hispanic, 1.6% Mixed, 1.3% Other, 0.3% Prefer not to say).<br>No samples were representative.

Sampling strategy	Samples for Studies 1 and 2 were recruited based on a pre-determined timeframe, i.e., prisoners who took part in the Twinning Project between 09/2021 and 03/2023. The maximum available data was collected. No statistical methods were used to pre-determine sample sizes, but our sample sizes are larger than those reported in previous publications with comparable study designs (e.g., Kovalsky et al. 2021; McDavid et al., 2019), and larger than samples require for PSM approaches to replicate RCT results (Campbell & Labrecque, 2022).<br>Data for Studies 3 to 9 were convenience samples collected using the online crowdsourcing platform Prolific. Individual samples from the UK and US were recruited between January and May 2023, and participants received on average the equivalent of £9.00 per hour in financial reimbursement for taking part. Sample sizes were determined by a priori power analyses in G*Power (Faul et al., 2009) to detect small-moderate effect sizes in linear regression/ANOVA analyses (i.e., f2 = 0.08/0.09, f = 0.18) at the standard .05 error probability at .80 power.
Data collection	For S1-2 data was collected via a third party (HMPPS). Prison behaviour data which was provided via the MoJ database P-Nomis reflect routine observations and records compiled by prison staff. The longitudinal surveys used for S2 were administered using pen-and-paper questionnaires by prison officers and intervention provider staff who received specialist training for data collection. These surveys were completed in the respective programme facilities during the first and last session of the intervention, and two months after completing the intervention in prisoner's cells. Paper copies of surveys were later digitised for further analsyes by the research team. For studies 3-9, data was collected using online questionnaires, distributed via the crowdsourcing platform Prolific and using the online survey platform Qualtrics. For the experimental study 4, experimental conditions were displayed in randomised order using Qualtric's block-randomisation feature.
Timing	Data for studies 1-2 was collected between 09/2021 and 03/2023, and data for studies S3-9 was collected between 01/2023 and 05/2023.
Data exclusions	For S1-2 available data of n = 162 cases were excluded from all analyses (67 due to unconfirmed age >18, 93 female cases) and a further 158 cases were excluded from analyses containing case-note variables, due to incomplete data recording. For S3-9, data was removed based on data quality checks (details are reported in the manuscript and SI). The number of exclusions was: S3 = 1, S4 = 48; S5 = 28; S6 = 36; S7 = 14; S8 = 8; S9 = 10.
Non-participation	For studies 1-2, details about attrition in the longitudinal studies is provided in the manuscript. For online studies (S3-9) this is not relevant.
Randomization	In study 4, participants were randomly presented with stimuli of a within-subject experimental conditions using a block-randomisation function of the online survey software Qualtrics. There were no randomised elements in the assignment to treatment groups or data collection for Studies 1 and 2 which reflect restrictions in the programme delivery and data collection. There was no randomisation in any of the other studies (3, 5-9). Covariates were used to match treatment and control groups using a propensity score weighting approach in S1 (details are reported in the manuscript and SI), and covariates are included (held constant) in all final analyses for S1-9.

Reporting for specific materials, systems and methods

We require information from authors about some types of materials, experimental systems and methods used in many studies. Here, indicate whether each material, system or method listed is relevant to your study. If you are not sure if a list item applies to your research, read the appropriate section before selecting a response.

Materials & experimental systems

n/a	Involved in the study	
☒	☐	Antibodies
☒	☐	Eukaryotic cell lines
☒	☐	Palaeontology and archaeology
☒	☐	Animals and other organisms
☒	☐	Clinical data
☒	☐	Dual use research of concern
☒	☐	Plants

Methods

n/a	Involved in the study	
☒	☐	ChIP-seq
☒	☐	Flow cytometry
☒	☐	MRI-based neuroimaging

Plants

Seed stocks	*Report on the source of all seed stocks or other plant material used. If applicable, state the seed stock centre and catalogue number. If plant specimens were collected from the field, describe the collection location, date and sampling procedures.*
Novel plant genotypes	*Describe the methods by which all novel plant genotypes were produced. This includes those generated by transgenic approaches, gene editing, chemical/radiation-based mutagenesis and hybridization. For transgenic lines, describe the transformation method, the number of independent lines analyzed and the generation upon which experiments were performed. For gene-edited lines, describe the editor used, the endogenous sequence targeted for editing, the targeting guide RNA sequence (if applicable) and how the editor was applied.*
Authentication	*Describe any authentication procedures for each seed stock used or novel genotype generated. Describe any experiments used to assess the effect of a mutation and, where applicable, how potential secondary effects (e.g. second site T-DNA insertions, mosiacism, off-target gene editing) were examined.*
